# CLC-Pred 2.0: A Freely Available Web Application for In Silico Prediction of Human Cell Line Cytotoxicity and Molecular Mechanisms of Action for Druglike Compounds

**DOI:** 10.3390/ijms24021689

**Published:** 2023-01-14

**Authors:** Alexey A. Lagunin, Anastasia V. Rudik, Pavel V. Pogodin, Polina I. Savosina, Olga A. Tarasova, Alexander V. Dmitriev, Sergey M. Ivanov, Nadezhda Y. Biziukova, Dmitry S. Druzhilovskiy, Dmitry A. Filimonov, Vladimir V. Poroikov

**Affiliations:** 1 Department of Bioinformatics, Institute of Biomedical Chemistry, 119435 Moscow, Russia; 2 Department of Bioinformatics, Pirogov Russian National Research Medical University, 117997 Moscow, Russia

**Keywords:** cytotoxicity, cell lines, mechanism of action, in silico prediction, SAR, PASS, CLC-Pred

## Abstract

In vitro cell-line cytotoxicity is widely used in the experimental studies of potential antineoplastic agents and evaluation of safety in drug discovery. In silico estimation of cytotoxicity against hundreds of tumor cell lines and dozens of normal cell lines considerably reduces the time and costs of drug development and the assessment of new pharmaceutical agent perspectives. In 2018, we developed the first freely available web application (CLC-Pred) for the qualitative prediction of cytotoxicity against 278 tumor and 27 normal cell lines based on structural formulas of 59,882 compounds. Here, we present a new version of this web application: CLC-Pred 2.0. It also employs the PASS (Prediction of Activity Spectra for Substance) approach based on substructural atom centric MNA descriptors and a Bayesian algorithm. CLC-Pred 2.0 provides three types of qualitative prediction: (1) cytotoxicity against 391 tumor and 47 normal human cell lines based on ChEMBL and PubChem data (128,545 structures) with a mean accuracy of prediction (AUC), calculated by the leave-one-out (LOO CV) and the 20-fold cross-validation (20F CV) procedures, of 0.925 and 0.923, respectively; (2) cytotoxicity against an NCI60 tumor cell-line panel based on the Developmental Therapeutics Program’s NCI60 data (22,726 structures) with different thresholds of IG_50_ data (100, 10 and 1 nM) and a mean accuracy of prediction from 0.870 to 0.945 (LOO CV) and from 0.869 to 0.942 (20F CV), respectively; (3) 2170 molecular mechanisms of actions based on ChEMBL and PubChem data (656,011 structures) with a mean accuracy of prediction 0.979 (LOO CV) and 0.978 (20F CV). Therefore, CLC-Pred 2.0 is a significant extension of the capabilities of the initial web application.

## 1. Introduction

Cancer is a major public health problem worldwide [1] and the discovery of new antitumor agents is one of the main directions of modern drug development. Despite significant efforts in the new antitumor agent research, due to the great diversity of tumors, their individual peculiarities and the emergence of drug resistance, there is a great need for new antitumor compounds. In vitro screening cytotoxicity of drug candidates against cell lines significantly improves the process of drug discovery. It allows the study of the efficacy against tumor cell lines and the estimation toxicity in normal cell lines to select the most effective and safe compounds. At the present time, over a thousand cell lines are used in the study of cytotoxicity (e.g., the Center for Molecular Therapeutics included a panel with 1200 human cancer cell lines [2]). NCI60 is one of the first known panels of tumor cell lines which has been used for the screening of antitumor drugs [3]. Even though cell lines have been used to assess cytotoxicity for several decades, only a few hundred thousand compounds have been tested so far. Many experimental results of cell-line cytotoxicity studies are kept in freely available chemical databases (e.g., PubChem [4] and ChEMBL [5]) or sources related to some screening programs (e.g., the Genomics of Drug Sensitivity in Cancer project [6] or Developmental Therapeutics Program NCI60 [7]). They can be used to create in silico tools for the prediction of cell-line cytotoxicity that are based on the analysis of structure–activity relationships (SAR). Such tools are supposed to estimate the potential cytotoxic effects of drug candidates or new druglike compounds before their experimental studies. They may also be of help in the studies of natural compounds and drug repositioning.

A number of studies aimed at creating computational tools for the prediction of cell-line cytotoxicity based on structural formulae of compounds, but most of them either were limited by the narrow chemical space due to the small number of compounds used [8,9] or made prediction for the cell lines related to the appropriate tissue [10,11]. Therefore, in 2018 we introduced a freely available web application, CLC-Pred (Cell-Line Cytotoxicity Predictor, https://www.way2drug.com/cell-line/, accessed on 1 January 2023), for the in silico prediction of cytotoxic effect of compounds in 278 cancer and 27 normal cell lines based on their structural formula [12]. It was based on the PASS Online approach [13,14,15] and the experimental data from ChEMBL’s 23 databases for 59,882 compounds. Since then, more than 80 papers have been published with studies using CLC-Pred to evaluate the cytotoxicity of new chemical compounds [16,17], natural compounds [18,19,20], as well as in studies aimed at finding new antitumor substances [21,22]. In the present paper, we offer a new version of the web application, CLC-Pred 2.0, that is based on significantly bigger data on the cytotoxicity of compounds in relation to cell lines. We also extended the capabilities of our web application by adding the ability to predict cytotoxicity with different thresholds of GI_50_ data (100, 10 and 1 nM) against NCI60 cell lines based on the data from the Developmental Therapeutics Program (DTP) NCI60 [7], where more than 20,000 compounds were tested against 60 cell lines using a single protocol. Moreover, we added the ability to predict over 2,100 molecular mechanisms of action (MoA) in relation to human proteins, which, in combination with the phenotypic assessment of cellular cytotoxicity, will allow a better planning of experimental studies and suggest possible molecular mechanisms of cytotoxicity for the studied compounds.

## 2. Results

### 2.1. Creation of Classification Models for Cell-Line Cytotoxicity Prediction

**Figure 1:**
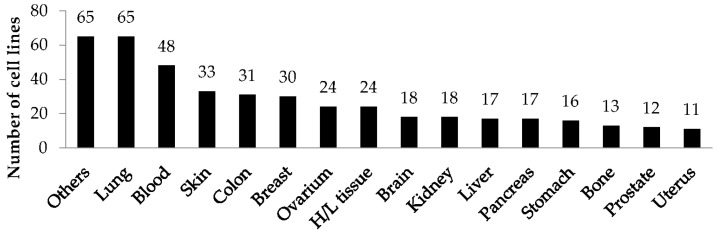
Distribution of 438 cell lines by organs and tissue.

The training set with 128,545 unique structures of compounds tested against 1162 cell lines was created based on the experimental data (379,767 experimental values) related to cytotoxic studies from the ChEMBL and PubChem databases. After PASS training and the creation of classification models of “structure–cytotoxicity” relationships, 438 human cell lines (391 tumor and 47 normal cell lines) with an accuracy of prediction (AUC) higher than 0.8 were selected (Table S1). Most of the cell lines were related to lung, blood, skin, colon, breast, ovarian, hematopoietic and lymphoid tissues (Figure 1).

The average accuracy of prediction (AUC) calculated by the leave-one-out (LOO CV) and 20-fold cross-validation (20-fold CV) procedures was 0.925 and 0.923, respectively. It displays the robustness of the cytotoxicity prediction for the selected cell lines.

**Figure 2:**
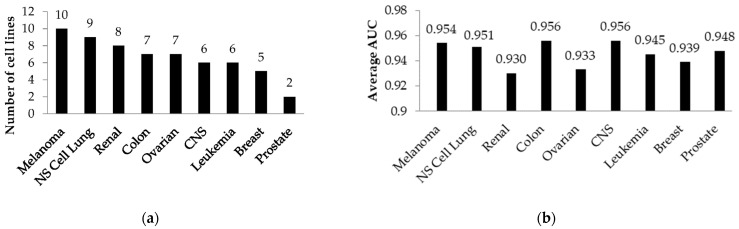
(**a**) Distribution of cell lines from DTP NCI60 by organs or general types; (**b**) average accuracy of cytotoxicity prediction for grouped cell lines from DTP NCI60 at a 1 nM IG_50_ value threshold. NS Cell Lung—nonsmall-cell lung.

The training sets based on the DTP’s NCI60 data included 22,726 unique structures tested against 60 cell lines with 1,262,878 experimental values. These training sets were created based on different thresholds of IG_50_ values (100, 10 and 1 nM). The average AUC calculated by the LOO CV and 20-fold CV procedures was (1) 0.870 and 0.869 for 100 nM; (2) 0.898 and 0.897 for 10 nM; (3) 0.945 and 0.942 for a 1 nM threshold, respectively. The accuracy of the cytotoxicity prediction against all NCI60 cell lines was higher than 0.80. In contrast to the previous training set based on ChEMBL and PubChem data, in these training sets, the differentiation between active and inactive compounds was made according to the nanomolar thresholds of GI_50_ values. The number of cell lines and the average accuracy of prediction (for a 1 nM IG_50_ value threshold) according to the origin are shown in Figure 2a and Figure 2b, respectively. Most cell lines were associated with melanoma, nonsmall-cell lung carcinoma and kidneys. The most accurate SAR models were received for cell lines from the central nervous system (CNS), colon and melanoma. The detailed data on the prediction accuracy, the number of active compounds and other characteristics from CellMiner DTP NCI60 metadata for appropriate cell lines are presented in Supplementary Materials, Table S2.

### 2.2. Creation of Classification Models to Predict Molecular Mechanisms of Action

**Figure 3:**
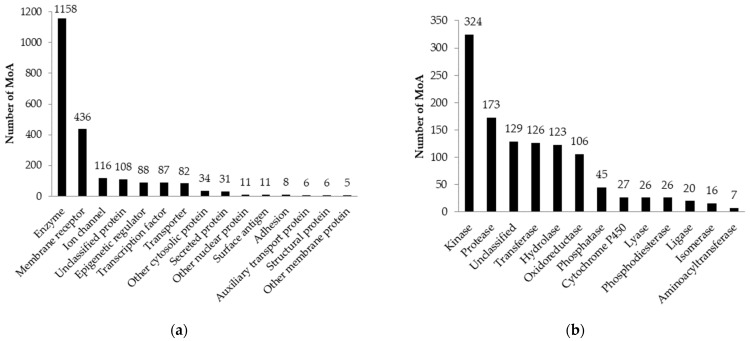
Distribution of predicted mechanisms of action by protein families: (**a**) number of predicted MoA classifying by ChEMBL protein family level 1; (**b**) number of predicted MoA classified by ChEMBL protein family level 2 for the enzyme protein family.

The training set to predict the molecular mechanisms of action related to the interaction of compounds with human proteins was created based on ChEMBL and PubChem data. The training set included 656,011 unique structures and 957,545 records describing experimental results related to 2876 molecular mechanisms of action. After completing the training procedure and selection of activities with the accuracy of prediction over 0.8, 2170 molecular mechanisms of action were selected. The average accuracy of the prediction calculated by the LOO CV and the 20-fold CV procedures was 0.979 and 0.978, respectively. The distribution of the mechanisms of action based on the ChEMBL family classification of proteins is shown in Figure 3. Most MoA are related to enzymes, membrane receptors (mostly G protein-coupled receptors) and ion channels (Figure 3a). Most enzymes are kinases, proteases, transferases and hydrolases (Figure 3b). The detailed data on the training set, the accuracy of prediction, numbers of active compounds and ChEMBL protein family classifications are presented in Supplementary Materials, Table S3.

### 2.3. CLC-Pred 2.0 Web Application

**Figure 4:**
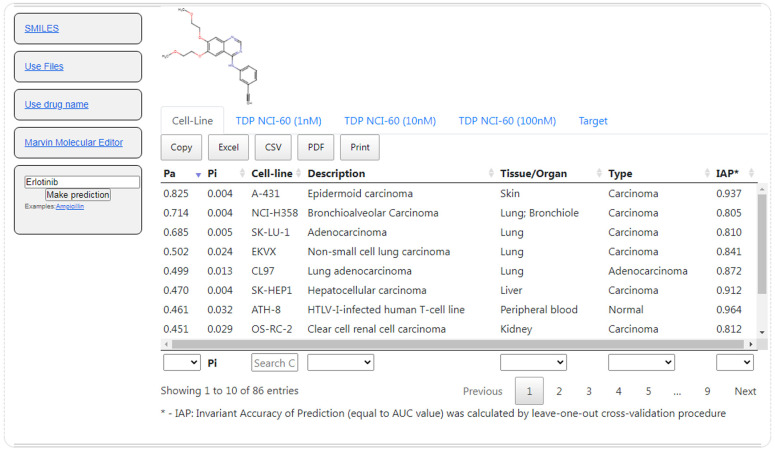
Interface of CLC-Pred 2.0 and prediction results of general cell-line cytotoxicity for erlotinib.

The CLC-Pred 2.0 web application (https://www.way2drug.com/CLC-pred, accessed on 1 January 2023) was developed based on the created SAR models for the prediction of human cell lines’ cytotoxicity and molecular mechanisms of actions related to human proteins. The web application allows one to use SMILES, the names of approved drugs, MDL Molfile or Marvin JS drawn structures as an input. The prediction results consist of three tabs—“Cell-line”, ”TDP NCI-60 (1 nM)”, ”TDP NCI-60 (10 nM)”, ”TDP NCI-60 (100 nM)” and ”Target”. Inside each tab, the user can see the results of the prediction with selectable rows. The results of the prediction contain three main characteristics, the names of activities, *Pa* (the probability that the compound will be active) and *Pi* (the probability that the compound will be inactive). In addition to these characteristics, one can see the detailed description of the activity. The prediction results are downloadable as PDF, CSV, Excel files and can be copied to the clipboard. In the section “Training set”, the user sees the total list of the predicted activities with the accuracy of prediction calculated by the LOO CV and 20-fold CV procedures. The interface of CLC-Pred 2.0 with prediction results for the EGFR inhibitor erlotinib is shown on Figure 4. According to Drugbank [23], erlotinib is used in the treatment of nonsmall-cell lung cancer, pancreatic cancer and several other types of cancer. The prediction results also demonstrate high and medium probabilities of cytotoxicity against EKVX (nonsmall-cell lung carcinoma) and several other cell lines related to the lungs (NCI-H358, SK-LU-1 and CL97).

**Figure 5:**
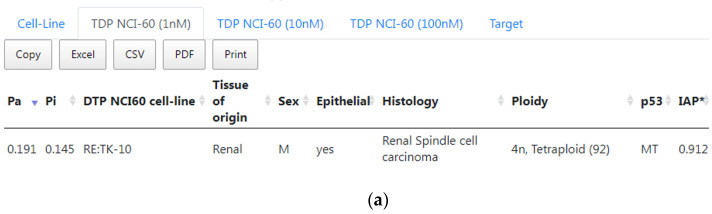

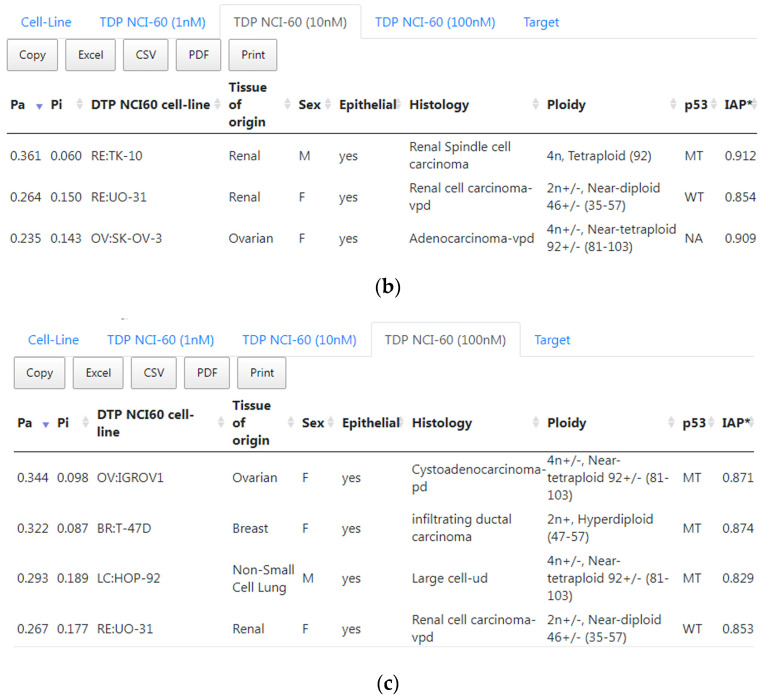
CLC-Pred 2.0 results of cytotoxicity prediction based on the TDP’s NCI60 data for erlotinib at different threshold of cytotoxicity: (**a**) at 1 nM, (**b**) at 10 nM; (**c**) at 100 nM.

The prediction results of cell-line cytotoxicity based on the TDP’s NCI-60 data for erlotinib showed that it had the potential to be very active against renal carcinoma cell line TK-10 at 1 and 10 nM (Figure 5a,b). The cytotoxicity against TK-10 was also predicted at 100 nM threshold (it is not displayed in Figure 5c because *Pa* = 0.255). Figure 5c shows the prediction of the cytotoxicity against another kidney cell line—UO-31 (*Pa* = 0.267). Such results also coincide with the prediction of the medium probability to reveal cytotoxicity in the kidney sarcoma cell line OS-RC-2 (Figure 4). The successful treatment of kidney cancer patients with the combination of erlotinib and bevacizumab was also confirmed by a recent publication [24]. The use of erlotinib in kidney cancer patients was also proposed by the independent bioinformatics analysis of OMICS data [25].

**Figure 6:**
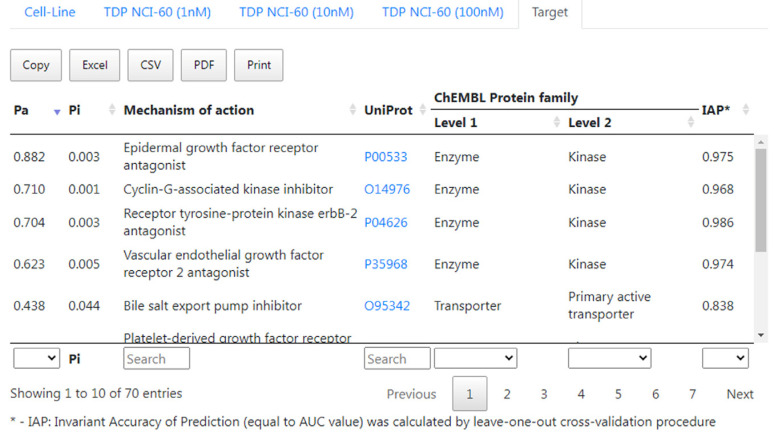
Interface of CLC-Pred 2.0 with prediction results of molecular mechanisms of action for erlotinib.

The prediction results for possible molecular mechanisms of action include the prediction of EGFR antagonism, which is the main mechanism of the anticancer activity of erlotinib, as seen in Figure 6, wherein the value of the probability for erlotinib to be an EGFR antagonist is higher than the one for other predicted mechanisms of action. One can also see that erlotinib has a high probability to act as an inhibitor of several kinases. Such probability may become the basis for studying experimentally the interaction of erlotinib with the appropriate kinases.

## 3. Discussion

In this study, we significantly expanded the capabilities of CLC-Pred web application. The number of structures of tested compounds studied for cell-line cytotoxicity and used for the creation of SAR models was increased by up to two times. The number of cell lines, both tumor and normal, for which the cytotoxicity of compounds can be predicted, was almost doubled. The data on cell-line cytotoxicity based on the TDP’s NCI60 provided the opportunity to create highly accurate SAR models and search for very active compounds with different thresholds of activity: 1, 10 and 100 nM of GI_50_ values. In CLC-Pred 2.0, the toxicity prediction coupled with the ability to predict molecular mechanisms of action enables researchers to obtain both the phenotypic information and molecular effects of the studied compounds. The prediction of both cell-line cytotoxicity and known mechanisms of antineoplastic action considerably increases the possibility to find anticancer compounds. Moreover, the prediction of the molecular mechanisms of action will help to estimate the possible action of the studied compound on the proteins related to ADME and side effects. CLC-Pred 2.0 provides the possibility to predict more than 2100 mechanisms of action. The prediction of cell-line cytotoxicity against normal cells will help to evaluate the possible toxic effect of the compounds studied in different therapeutic fields, with the treatment outcomes for patients with a tumor included. We believe that CLC-Pred 2.0 will also add to the studies related to drug repositioning and extracts. The results of the prediction provided by CLC-Pred 2.0 will help medicinal chemists, pharmacologists and toxicologists to select more promising drug candidates and plan experimental studies based only on the structural formula of compounds. It will lead to a more rational use of time and resources in drug development.

## 4. Materials and Methods

### 4.1. Datasets

#### 4.1.1. Creation of the Training Set with Tumor and Normal Cell Lines’ Cytotoxicity Data

The experimental data (IG_50_, IC_50_ and % inhibition values) and structural formulas related to cell-line cytotoxicity studies were extracted from the PubChem (February 2022) and ChEMBL (version 29) databases. The compounds were considered active if their IG_50_ or IC_50_ values were less than 10,000 nM or if the percent of inhibition exceeded 50%. All compounds were considered as inactive for the appropriate cell line if they were not active for the cell line according to the above-mentioned criteria. The names of cell lines were standardized. Cell lines’ descriptions, tissue specificity and tumor types were used according to ChEMBL, Cellosaurus and ATCC (American Type Culture Collection) data.

#### 4.1.2. Creation of Training Set Based on the DTP’s NCI60 Data

The initial data from CellMiner [7] on cytotoxicity assessment according to drug activity levels expressed as 50% growth-inhibitory levels (GI_50_) at 48 h using the sulforhodamine B assay [26] were used for the preparation of the DTP’s NCI60 training sets. All data with labels in the column “Failure reason” were deleted. The compounds were considered active against the appropriate cell line if its medium negative log10 of the GI_50_ value was equal or over 9 (1 nM), 8 (10 nM) or 7 (100 nM) for the appropriate training sets. Additional data related with the characteristics of NCI60 cell lines were given from the table with the metadata on the NCI60 panel of cancer cell lines that is available on CellMiner.

#### 4.1.3. Creation of Training Set with Data on Mechanisms of Action

The experimental data (K_i_, IC_50_, K_act_ and % inhibition values) and structural formulas related to the action on human proteins were extracted from the PubChem (February 2022) and ChEMBL (version 29) databases. The compounds were considered active if their K_i_ or IC_50_ values were less than 10,000 nM or if the percent of inhibition was higher than 50%. All compounds were considered inactive for the appropriate mechanism of action if they were not active according to the above-mentioned criteria. The canonical names of proteins from the UniProt database were used to create the names of the mechanisms of action. The data for protein targets from PubChem and ChEMBL were coupled based on UniProt IDs.

### 4.2. PASS Approach

The PASS (Prediction of Activity Spectra for Substances) software tool simultaneously predicts many types of biological activities (activity spectra) for compounds based on their structures [12,13,14,15]. Biological activities are described qualitatively (active or inactive) in PASS. The cytotoxicity of compounds against the appropriate cell lines and the mechanisms of action are also considered as biological activities. The sets of unique substructural atom-centric Multilevel Neighborhoods of Atoms (MNA) descriptors are used for the representation of the molecular structures of compounds. These descriptors are a linear notation of atom-centered fragments in the structure of an organic molecule. They are based on the molecular structure representation that includes the hydrogen atoms according to the valences and partial charges of the atoms and does not specify the types of bonds. MNA descriptors do not represent the stereochemical peculiarities of the molecule, the substances with stereochemical different structures are formally considered to be equivalent [15].

The algorithm for revealing structure–activity relationships is based on the naive Bayes approach with some significant enhancements [14,15].

The leave-one-out and 20-fold cross-validation procedures for all predictable types of biological activity and all substances are used during the training procedure. The invariant accuracy of prediction (IAP) is calculated for accuracy estimations. IAP values are numerically equal to ROC AUC (the area under the ROC curve) values. IAP is the estimation of the probability that positive and negative examples (active and inactive compounds) that are arbitrarily chosen from a validation set may be classified correctly by the prediction.

The prediction results of PASS are given as a list of activities with probabilities “to be active” *Pa* and “to be inactive” *Pi*. The list of predicted activities is arranged in a descending order according to *Pa–Pi* values. Thus, the more probable activity types are put at the top of the list. If the user chooses a higher *Pa* value as a cutoff for the selection of probable activities, the chance to confirm the predicted activities by the experiment is also high; however, many existing activities will be lost. For instance, if *Pa* > 0.5 is used as a threshold, about a half of the real activities will be lost; for *Pa* > 0.7, the portion of lost activities is 70%, etc. Moreover, a high *Pa* value shows the similarity of the queried compound structure and the typical compound structures of similar activity. Thus, experimental studies are mostly likely to confirm such an activity. The *Pa* value being smaller but still higher than the *Pi* value means that the chance to confirm the activity is lower. However, if the activity is discovered in the experiment, the compounds may be a new chemical entity for it.

## Data Availability

Not applicable.

## Supplementary Materials

The following supporting information can be downloaded at: https://www.mdpi.com/article/10.3390/ijms24021689/s1.
