# Supplementary material for: CLC-Pred 2.0: A Freely Available Web Application for In Silico Prediction of Human Cell Line Cytotoxicity and Molecular Mechanisms of Action for Druglike Compounds
Source: Int J Mol Sci. 2023 Jan 14;24(2):1689. doi: 10.3390/ijms24021689 (PMC9861947; doi:10.3390/ijms24021689)
Supplement: Supplementary file 1 [file ijms-24-01689-s001.zip › Table S3.pdf]

Table S3: The information related with description of the training set based on ChEMBL and PubChem data and accuracy of prediction for 2170 molecular mechanisms of action.

Activity Type	UniProt ID	Number	IAP, LOO	IAP, 20-Fold	ChEMBL protein family level 1	ChEMBL protein family level 2
[Pyruvate dehydrogenase (acetyl-transferring)] kinase isozyme 1, mitochondrial inhibitor	Q15118	598	0.9787	0.979	Enzyme	Kinase
[Pyruvate dehydrogenase (acetyl-transferring)] kinase isozyme 2, mitochondrial inhibitor	Q15119	769	0.9967	0.997	Enzyme	Kinase
[Pyruvate dehydrogenase (acetyl-transferring)] kinase isozyme 3, mitochondrial inhibitor	Q15120	28	0.9168	0.915	Enzyme	Kinase
[Pyruvate dehydrogenase (acetyl-transferring)] kinase isozyme 4, mitochondrial inhibitor	Q16654	39	0.9712	0.972	Enzyme	Kinase
1,25-dihydroxyvitamin D(3) 24-hydroxylase, mitochondrial inhibitor	Q07973	63	0.9993	0.999	Enzyme	Cytochrome P450
10 kDa heat shock protein, mitochondrial inhibitor	P61604	49	0.9583	0.958	Unclassified protein	NA
11-beta-hydroxysteroid dehydrogenase 1 inhibitor	P28845	2295	0.9926	0.993	Enzyme	Oxidoreductase
11-beta-hydroxysteroid dehydrogenase type 2 inhibitor	P80365	102	0.9934	0.993	Enzyme	NA
14-3-3 protein sigma inhibitor	P31947	6	0.9987	0.999	Unclassified protein	NA
15-hydroxyprostaglandin dehydrogenase [NAD(+)] inhibitor	P15428	3044	0.901	0.901	Enzyme	NA
17-beta-hydroxysteroid dehydrogenase 14 inhibitor	Q9BPX1	47	1.000	1.000	Enzyme	Oxidoreductase
17-beta-hydroxysteroid dehydrogenase type 1 inhibitor	P14061	373	0.9938	0.994	Enzyme	Oxidoreductase
17-beta-hydroxysteroid dehydrogenase type 2 inhibitor	P37059	470	0.9957	0.995	Enzyme	Oxidoreductase
17-beta-hydroxysteroid dehydrogenase type 3 inhibitor	P37058	176	0.998	0.998	Enzyme	Oxidoreductase
1-acyl-sn-glycerol-3-phosphate acyltransferase beta inhibitor	O15120	102	1.000	1.000	Enzyme	Transferase
1-phosphatidylinositol 4,5-bisphosphate phosphodiesterase gamma-2 inhibitor	P16885	12	0.9134	0.916	Enzyme	Hydrolase
2-5A-dependent ribonuclease activator	Q05823	3	1.000	1.000	Enzyme	Hydrolase
2-5A-dependent ribonuclease inhibitor	Q05823	8	1.000	1.000	Enzyme	Hydrolase
25-hydroxyvitamin D-1 alpha hydroxylase, mitochondrial inhibitor	O15528	10	0.9967	0.997	Enzyme	Cytochrome P450
26S proteasome non-ATPase regulatory subunit 14 inhibitor	O00487	91	0.9246	0.925	Enzyme	Protease
2-acylglycerol O-acyltransferase 2 inhibitor	Q3SYC2	176	0.9999	1.000	Enzyme	Transferase
2-amino-3-carboxymuconate-6-semialdehyde decarboxylase inhibitor	Q8TDX5	37	1.000	1.000	Enzyme	Lyase
2'-deoxynucleoside 5'-phosphate N-hydrolase 1 inhibitor	O43598	20	1.000	1.000	Enzyme	Hydrolase
2-Hydroxyacid oxidase 1 inhibitor	Q9UJM8	15	0.9984	0.998	Enzyme	Oxidoreductase
2-oxoglutarate receptor 1 antagonist	Q96P68	3	1.000	1.000	Membrane receptor	Family A G protein-coupled receptor
3 beta-hydroxysteroid dehydrogenase/Delta 5-->4-isomerase type 1 inhibitor	P14060	14	1.000	1.000	Enzyme	NA
3-beta-hydroxysteroid-Delta(8),Delta(7)-isomerase inhibitor	Q15125	7	0.992	0.992	Enzyme	Isomerase
3-hydroxy-3-methylglutaryl-coenzyme A reductase inhibitor	P04035	191	0.9979	0.998	Enzyme	Oxidoreductase
3-hydroxyacyl-CoA dehydrogenase type-2 inhibitor	Q99714	2234	0.878	0.877	Enzyme	Oxidoreductase

3-keto-steroid reductase inhibitor	P56937	3	1.000	1.000	Enzyme	Oxidoreductase
3-keto-steroid reductase/17-beta-hydroxysteroid dehydrogenase 7 inhibitor	P56937	4	1.000	1.000	Enzyme	Oxidoreductase
3-oxo-5-alpha-steroid 4-dehydrogenase 1 inhibitor	P18405	365	0.9965	0.996	Enzyme	Oxidoreductase
3-oxo-5-alpha-steroid 4-dehydrogenase 2 inhibitor	P31213	371	0.9973	0.997	Enzyme	Oxidoreductase
3-phosphoinositide-dependent protein kinase 1 inhibitor	O15530	615	0.9905	0.989	Enzyme	Kinase
40S ribosomal protein S27 inhibitor	P42677	7	1.000	1.000	Unclassified protein	NA
4-galactosyl-N-acetylglucosaminide 3-alpha-L-fucosyltransferase FUT6 inhibitor	P51993	5	1.000	1.000	Enzyme	Transferase
4-hydroxyphenylpyruvate dioxygenase inhibitor	P32754	220	0.9999	1.000	Enzyme	Oxidoreductase
5'-AMP-activated protein kinase catalytic subunit alpha-1 inhibitor	Q13131	36	0.9293	0.931	Enzyme	Kinase
5'-AMP-activated protein kinase subunit beta-1 inhibitor	Q9Y478	16	0.9013	0.89	Enzyme	Kinase
5-hydroxytryptamine receptor 1A agonist	P08908	334	0.9927	0.992	Membrane receptor	Family A G protein-coupled receptor
5-hydroxytryptamine receptor 1A antagonist	P08908	1717	0.9837	0.984	Membrane receptor	Family A G protein-coupled receptor
5-hydroxytryptamine receptor 1B agonist	P28222	56	0.9824	0.983	Membrane receptor	Family A G protein-coupled receptor
5-hydroxytryptamine receptor 1B antagonist	P28222	698	0.9868	0.987	Membrane receptor	Family A G protein-coupled receptor
5-hydroxytryptamine receptor 1D agonist	P28221	88	0.9992	0.999	Membrane receptor	Family A G protein-coupled receptor
5-hydroxytryptamine receptor 1D antagonist	P28221	830	0.9877	0.988	Membrane receptor	Family A G protein-coupled receptor
5-hydroxytryptamine receptor 1E antagonist	P28566	53	0.9261	0.928	Membrane receptor	Family A G protein-coupled receptor
5-hydroxytryptamine receptor 1F agonist	P30939	3	1.000	1.000	Membrane receptor	Family A G protein-coupled receptor
5-hydroxytryptamine receptor 1F antagonist	P30939	122	0.9919	0.992	Membrane receptor	Family A G protein-coupled receptor
5-hydroxytryptamine receptor 2A agonist	P28223	304	0.99	0.99	Membrane receptor	Family A G protein-coupled receptor
5-hydroxytryptamine receptor 2A antagonist	P28223	2073	0.9841	0.984	Membrane receptor	Family A G protein-coupled receptor
5-hydroxytryptamine receptor 2B agonist	P41595	293	0.9946	0.995	Membrane receptor	Family A G protein-coupled receptor
5-hydroxytryptamine receptor 2B antagonist	P41595	766	0.9557	0.956	Membrane receptor	Family A G protein-coupled receptor
5-hydroxytryptamine receptor 2C agonist	P28335	596	0.9957	0.995	Membrane receptor	Family A G protein-coupled receptor
5-hydroxytryptamine receptor 2C antagonist	P28335	1241	0.982	0.982	Membrane receptor	Family A G protein-coupled receptor

5-hydroxytryptamine receptor 3A agonist	P46098	24	0.9793	0.98	Ion channel	Ligand-gated ion channel
5-hydroxytryptamine receptor 3A antagonist	P46098	489	0.9862	0.986	Ion channel	Ligand-gated ion channel
5-hydroxytryptamine receptor 3D antagonist	Q70Z44	186	0.9848	0.985	Ion channel	Ligand-gated ion channel
5-hydroxytryptamine receptor 4 agonist	Q13639	243	0.9975	0.996	Membrane receptor	Family A G protein-coupled receptor
5-hydroxytryptamine receptor 4 antagonist	Q13639	189	0.9832	0.984	Membrane receptor	Family A G protein-coupled receptor
5-hydroxytryptamine receptor 5A antagonist	P47898	188	0.9781	0.978	Membrane receptor	Family A G protein-coupled receptor
5-hydroxytryptamine receptor 6 agonist	P50406	123	0.9996	1.000	Membrane receptor	Family A G protein-coupled receptor
5-hydroxytryptamine receptor 6 antagonist	P50406	1741	0.9914	0.992	Membrane receptor	Family A G protein-coupled receptor
5-hydroxytryptamine receptor 7 agonist	P34969	14	0.9833	0.984	Membrane receptor	Family A G protein-coupled receptor
5-hydroxytryptamine receptor 7 antagonist	P34969	645	0.9799	0.98	Membrane receptor	Family A G protein-coupled receptor
5'-nucleotidase inhibitor	P21589	90	0.9997	1.000	Enzyme	Phosphatase
6-phosphofructo-2-kinase/fructose-2,6-bisphosphatase 1 inhibitor	P16118	17	1.000	1.000	Enzyme	NA
6-phosphofructo-2-kinase/fructose-2,6-bisphosphatase 2 inhibitor	O60825	20	1.000	1.000	Enzyme	NA
6-phosphofructo-2-kinase/fructose-2,6-bisphosphatase 3 inhibitor	Q16875	281	0.9992	0.999	Enzyme	NA
6-phosphofructo-2-kinase/fructose-2,6-bisphosphatase 4 inhibitor	Q16877	32	1.000	1.000	Enzyme	NA
6-phosphogluconate dehydrogenase, decarboxylating inhibitor	P52209	11	1.000	1.000	Enzyme	Oxidoreductase
72 kDa type IV collagenase inhibitor	P08253	2855	0.9835	0.983	Enzyme	Protease
7-alpha-hydroxycholest-4-en-3-one 12-alpha-hydroxylase inhibitor	Q9UNU6	88	0.9916	0.992	Enzyme	Oxidoreductase
7-dehydrocholesterol reductase inhibitor	Q9UBM7	17	0.9977	0.998	Enzyme	Oxidoreductase
A disintegrin and metalloproteinase with thrombospondin motifs 1 inhibitor	Q9UHI8	4	0.9971	0.997	Enzyme	Protease
A disintegrin and metalloproteinase with thrombospondin motifs 4 inhibitor	O75173	239	0.9864	0.987	Enzyme	Protease
A disintegrin and metalloproteinase with thrombospondin motifs 5 inhibitor	Q9UNA0	397	0.9963	0.996	Enzyme	Protease
Acetylcholine receptor subunit alpha antagonist	P02708	10	0.9157	0.774	Ion channel	Ligand-gated ion channel
Acetylcholine receptor subunit beta antagonist	P11230	4	1.000	1.000	Ion channel	Ligand-gated ion channel
Acetylcholine receptor subunit epsilon antagonist	Q04844	14	0.9278	0.928	Ion channel	Ligand-gated ion channel
Acetylcholine receptor subunit gamma antagonist	P07510	66	0.9975	0.998	Ion channel	Ligand-gated ion

							channel
Acetylcholinesterase activator	P22303	8	0.9997	1.000	Enzyme	Hydrolase
Acetylcholinesterase inhibitor	P22303	3083	0.9828	0.983	Enzyme	Hydrolase
Acetyl-CoA carboxylase 1 inhibitor	Q13085	379	0.9961	0.996	Enzyme	NA
Acetyl-CoA carboxylase 2 inhibitor	O00763	882	0.9983	0.998	Enzyme	Ligase
Acetyl-coenzyme A synthetase, cytoplasmic inhibitor	Q9NR19	319	1.000	1.000	Enzyme	Ligase
Acetyl-coenzyme A transporter 1 inhibitor	O00400	130	1.000	1.000	Transporter	Electrochemical transporter
Acid ceramidase inhibitor	Q13510	119	1.000	1.000	Enzyme	Hydrolase
Acidic mammalian chitinase inhibitor	Q9BZP6	110	0.9865	0.977	Enzyme	NA
Acid-sensing ion channel 3 blocker	Q9UHC3	30	0.9954	0.996	Ion channel	Ligand-gated ion channel
Acrosin inhibitor	P10323	60	0.9999	1.000	Enzyme	Protease
Activated CDC42 kinase 1 inhibitor	Q07912	498	0.9924	0.992	Enzyme	Kinase
Activin receptor type-1 antagonist	Q04771	139	0.9826	0.982	Enzyme	Kinase
Activin receptor type-1B antagonist	P36896	48	0.9348	0.935	Enzyme	Kinase
Acyl-CoA (8-3)-desaturase inhibitor	O60427	39	0.9736	0.974	Enzyme	NA
Acyl-protein thioesterase 1 inhibitor	O75608	8	0.8876	0.89	Enzyme	Hydrolase
Acyl-protein thioesterase 2 inhibitor	O95372	7	0.9916	0.992	Enzyme	Hydrolase
Adenomatous polyposis coli protein inhibitor	P25054	19	0.9665	0.967	Other cytosolic protein	NA
Adenosine deaminase inhibitor	P00813	46	0.9965	0.997	Enzyme	Hydrolase
Adenosine kinase inhibitor	P55263	405	0.9989	0.999	Enzyme	NA
Adenosine receptor A1 agonist	P30542	220	0.9935	0.994	Membrane receptor	Family A G protein-coupled receptor
Adenosine receptor A1 antagonist	P30542	1169	0.9862	0.986	Membrane receptor	Family A G protein-coupled receptor
Adenosine receptor A2a agonist	P29274	266	0.9984	0.998	Membrane receptor	Family A G protein-coupled receptor
Adenosine receptor A2a antagonist	P29274	1568	0.9885	0.989	Membrane receptor	Family A G protein-coupled receptor
Adenosine receptor A2b agonist	P29275	197	0.9966	0.997	Membrane receptor	Family A G protein-coupled receptor
Adenosine receptor A2b antagonist	P29275	1146	0.9917	0.992	Membrane receptor	Family A G protein-coupled receptor
Adenosine receptor A3 agonist	P0DMS8	172	0.9924	0.992	Membrane receptor	Family A G protein-coupled receptor
Adenosine receptor A3 antagonist	P0DMS8	1193	0.9842	0.984	Membrane receptor	Family A G protein-

						coupled receptor
Adenosylhomocysteinase inhibitor	P23526	133	0.9998	1.000	Enzyme	Hydrolase
Adenylate cyclase type 1 inhibitor	Q08828	92	0.9953	0.995	Enzyme	NA
Adenylate cyclase type 5 inhibitor	O95622	24	0.9996	1.000	Enzyme	Lyase
Adenylate cyclase type 8 inhibitor	P40145	17	0.9999	1.000	Enzyme	Lyase
Adenylosuccinate synthetase isozyme 2 inhibitor	P30520	3	1.000	1.000	Enzyme	Ligase
ADP-ribosyl cyclase/cyclic ADP-ribose hydrolase 1 inhibitor	P28907	143	0.997	0.996	Enzyme	NA
Advanced glycosylation end product-specific receptor antagonist	Q15109	24	0.9984	0.998	Unclassified protein	NA
Alcohol dehydrogenase 1A inhibitor	P07327	16	0.9997	0.98	Enzyme	Oxidoreductase
Alcohol dehydrogenase 1C inhibitor	P00326	6	1.000	1.000	Enzyme	Oxidoreductase
Alcohol dehydrogenase class-3 inhibitor	P11766	64	0.9999	1.000	Enzyme	Oxidoreductase
Aldehyde dehydrogenase 1A1 inhibitor	P00352	12507	0.8857	0.886	Enzyme	Oxidoreductase
Aldehyde dehydrogenase family 1 member A3 inhibitor	P47895	38	0.9864	0.987	Enzyme	Oxidoreductase
Aldehyde dehydrogenase X, mitochondrial inhibitor	P30837	7	0.9857	0.986	Enzyme	Oxidoreductase
Aldehyde dehydrogenase, dimeric NADP-preferring inhibitor	P30838	37	0.9806	0.977	Enzyme	Oxidoreductase
Aldehyde dehydrogenase, mitochondrial inhibitor	P05091	74	0.9736	0.974	Enzyme	Oxidoreductase
Aldehyde oxidase inhibitor	Q06278	14	0.9398	0.933	Enzyme	Oxidoreductase
Aldo-keto reductase family 1 member A1 inhibitor	P14550	27	0.9982	0.998	Enzyme	Oxidoreductase
Aldo-keto reductase family 1 member B1 inhibitor	P15121	715	0.9964	0.996	Enzyme	Oxidoreductase
Aldo-keto reductase family 1 member B10 inhibitor	O60218	99	0.9936	0.994	Enzyme	Oxidoreductase
Aldo-keto reductase family 1 member C1 inhibitor	Q04828	83	0.9881	0.988	Enzyme	Oxidoreductase
Aldo-keto reductase family 1 member C2 inhibitor	P52895	134	0.9917	0.992	Enzyme	Oxidoreductase
Aldo-keto reductase family 1 member C3 inhibitor	P42330	527	0.9911	0.991	Enzyme	NA
Aldo-keto reductase family 1 member C4 inhibitor	P17516	14	0.9729	0.973	Enzyme	Oxidoreductase
ALK tyrosine kinase receptor antagonist	Q9UM73	1724	0.9896	0.99	Enzyme	Kinase
Alkaline phosphatase, germ cell type inhibitor	P10696	77	0.9345	0.932	Enzyme	Phosphatase
Alkaline phosphatase, tissue-nonspecific isozyme inhibitor	P05186	182	0.9836	0.984	Enzyme	NA
All-trans-retinol dehydrogenase [NAD(+)] ADH1B inhibitor	P00325	7	1.000	1.000	Enzyme	Oxidoreductase
Alpha-(1,3)-fucosyltransferase 7 inhibitor	Q11130	5	0.9998	1.000	Enzyme	Transferase
Alpha-1,3-mannosyl-glycoprotein 2-beta-N-acetylglucosaminyltransferase inhibitor	P26572	6	1.000	1.000	Enzyme	Transferase
Alpha-1,6-mannosyl-glycoprotein 2-beta-N-acetylglucosaminyltransferase inhibitor	Q10469	196	0.9966	0.996	Enzyme	Transferase
Alpha-1A adrenergic receptor agonist	P35348	193	0.9955	0.996	Membrane receptor	Family A G protein-coupled receptor

Alpha-1A adrenergic receptor antagonist	P35348	884	0.982	0.982	Membrane receptor	Family A G protein-coupled receptor
Alpha-1B adrenergic receptor agonist	P35368	46	0.9766	0.977	Membrane receptor	Family A G protein-coupled receptor
Alpha-1B adrenergic receptor antagonist	P35368	1030	0.9845	0.984	Membrane receptor	Family A G protein-coupled receptor
Alpha-1D adrenergic receptor agonist	P25100	44	0.9992	0.999	Membrane receptor	Family A G protein-coupled receptor
Alpha-1D adrenergic receptor antagonist	P25100	603	0.9896	0.99	Membrane receptor	Family A G protein-coupled receptor
Alpha-2A adrenergic receptor agonist	P08913	94	0.9998	1.000	Membrane receptor	Family A G protein-coupled receptor
Alpha-2A adrenergic receptor antagonist	P08913	502	0.9747	0.974	Membrane receptor	Family A G protein-coupled receptor
Alpha-2B adrenergic receptor agonist	P18089	61	0.9991	0.999	Membrane receptor	Family A G protein-coupled receptor
Alpha-2B adrenergic receptor antagonist	P18089	237	0.9679	0.968	Membrane receptor	Family A G protein-coupled receptor
Alpha-2C adrenergic receptor agonist	P18825	111	0.9989	0.999	Membrane receptor	Family A G protein-coupled receptor
Alpha-2C adrenergic receptor antagonist	P18825	381	0.9788	0.979	Membrane receptor	Family A G protein-coupled receptor
Alpha-amylase 1A inhibitor	P0DUB6	5	0.9988	0.999	Enzyme	Hydrolase
Alpha-crystallin B chain inhibitor	P02511	6	1.000	1.000	Unclassified protein	NA
Alpha-enolase inhibitor	P06733	5	0.8748	0.866	Enzyme	NA
Alpha-galactosidase A inhibitor	P06280	359	0.8163	0.814	Enzyme	Hydrolase
Alpha-ketoglutarate-dependent dioxygenase alkB homolog 3 inhibitor	Q96Q83	38	0.9995	1.000	Enzyme	Oxidoreductase
Alpha-ketoglutarate-dependent dioxygenase FTO inhibitor	Q9C0B1	105	0.9992	0.999	Enzyme	Oxidoreductase
Alpha-mannosidase 2 inhibitor	Q16706	4	1.000	1.000	Enzyme	Hydrolase
Alpha-synuclein inhibitor	P37840	140	0.9943	0.994	Unclassified protein	NA
Amiloride-sensitive amine oxidase [copper-containing] inhibitor	P19801	30	0.999	0.999	Enzyme	NA
Amiloride-sensitive sodium channel subunit alpha blocker	P37088	37	1.000	1.000	Ion channel	Ligand-gated ion channel
Amine oxidase [flavin-containing] A inhibitor	P21397	1329	0.9834	0.983	Enzyme	Oxidoreductase
Amine oxidase [flavin-containing] B inhibitor	P27338	2519	0.9833	0.983	Enzyme	Oxidoreductase
Aminoacyl tRNA synthase complex-interacting multifunctional protein 2 inhibitor	Q13155	4	0.9997	1.000	Unclassified protein	NA
Aminopeptidase B inhibitor	Q9H4A4	7	0.9995	1.000	Enzyme	Protease
Aminopeptidase N inhibitor	P15144	236	0.9898	0.99	Enzyme	Protease

AMP deaminase 1 inhibitor	P23109	3	1.000	1.000	Enzyme	Hydrolase
AMP deaminase 2 inhibitor	Q01433	21	1.000	1.000	Enzyme	NA
AMP deaminase 3 inhibitor	Q01432	44	0.9999	1.000	Enzyme	Hydrolase
Amphiregulin inhibitor	P15514	5	1.000	1.000	Secreted protein	NA
Amyloid-beta precursor protein inhibitor	P05067	575	0.9801	0.98	Membrane receptor	NA
Androgen receptor agonist	P10275	352	0.9984	0.999	Transcription factor	Nuclear receptor
Androgen receptor antagonist	P10275	1364	0.9937	0.993	Transcription factor	Nuclear receptor
Angiopoietin-1 receptor antagonist	Q02763	774	0.9851	0.984	Enzyme	Kinase
Angiotensin-converting enzyme 2 inhibitor	Q9BYF1	107	0.9994	0.999	Enzyme	Protease
Angiotensin-converting enzyme inhibitor	P12821	464	0.9973	0.997	Enzyme	Protease
Anoctamin-1 inhibitor	Q5XXA6	51	0.9889	0.989	Ion channel	Other ion channel
Anoctamin-2 inhibitor	Q9NQ90	7	0.9177	0.919	Ion channel	Other ion channel
Anthrax toxin receptor 2 antagonist	P58335	6	0.9979	0.998	Unclassified protein	NA
AP2-associated protein kinase 1 inhibitor	Q2M2I8	72	0.9783	0.979	Enzyme	Kinase
Apelin receptor agonist	P35414	183	0.9926	0.993	Membrane receptor	Family A G protein-coupled receptor
Apelin receptor antagonist	P35414	13	0.9337	0.934	Membrane receptor	Family A G protein-coupled receptor
Apolipoprotein B-100 inhibitor	P04114	42	1.000	1.000	Secreted protein	NA
Apoptosis regulator Bcl-2 inhibitor	P10415	1172	0.9906	0.99	Ion channel	Other ion channel
Appetite-regulating hormone inhibitor	Q9UBU3	40	1.000	1.000	Secreted protein	NA
Arachidonate 5-lipoxygenase-activating protein inhibitor	P20292	1911	0.9988	0.999	Other cytosolic protein	NA
Arginase-1 inhibitor	P05089	95	1.000	1.000	Enzyme	Hydrolase
Arginase-2, mitochondrial inhibitor	P78540	41	1.000	1.000	Enzyme	Hydrolase
Aromatase inhibitor	P11511	1558	0.9931	0.993	Enzyme	Cytochrome P450
Arsenite methyltransferase inhibitor	Q9HBK9	8	1.000	1.000	Enzyme	Transferase
Aryl hydrocarbon receptor agonist	P35869	15	0.9905	0.983	Transcription factor	NA
Aryl hydrocarbon receptor antagonist	P35869	9	0.9962	0.996	Transcription factor	NA
Arylamine N-acetyltransferase 1 inhibitor	P18440	26	0.9999	1.000	Enzyme	Transferase
Asparagine synthetase [glutamine-hydrolyzing] inhibitor	P08243	3	1.000	1.000	Enzyme	NA
Aspartyl aminopeptidase inhibitor	Q9ULA0	4	1.000	1.000	Enzyme	Protease
ATPase family AAA domain-containing protein 2 inhibitor	Q6PL18	28	0.9777	0.978	Epigenetic regulator	Reader
ATP-binding cassette sub-family C member 8 inhibitor	Q09428	19	0.9734	0.975	Transporter	Primary active transporter

ATP-binding cassette sub-family C member 9 inhibitor	O60706	14	0.9798	0.981	Transporter	Primary active transporter
ATP-citrate synthase inhibitor	P53396	44	0.9952	0.996	Enzyme	Transferase
ATP-dependent Clp protease proteolytic subunit, mitochondrial inhibitor	Q16740	3	0.8476	0.852	Enzyme	Hydrolase
ATP-dependent DNA helicase Q1 inhibitor	P46063	1431	0.8322	0.831	Enzyme	NA
ATP-dependent RNA helicase DDX3X inhibitor	O00571	26	0.9723	0.973	Enzyme	NA
ATP-dependent translocase ABCB1 inhibitor	P08183	967	0.9758	0.975	Transporter	Primary active transporter
ATP-sensitive inward rectifier potassium channel 1 blocker	P48048	441	0.9958	0.996	Ion channel	Voltage-gated ion channel
ATP-sensitive inward rectifier potassium channel 11 blocker	Q14654	93	0.9999	1.000	Ion channel	Voltage-gated ion channel
Atrial natriuretic peptide receptor 1 agonist	P16066	40	0.9961	0.996	Enzyme	Lyase
Atrial natriuretic peptide receptor 3 antagonist	P17342	4	1.000	1.000	Membrane receptor	NA
Atypical chemokine receptor 3 agonist	P25106	485	0.9971	0.997	Membrane receptor	Family A G protein-coupled receptor
Atypical chemokine receptor 3 antagonist	P25106	11	0.8401	0.844	Membrane receptor	Family A G protein-coupled receptor
Atypical kinase COQ8B, mitochondrial inhibitor	Q96D53	3	0.983	0.983	Enzyme	Kinase
Aurora kinase A inhibitor	O14965	2229	0.9801	0.98	Enzyme	Kinase
Aurora kinase B inhibitor	Q96GD4	1545	0.9797	0.98	Enzyme	Kinase
Aurora kinase C inhibitor	Q9UQB9	47	0.9219	0.915	Enzyme	Kinase
B1 bradykinin receptor antagonist	P46663	660	0.998	0.998	Membrane receptor	Family A G protein-coupled receptor
B2 bradykinin receptor antagonist	P30411	332	0.9989	0.999	Membrane receptor	Family A G protein-coupled receptor
Baculoviral IAP repeat-containing protein 2 activator	Q13490	44	1.000	1.000	Enzyme	NA
Baculoviral IAP repeat-containing protein 2 inhibitor	Q13490	462	0.9998	1.000	Enzyme	NA
Baculoviral IAP repeat-containing protein 3 inhibitor	Q13489	102	0.9999	1.000	Enzyme	NA
B-cell CLL/lymphoma 9 protein inhibitor	O00512	11	0.9505	0.951	Unclassified protein	NA
B-cell lymphoma 3 protein inhibitor	P20749	8	1.000	1.000	Unclassified protein	NA
B-cell lymphoma 6 protein inhibitor	P41182	95	0.9906	0.99	Transcription factor	NA
B-cell receptor CD22 antagonist	P20273	21	1.000	1.000	Surface antigen; Adhesion; Membrane receptor	NA
Bcl-2 homologous antagonist/killer inhibitor	Q16611	25	0.9992	0.998	Unclassified protein	NA
Bcl2-associated agonist of cell death inhibitor	Q92934	48	0.9704	0.971	Other cytosolic protein	NA
Bcl-2-like protein 1 inhibitor	Q07817	804	0.9941	0.994	Ion channel	Other ion channel

Bcl-2-like protein 11 inhibitor	O43521	19	0.9994	0.999	Unclassified protein	NA
Bcl-2-like protein 2 inhibitor	Q92843	16	0.8823	0.879	Other cytosolic protein	NA
Bcl-2-related protein A1 inhibitor	Q16548	56	0.9841	0.985	Unclassified protein	NA
BCL-6 corepressor inhibitor	Q6W2J9	11	1.000	1.000	Unclassified protein	NA
BDNF/NT-3 growth factors receptor agonist	Q16620	16	0.9977	0.998	Enzyme	Kinase
BDNF/NT-3 growth factors receptor antagonist	Q16620	244	0.9684	0.96	Enzyme	Kinase
Beta-1 adrenergic receptor agonist	P08588	528	0.9981	0.998	Membrane receptor	Family A G protein-coupled receptor
Beta-1 adrenergic receptor antagonist	P08588	382	0.9872	0.987	Membrane receptor	Family A G protein-coupled receptor
Beta-1,4-mannosyl-glycoprotein 4-beta-N-acetylglucosaminyltransferase inhibitor	Q09327	14	1.000	1.000	Enzyme	Transferase
Beta-2 adrenergic receptor agonist	P07550	623	0.9988	0.999	Membrane receptor	Family A G protein-coupled receptor
Beta-2 adrenergic receptor antagonist	P07550	483	0.99	0.99	Membrane receptor	Family A G protein-coupled receptor
Beta-3 adrenergic receptor agonist	P13945	960	0.9991	0.999	Membrane receptor	Family A G protein-coupled receptor
Beta-3 adrenergic receptor antagonist	P13945	194	0.9844	0.984	Membrane receptor	Family A G protein-coupled receptor
Beta-adrenergic receptor kinase 1 antagonist	P25098	325	0.9731	0.972	Enzyme	Kinase
Beta-adrenergic receptor kinase 2 antagonist	P35626	33	0.9339	0.935	Enzyme	Kinase
Beta-galactosidase inhibitor	P16278	16	0.9996	1.000	Enzyme	Hydrolase
Beta-galactoside alpha-2,6-sialyltransferase 1 inhibitor	P15907	18	0.9915	0.989	Enzyme	Transferase
Beta-glucuronidase inhibitor	P08236	185	0.9677	0.968	Enzyme	Hydrolase
Beta-hexosaminidase subunit alpha inhibitor	P06865	20	0.9501	0.951	Enzyme	Hydrolase
Beta-hexosaminidase subunit beta inhibitor	P07686	21	0.9943	0.994	Enzyme	Hydrolase
Betaine--homocysteine S-methyltransferase 1 inhibitor	Q93088	14	1.000	1.000	Enzyme	Transferase
Beta-secretase 1 inhibitor	P56817	6537	0.9868	0.987	Enzyme	Protease
Beta-secretase 2 inhibitor	Q9Y5Z0	1595	0.9964	0.997	Enzyme	Protease
Bifunctional 3'-5' exonuclease/ATP-dependent helicase WRN inhibitor	Q14191	7	1.000	1.000	Enzyme	NA
Bifunctional epoxide hydrolase 2 inhibitor	P34913	1650	0.9901	0.99	Enzyme	Protease
Bifunctional methylenetetrahydrofolate dehydrogenase/cyclohydrolase, mitochondrial inhibitor	P13995	37	0.9999	1.000	Enzyme	Oxidoreductase
Bifunctional phosphoribosylaminoimidazole carboxylase/phosphoribosylaminoimidazole succinocarboxamide synthetase inhibitor	P22234	38	1.000	1.000	Enzyme	NA
Bifunctional purine biosynthesis protein ATIC inhibitor	P31939	28	0.9694	0.97	Enzyme	NA

Bile acid receptor agonist	Q96RI1	446	0.9925	0.993	Transcription factor	Nuclear receptor
Bile acid receptor antagonist	Q96RI1	202	0.9726	0.973	Transcription factor	Nuclear receptor
Bile salt export pump inhibitor	O95342	153	0.8378	0.837	Transporter	Primary active transporter
Bile salt-activated lipase inhibitor	P19835	6	1.000	1.000	Enzyme	Hydrolase
Bis(5'-adenosyl)-triphosphatase inhibitor	P49789	8	1.000	1.000	Enzyme	Hydrolase
BMP-2-inducible protein kinase inhibitor	Q9NSY1	19	0.9527	0.955	Enzyme	Kinase
Bombesin receptor subtype-3 agonist	P32247	305	0.9987	0.999	Membrane receptor	Family A G protein-coupled receptor
Bombesin receptor subtype-3 antagonist	P32247	40	0.9991	0.999	Membrane receptor	Family A G protein-coupled receptor
Bone morphogenetic protein 1 inhibitor	P13497	757	0.9999	1.000	Enzyme	Protease
Bone morphogenetic protein 4 inhibitor	P12644	20	1.000	1.000	Enzyme	Protease
Bone morphogenetic protein receptor type-1A antagonist	P36894	26	0.8888	0.89	Enzyme	Kinase
Bone morphogenetic protein receptor type-1B antagonist	O00238	18	0.8879	0.89	Enzyme	Kinase
Bone morphogenetic protein receptor type-2 antagonist	Q13873	13	0.9179	0.918	Enzyme	Kinase
Branched-chain-amino-acid aminotransferase, cytosolic inhibitor	P54687	16	0.9999	1.000	Enzyme	Transferase
Branched-chain-amino-acid aminotransferase, mitochondrial inhibitor	O15382	117	0.9961	0.996	Enzyme	Transferase
Breakpoint cluster region protein inhibitor	P11274	378	0.9915	0.992	Enzyme	Kinase
Breast cancer type 1 susceptibility protein inhibitor	P38398	66	0.9417	0.925	Enzyme	NA
Broad substrate specificity ATP-binding cassette transporter ABCG2 inhibitor	Q9UNQ0	736	0.9896	0.99	Transporter	Primary active transporter
Bromodomain adjacent to zinc finger domain protein 2A inhibitor	Q9UIF9	33	0.9917	0.992	Epigenetic regulator	Reader
Bromodomain adjacent to zinc finger domain protein 2B inhibitor	Q9UIF8	23	0.9601	0.961	Epigenetic regulator	Reader
Bromodomain and PHD finger-containing protein 3 inhibitor	Q9ULD4	21	0.97	0.97	Epigenetic regulator	Reader
Bromodomain testis-specific protein inhibitor	Q58F21	61	0.9661	0.965	Epigenetic regulator	Reader
Bromodomain-containing protein 1 inhibitor	O95696	72	0.9979	0.998	Epigenetic regulator	Reader
Bromodomain-containing protein 2 inhibitor	P25440	268	0.9958	0.996	Epigenetic regulator	Reader
Bromodomain-containing protein 3 inhibitor	Q15059	271	0.9965	0.997	Epigenetic regulator	Reader
Bromodomain-containing protein 4 inhibitor	O60885	1975	0.9884	0.988	Epigenetic regulator	Reader
Bromodomain-containing protein 7 inhibitor	Q9NPI1	18	0.9999	1.000	Epigenetic regulator	Reader
Bromodomain-containing protein 9 inhibitor	Q9H8M2	141	0.9926	0.993	Epigenetic regulator	Reader
BTB/POZ domain-containing protein KCTD12 inhibitor	Q96CX2	17	1.000	1.000	Unclassified protein	NA
Butyrophilin subfamily 3 member A1 inhibitor	O00481	17	0.9996	1.000	Unclassified protein	NA

C-1-tetrahydrofolate synthase, cytoplasmic inhibitor	P11586	13	1.000	1.000	Enzyme	NA
C3a anaphylatoxin chemotactic receptor agonist	A8K2H7, Q16581	131	0.9924	0.992	Membrane receptor	Family A G protein-coupled receptor
C3a anaphylatoxin chemotactic receptor antagonist	A8K2H7, Q16581	108	0.9987	0.999	Membrane receptor	Family A G protein-coupled receptor
C5a anaphylatoxin chemotactic receptor 1 antagonist	P21730	326	0.9957	0.996	Membrane receptor	Family A G protein-coupled receptor
CAAX prenyl protease 2 inhibitor	Q9Y256	17	0.9991	0.999	Enzyme	Protease
CAD protein inhibitor	P27708	6	0.9911	0.991	Enzyme	NA
Calcitonin gene-related peptide 1 inhibitor	P06881	14	0.9828	0.981	Secreted protein	NA
Calcitonin gene-related peptide type 1 receptor antagonist	Q16602	478	0.9973	0.997	Membrane receptor	Family B G protein-coupled receptor
Calcitonin receptor agonist	P30988	63	1.000	1.000	Membrane receptor	Family B G protein-coupled receptor
Calcitonin receptor antagonist	P30988	12	1.000	1.000	Membrane receptor	Family B G protein-coupled receptor
Calcium release-activated calcium channel protein 1 blocker	Q96D31	18	0.9881	0.987	Ion channel	Other ion channel
Calcium/calmodulin-dependent protein kinase kinase 2 inhibitor	Q96RR4	75	0.9728	0.973	Enzyme	Kinase
Calcium/calmodulin-dependent protein kinase type 1D inhibitor	Q8IU85	24	0.9136	0.915	Enzyme	Kinase
Calcium/calmodulin-dependent protein kinase type II subunit alpha inhibitor	Q9UQM7	20	0.8624	0.868	Enzyme	Kinase
Calcium/calmodulin-dependent protein kinase type II subunit delta inhibitor	Q13557	141	0.9875	0.987	Enzyme	Kinase
Calcium/calmodulin-dependent protein kinase type II subunit gamma inhibitor	Q13555	56	0.9499	0.949	Enzyme	Kinase
Calcium-activated potassium channel subunit alpha-1 blocker	Q12791	45	0.9675	0.967	Ion channel	Voltage-gated ion channel
Calmodulin-1 inhibitor	P0DP23	6	0.9293	0.931	Unclassified protein	NA
Calpain small subunit 1 inhibitor	P04632	66	0.9999	1.000	Enzyme	Protease
Calpain-1 catalytic subunit inhibitor	P07384	580	0.9982	0.998	Enzyme	Protease
Calpain-2 catalytic subunit inhibitor	P17655	157	0.9999	1.000	Enzyme	Protease
Calpain-9 inhibitor	O14815	8	0.9998	1.000	Enzyme	Hydrolase
cAMP and cAMP-inhibited cGMP 3',5'-cyclic phosphodiesterase 10A inhibitor	Q9Y233	3653	0.9937	0.994	Enzyme	Phosphodiesterase
cAMP-dependent protein kinase catalytic subunit alpha inhibitor	P17612	95	0.9631	0.962	Enzyme	Kinase
cAMP-dependent protein kinase catalytic subunit gamma inhibitor	P22612	169	0.9815	0.981	Enzyme	Kinase
cAMP-specific 3',5'-cyclic phosphodiesterase 4A activator	P27815	13	0.9934	0.985	Enzyme	Phosphodiesterase
cAMP-specific 3',5'-cyclic phosphodiesterase 4A inhibitor	P27815	1765	0.991	0.991	Enzyme	Phosphodiesterase
cAMP-specific 3',5'-cyclic phosphodiesterase 4B inhibitor	Q07343	1366	0.9859	0.986	Enzyme	Phosphodiesterase
cAMP-specific 3',5'-cyclic phosphodiesterase 4C inhibitor	Q08493	108	0.9865	0.986	Enzyme	Phosphodiesterase

cAMP-specific 3',5'-cyclic phosphodiesterase 4D inhibitor	Q08499	787	0.9813	0.98	Enzyme	Phosphodiesterase
cAMP-specific 3',5'-cyclic phosphodiesterase 7B inhibitor	Q9NP56	44	0.968	0.969	Enzyme	Phosphodiesterase
Cannabinoid receptor 1 agonist	P21554	910	0.989	0.989	Membrane receptor	Family A G protein-coupled receptor
Cannabinoid receptor 1 antagonist	P21554	1708	0.9858	0.986	Membrane receptor	Family A G protein-coupled receptor
Cannabinoid receptor 2 agonist	P34972	2010	0.9922	0.992	Membrane receptor	Family A G protein-coupled receptor
Cannabinoid receptor 2 antagonist	P34972	1210	0.9918	0.992	Membrane receptor	Family A G protein-coupled receptor
Carbamoyl-phosphate synthase [ammonia], mitochondrial allosteric inhibitor	P31327	15	0.9583	0.959	Enzyme	Ligase
Carbamoyl-phosphate synthase [ammonia], mitochondrial inhibitor	P31327	15	0.9583	0.959	Enzyme	Ligase
Carbonic anhydrase 1 inhibitor	P00915	5137	0.9935	0.994	Enzyme	Lyase
Carbonic anhydrase 12 inhibitor	O43570	3217	0.9914	0.991	Enzyme	Lyase
Carbonic anhydrase 13 inhibitor	Q8N1Q1	90	0.9767	0.977	Enzyme	Lyase
Carbonic anhydrase 14 inhibitor	Q9ULX7	519	0.9892	0.989	Enzyme	Lyase
Carbonic anhydrase 2 inhibitor	P00918	6015	0.9935	0.994	Enzyme	Lyase
Carbonic anhydrase 3 inhibitor	P07451	87	0.9608	0.961	Enzyme	Lyase
Carbonic anhydrase 4 inhibitor	P22748	926	0.993	0.993	Enzyme	Lyase
Carbonic anhydrase 5A, mitochondrial inhibitor	P35218	343	0.9807	0.981	Enzyme	Lyase
Carbonic anhydrase 5B, mitochondrial inhibitor	Q9Y2D0	274	0.9917	0.992	Enzyme	Lyase
Carbonic anhydrase 6 inhibitor	P23280	171	0.9737	0.974	Enzyme	Lyase
Carbonic anhydrase 7 inhibitor	P43166	857	0.9916	0.992	Enzyme	Lyase
Carbonic anhydrase 9 inhibitor	Q16790	4225	0.992	0.992	Enzyme	Lyase
Carbonic anhydrase-related protein 11 inhibitor	O75493	26	0.9997	1.000	Enzyme	NA
Carbonyl reductase [NADPH] 1 inhibitor	P16152	17	0.9916	0.99	Enzyme	Oxidoreductase
Carboxypeptidase A1 inhibitor	P15085	48	0.9996	1.000	Enzyme	Protease
Carboxypeptidase A2 inhibitor	P48052	11	1.000	1.000	Enzyme	Protease
Carboxypeptidase A4 inhibitor	Q9UI42	11	1.000	1.000	Enzyme	Protease
Carboxypeptidase B inhibitor	P15086	63	0.9849	0.985	Enzyme	Protease
Carboxypeptidase B2 inhibitor	Q96IY4	198	0.9999	1.000	Enzyme	Protease
Carboxypeptidase N catalytic chain inhibitor	P15169	20	1.000	1.000	Enzyme	Protease
Carnitine O-palmitoyltransferase 1, liver isoform inhibitor	P50416	490	0.997	0.997	Enzyme	Transferase
Carnitine O-palmitoyltransferase 1, muscle isoform inhibitor	Q92523	166	0.9855	0.986	Transporter	Group translocator

Carnitine O-palmitoyltransferase 2, mitochondrial inhibitor	P23786	113	0.9959	0.996	Enzyme	Transferase
Casein kinase I isoform alpha inhibitor	P48729	83	0.962	0.962	Enzyme	Kinase
Casein kinase I isoform delta inhibitor	P48730	447	0.9853	0.985	Enzyme	Kinase
Casein kinase I isoform epsilon inhibitor	P49674	298	0.996	0.996	Enzyme	Kinase
Casein kinase I isoform gamma-1 inhibitor	Q9HCP0	12	0.8218	0.825	Enzyme	Kinase
Casein kinase I isoform gamma-2 inhibitor	P78368	30	0.929	0.929	Enzyme	Kinase
Casein kinase II subunit alpha 3 inhibitor	Q8NEV1	34	0.9675	0.968	Enzyme	Kinase
Casein kinase II subunit alpha inhibitor	P68400	696	0.9848	0.983	Enzyme	Kinase
Casein kinase II subunit alpha' inhibitor	P19784	160	0.9895	0.99	Enzyme	Kinase
Casein kinase II subunit beta inhibitor	P67870	349	0.9992	0.999	Enzyme	Kinase
Caspase-1 inhibitor	P29466	601	0.9896	0.989	Enzyme	Protease
Caspase-10 inhibitor	Q92851	8	0.9886	0.989	Enzyme	Protease
Caspase-2 inhibitor	P42575	33	0.999	0.999	Enzyme	Protease
Caspase-3 activator	P42574	6	0.9996	1.000	Enzyme	Protease
Caspase-3 inhibitor	P42574	962	0.9969	0.997	Enzyme	Protease
Caspase-4 inhibitor	P49662	24	0.9454	0.944	Enzyme	Protease
Caspase-5 inhibitor	P51878	20	0.9114	0.913	Enzyme	Protease
Caspase-6 inhibitor	P55212	193	0.9981	0.995	Enzyme	Protease
Caspase-7 inhibitor	P55210	420	0.9962	0.996	Enzyme	Protease
Caspase-8 inhibitor	Q14790	297	0.9961	0.996	Enzyme	Protease
Caspase-9 inhibitor	P55211	28	0.9893	0.99	Enzyme	Protease
Cat eye syndrome critical region protein 2 inhibitor	Q9BXF3	59	0.9729	0.967	Epigenetic regulator	Reader
Catechol O-methyltransferase inhibitor	P21964	114	0.9981	0.998	Enzyme	Transferase
Catenin beta-1 inhibitor	P35222	53	0.9629	0.96	Unclassified protein	NA
Cathepsin B inhibitor	P07858	942	0.9917	0.992	Enzyme	Protease
Cathepsin D inhibitor	P07339	1117	0.9911	0.991	Enzyme	Protease
Cathepsin E inhibitor	P14091	87	0.9719	0.972	Enzyme	Protease
Cathepsin F inhibitor	Q9UBX1	28	0.9942	0.994	Enzyme	Protease
Cathepsin G inhibitor	P08311	157	0.9764	0.977	Enzyme	Protease
Cathepsin K inhibitor	P43235	1647	0.9955	0.995	Enzyme	Protease
Cathepsin L2 inhibitor	O60911	131	0.9966	0.997	Enzyme	Protease
Cathepsin S inhibitor	P25774	1930	0.9968	0.997	Enzyme	Protease

Cathepsin Z inhibitor	Q9UBR2	5	0.8002	0.801	Enzyme	Protease
C-C chemokine receptor type 1 antagonist	P32246	483	0.9965	0.997	Membrane receptor	Family A G protein-coupled receptor
C-C chemokine receptor type 10 antagonist	P46092	33	1.000	1.000	Membrane receptor	Family A G protein-coupled receptor
C-C chemokine receptor type 2 antagonist	P41597	1041	0.9964	0.996	Membrane receptor	Family A G protein-coupled receptor
C-C chemokine receptor type 3 antagonist	P51677	846	0.9951	0.995	Membrane receptor	Family A G protein-coupled receptor
C-C chemokine receptor type 4 antagonist	P51679	326	0.9916	0.992	Membrane receptor	Family A G protein-coupled receptor
C-C chemokine receptor type 5 agonist	P51681	8	0.9139	0.915	Membrane receptor	Family A G protein-coupled receptor
C-C chemokine receptor type 5 antagonist	P51681	1451	0.9919	0.992	Membrane receptor	Family A G protein-coupled receptor
C-C chemokine receptor type 6 agonist	P51684	25	1.000	1.000	Membrane receptor	Family A G protein-coupled receptor
C-C chemokine receptor type 6 antagonist	P51684	11	0.9098	0.91	Membrane receptor	Family A G protein-coupled receptor
C-C chemokine receptor type 8 antagonist	P51685	148	0.9966	0.997	Membrane receptor	Family A G protein-coupled receptor
C-C chemokine receptor type 9 antagonist	P51686	302	0.9975	0.998	Membrane receptor	Family A G protein-coupled receptor
C-C chemokine receptor-like 2 antagonist	O00421	150	1.000	1.000	Membrane receptor	Family A G protein-coupled receptor
C-C motif chemokine 2 inhibitor	P13500	3	0.9678	0.809	Membrane receptor	Family A G protein-coupled receptor
C-C motif chemokine 5 inhibitor	P13501	27	1.000	1.000	Membrane receptor	Family A G protein-coupled receptor
CCR4-NOT transcription complex subunit 7 inhibitor	Q9UIV1	10	1.000	1.000	Enzyme	Hydrolase
CD40 ligand inhibitor	P29965	6	0.9997	1.000	Secreted protein	NA
CD63 antigen inhibitor	P08962	11	1.000	1.000	Surface antigen	NA
CDK-activating kinase assembly factor MAT1 inhibitor	P51948	69	0.9168	0.92	Unclassified protein	NA
Cell division control protein 42 homolog inhibitor	P60953	10	0.8351	0.837	Enzyme	Hydrolase
Cell division cycle 7-related protein kinase inhibitor	O00311	406	0.9988	0.999	Enzyme	Kinase
Cellular tumor antigen p53 inhibitor	P04637	764	0.9923	0.992	Transcription factor	NA
Centromere-associated protein E inhibitor	Q02224	22	1.000	1.000	Unclassified protein	NA
Ceramide glucosyltransferase inhibitor	Q16739	91	0.9999	1.000	Enzyme	Transferase
cGMP-dependent 3',5'-cyclic phosphodiesterase inhibitor	O00408	708	0.9915	0.99	Enzyme	Phosphodiesterase

cGMP-dependent protein kinase 2 inhibitor	Q13237	6	0.8759	0.879	Enzyme	Kinase
cGMP-inhibited 3',5'-cyclic phosphodiesterase 3A inhibitor	Q14432	240	0.9695	0.969	Enzyme	Phosphodiesterase
cGMP-inhibited 3',5'-cyclic phosphodiesterase 3B inhibitor	Q13370	112	0.9859	0.986	Enzyme	Phosphodiesterase
cGMP-specific 3',5'-cyclic phosphodiesterase inhibitor	O76074	1709	0.9893	0.989	Enzyme	Phosphodiesterase
Chemerin-like receptor 1 agonist	Q99788	14	1.000	1.000	Membrane receptor	Family A G protein-coupled receptor
Chemokine-like receptor 1 antagonist	Q99788	22	1.000	1.000	Membrane receptor	Family A G protein-coupled receptor
Chitotriosidase-1 inhibitor	Q13231	99	0.9997	1.000	Enzyme	NA
Cholecystokinin receptor type A agonist	P32238	164	0.9998	1.000	Membrane receptor	Family A G protein-coupled receptor
Cholecystokinin receptor type A antagonist	P32238	143	0.9839	0.984	Membrane receptor	Family A G protein-coupled receptor
Cholesteryl ester transfer protein inhibitor	P11597	768	0.9982	0.998	Ion channel	Other ion channel
Choline kinase alpha inhibitor	P35790	73	0.9955	0.996	Enzyme	Transferase
Choline O-acetyltransferase inhibitor	P28329	20	0.942	0.942	Enzyme	Transferase
Choline/ethanolaminephosphotransferase 1 inhibitor	Q9Y6K0	39	1.000	1.000	Enzyme	Transferase
Cholinesterase inhibitor	P06276	1612	0.989	0.989	Enzyme	Hydrolase
Chromobox protein homolog 7 inhibitor	O95931	8	0.9501	0.951	Epigenetic regulator	Reader
Chymase inhibitor	P23946	433	0.9979	0.998	Enzyme	Protease
Chymotrypsin-C inhibitor	Q99895	73	0.9511	0.952	Enzyme	Protease
Chymotrypsin-like elastase family member 1 inhibitor	Q9UNI1	17	0.9897	0.99	Enzyme	Protease
Chymotrypsin-like protease CTRL-1 inhibitor	P40313	11	1.000	1.000	Enzyme	Protease
Chymotrypsinogen B inhibitor	P17538	72	0.9694	0.97	Enzyme	Protease
Citron Rho-interacting kinase inhibitor	O14578	6	0.8323	0.836	Enzyme	Kinase
Clathrin heavy chain 1 inhibitor	Q00610	3	1.000	1.000	Structural protein	NA
CMP-N-acetylneuraminate-beta-galactosamide-alpha-2,3-sialyltransferase 1 inhibitor	Q11201	4	0.9324	0.93	Enzyme	Transferase
Coagulation factor IX inhibitor	P00740	393	0.995	0.995	Enzyme	Protease
Coagulation factor V inhibitor	P12259	7	0.8854	0.888	Secreted protein	NA
Coagulation factor VII inhibitor	P08709	491	0.9936	0.994	Enzyme	Protease
Coagulation factor X inhibitor	P00742	5178	0.9944	0.994	Enzyme	Protease
Coagulation factor XI inhibitor	P03951	1000	0.9977	0.998	Enzyme	Protease
Coagulation factor XII inhibitor	P00748	123	0.985	0.985	Enzyme	Protease
Coagulation factor XIII A chain inhibitor	P00488	95	0.9964	0.997	Enzyme	Aminoacyltransferase

Cocaine esterase inhibitor	O00748	131	0.9881	0.988	Enzyme	Hydrolase
Coiled-coil domain-containing protein 6 inhibitor	Q16204	13	0.8685	0.87	Unclassified protein	NA
Collagenase 3 inhibitor	P45452	2412	0.9902	0.99	Enzyme	Protease
Complement C1r subcomponent inhibitor	P00736	47	0.993	0.994	Enzyme	Protease
Complement C1s subcomponent inhibitor	P09871	105	0.9875	0.988	Enzyme	Protease
Complement C3 inhibitor	P01024	26	1.000	1.000	Secreted protein	NA
Complement component C9 inhibitor	P02748	3	1.000	1.000	Unclassified protein	NA
Complement factor B inhibitor	P00751	39	0.9804	0.98	Enzyme	Protease
Complement factor D inhibitor	P00746	897	0.9974	0.997	Enzyme	Protease
Cone cGMP-specific 3',5'-cyclic phosphodiesterase subunit alpha' inhibitor	P51160	35	0.9463	0.935	Enzyme	Phosphodiesterase
COP9 signalosome complex subunit 5 inhibitor	Q92905	12	0.9218	0.923	Enzyme	Hydrolase
Core-binding factor subunit beta inhibitor	Q13951	32	1.000	1.000	Unclassified protein	NA
Corticosteroid-binding globulin inhibitor	P08185	29	0.9999	1.000	Secreted protein	NA
Corticotropin-releasing factor receptor 1 agonist	P34998	21	1.000	1.000	Membrane receptor	Family B G protein-coupled receptor
Corticotropin-releasing factor receptor 1 antagonist	P34998	1207	0.9968	0.997	Membrane receptor	Family B G protein-coupled receptor
Corticotropin-releasing factor receptor 2 agonist	Q13324	26	1.000	1.000	Membrane receptor	Family B G protein-coupled receptor
Corticotropin-releasing factor receptor 2 antagonist	Q13324	18	0.9935	0.994	Membrane receptor	Family B G protein-coupled receptor
CREB-binding protein inhibitor	Q92793	297	0.9752	0.975	Epigenetic regulator	Reader; Writer
C-terminal-binding protein 2 inhibitor	P56545	13	1.000	1.000	Unclassified protein	NA
Cullin-4A inhibitor	Q13619	24	0.9824	0.984	Unclassified protein	NA
CX3C chemokine receptor 1 antagonist	P49238	9	1.000	1.000	Membrane receptor	Family A G protein-coupled receptor
C-X-C chemokine receptor type 1 antagonist	P25024	174	0.9814	0.982	Membrane receptor	Family A G protein-coupled receptor
C-X-C chemokine receptor type 2 antagonist	P25025	576	0.9951	0.995	Membrane receptor	Family A G protein-coupled receptor
C-X-C chemokine receptor type 3 agonist	P49682	253	0.9987	0.999	Membrane receptor	Family A G protein-coupled receptor
C-X-C chemokine receptor type 3 antagonist	P49682	637	0.9997	1.000	Membrane receptor	Family A G protein-coupled receptor
C-X-C chemokine receptor type 4 agonist	P61073	115	0.9992	0.999	Membrane receptor	Family A G protein-coupled receptor
C-X-C chemokine receptor type 4 antagonist	P61073	397	0.9937	0.994	Membrane receptor	Family A G protein-coupled receptor

C-X-C chemokine receptor type 5 antagonist	P32302	39	1.000	1.000	Membrane receptor	Family A G protein-coupled receptor
C-X-C chemokine receptor type 6 antagonist	O00574	41	1.000	1.000	Membrane receptor	Family A G protein-coupled receptor
C-X-C motif chemokine 10 inhibitor	P02778	165	1.000	1.000	Secreted protein	NA
Cyclic AMP-dependent transcription factor ATF-1 inhibitor	P18846	17	1.000	1.000	Transcription factor	NA
Cyclic GMP-AMP synthase inhibitor	Q8N884	120	0.9924	0.993	Enzyme	Transferase
Cyclin-A1 inhibitor	P78396	53	0.9782	0.979	Other cytosolic protein	NA
Cyclin-A2 inhibitor	P20248	1433	0.9838	0.984	Other cytosolic protein	NA
Cyclin-C inhibitor	P24863	245	0.9755	0.975	Other cytosolic protein	NA
Cyclin-dependent kinase 1 inhibitor	P06493	1727	0.9817	0.982	Enzyme	Kinase
Cyclin-dependent kinase 10 inhibitor	Q15131	5	0.9351	0.936	Enzyme	NA
Cyclin-dependent kinase 12 inhibitor	Q9NYV4	27	0.9884	0.988	Enzyme	Kinase
Cyclin-dependent kinase 13 inhibitor	Q14004	3	0.9128	0.915	Enzyme	NA
Cyclin-dependent kinase 14 inhibitor	O94921	64	0.958	0.959	Enzyme	Kinase
Cyclin-dependent kinase 18 inhibitor	Q07002	5	0.9094	0.912	Enzyme	Kinase
Cyclin-dependent kinase 19 inhibitor	Q9BWU1	60	0.9926	0.993	Enzyme	Kinase
Cyclin-dependent kinase 2 inhibitor	P24941	1533	0.9816	0.981	Enzyme	Kinase
Cyclin-dependent kinase 2-associated protein 1 inhibitor	O14519	17	1.000	1.000	Unclassified protein	NA
Cyclin-dependent kinase 3 inhibitor	Q00526	7	0.8666	0.829	Enzyme	Kinase
Cyclin-dependent kinase 4 inhibitor	P11802	1659	0.9854	0.985	Enzyme	Kinase
Cyclin-dependent kinase 5 activator 1 inhibitor	Q15078	4	1.000	1.000	Enzyme	Kinase
Cyclin-dependent kinase 5 inhibitor	Q00535	137	0.9765	0.977	Enzyme	Kinase
Cyclin-dependent kinase 6 inhibitor	Q00534	316	0.9916	0.991	Enzyme	Kinase
Cyclin-dependent kinase 7 inhibitor	P50613	120	0.9786	0.978	Enzyme	Kinase
Cyclin-dependent kinase 8 inhibitor	P49336	294	0.9915	0.992	Enzyme	Kinase
Cyclin-dependent kinase 9 inhibitor	P50750	723	0.9794	0.979	Enzyme	Kinase
Cyclin-dependent kinase inhibitor 1 inhibitor	P38936	7	0.8446	0.847	Other cytosolic protein	NA
Cyclin-dependent kinase-like 1 inhibitor	Q00532	4	0.8498	0.853	Enzyme	Kinase
Cyclin-dependent kinases regulatory subunit 1 inhibitor	P61024	22	1.000	1.000	Unclassified protein	NA
Cyclin-G-associated kinase inhibitor	O14976	56	0.9681	0.968	Enzyme	Kinase
Cyclin-H inhibitor	P51946	147	0.9788	0.979	Other cytosolic protein	NA
Cyclin-K inhibitor	O75909	63	0.9645	0.965	Enzyme	Kinase

Cyclin-T1 inhibitor	O60563	311	0.9986	0.999	Other cytosolic protein	NA
Cystathionine beta-synthase inhibitor	P35520	10	0.9945	0.995	Enzyme	NA
Cystathionine gamma-lyase inhibitor	P32929	28	0.9845	0.985	Enzyme	Lyase
Cysteine protease ATG4B inhibitor	Q9Y4P1	177	0.9123	0.911	Enzyme	NA
Cysteinyl leukotriene receptor 1 antagonist	Q9Y271	477	0.9973	0.997	Membrane receptor	Family A G protein-coupled receptor
Cysteinyl leukotriene receptor 2 antagonist	Q9NS75	71	0.9809	0.98	Membrane receptor	Family A G protein-coupled receptor
Cystic fibrosis transmembrane conductance regulator inhibitor	P13569	61	0.9938	0.994	Ion channel	Other ion channel
Cystine/glutamate transporter inhibitor	Q9UPY5	15	0.9537	0.955	Transporter	Electrochemical transporter
Cytidine deaminase inhibitor	P32320	12	1.000	1.000	Enzyme	Hydrolase
Cytochrome b-245 heavy chain inhibitor	P04839	6	1.000	1.000	Transporter	Transmembrane 1-electron transfer carriers
Cytochrome b-c1 complex subunit 7 inhibitor	P14927	5	1.000	1.000	Transporter	Transmembrane 1-electron transfer carriers
Cytochrome c oxidase subunit 2 inhibitor	P00403	8	0.955	0.956	Transporter	Primary active transporter
Cytochrome P450 11B1, mitochondrial inhibitor	P15538	919	0.9983	0.998	Enzyme	Cytochrome P450
Cytochrome P450 11B2, mitochondrial inhibitor	P19099	1228	0.9976	0.998	Enzyme	Cytochrome P450
Cytochrome P450 1A1 inhibitor	P04798	285	0.9884	0.988	Enzyme	Cytochrome P450
Cytochrome P450 1A2 inhibitor	P05177	1040	0.9344	0.934	Enzyme	Cytochrome P450
Cytochrome P450 1B1 inhibitor	Q16678	300	0.9941	0.994	Enzyme	Cytochrome P450
Cytochrome P450 26A1 inhibitor	O43174	136	0.9998	1.000	Enzyme	Cytochrome P450
Cytochrome P450 26B1 inhibitor	Q9NR63	14	0.9208	0.922	Enzyme	Cytochrome P450
Cytochrome P450 2A13 inhibitor	Q16696	5	0.9595	0.962	Enzyme	Cytochrome P450
Cytochrome P450 2A6 inhibitor	P11509	177	0.9979	0.998	Enzyme	Cytochrome P450
Cytochrome P450 2B6 activator	P20813	3	0.9901	0.99	Enzyme	Cytochrome P450
Cytochrome P450 2B6 inhibitor	P20813	104	0.9408	0.942	Enzyme	Cytochrome P450
Cytochrome P450 2C18 inhibitor	P33260	12	1.000	1.000	Enzyme	Cytochrome P450
Cytochrome P450 2C19 inhibitor	P33261	3228	0.8894	0.888	Enzyme	Cytochrome P450
Cytochrome P450 2C8 inhibitor	P10632	206	0.8699	0.866	Enzyme	Cytochrome P450
Cytochrome P450 2C9 inhibitor	P11712	3615	0.8839	0.883	Enzyme	Cytochrome P450
Cytochrome P450 2D6 inhibitor	P10635	3516	0.9069	0.906	Enzyme	Cytochrome P450

Cytochrome P450 2E1 inhibitor	P05181	42	0.9557	0.957	Enzyme	Cytochrome P450
Cytochrome P450 2J2 inhibitor	P51589	36	0.9125	0.915	Enzyme	NA
Cytochrome P450 3A4 inhibitor	P08684	7098	0.9003	0.9	Enzyme	Cytochrome P450
Cytochrome P450 3A7 inhibitor	P24462	12	0.9294	0.933	Enzyme	Oxidoreductase
Cytochrome P450 4Z1 inhibitor	Q86W10	3	1.000	1.000	Enzyme	Oxidoreductase
Cytoplasmic tyrosine-protein kinase BMX inhibitor	P51813	123	0.9666	0.965	Enzyme	Kinase
Cytosol aminopeptidase inhibitor	P28838	32	0.9993	0.999	Enzyme	Protease
Cytosolic carboxypeptidase 2 inhibitor	Q5U5Z8	4	0.9991	0.999	Enzyme	Protease
Cytosolic phospholipase A2 beta inhibitor	P0C869	31	0.9999	1.000	Enzyme	Hydrolase
Cytosolic phospholipase A2 gamma inhibitor	Q9UP65	3	0.9832	0.984	Enzyme	Hydrolase
Cytosolic phospholipase A2 inhibitor	P47712	238	0.9988	0.999	Enzyme	NA
D(1A) dopamine receptor agonist	P21728	237	0.993	0.993	Membrane receptor	Family A G protein-coupled receptor
D(1A) dopamine receptor antagonist	P21728	359	0.978	0.978	Membrane receptor	Family A G protein-coupled receptor
D(1B) dopamine receptor agonist	P21918	6	0.9992	0.999	Membrane receptor	Family A G protein-coupled receptor
D(1B) dopamine receptor antagonist	P21918	111	0.9812	0.981	Membrane receptor	Family A G protein-coupled receptor
D(2) dopamine receptor agonist	P14416	473	0.9936	0.994	Membrane receptor	Family A G protein-coupled receptor
D(2) dopamine receptor antagonist	P14416	3226	0.9875	0.987	Membrane receptor	Family A G protein-coupled receptor
D(3) dopamine receptor agonist	P35462	312	0.9967	0.997	Membrane receptor	Family A G protein-coupled receptor
D(3) dopamine receptor antagonist	P35462	2277	0.9899	0.99	Membrane receptor	Family A G protein-coupled receptor
D(4) dopamine receptor agonist	P21917	118	0.9881	0.988	Membrane receptor	Family A G protein-coupled receptor
D(4) dopamine receptor antagonist	P21917	1126	0.9898	0.99	Membrane receptor	Family A G protein-coupled receptor
D-3-phosphoglycerate dehydrogenase inhibitor	O43175	271	0.997	0.997	Enzyme	Oxidoreductase
D-amino-acid oxidase inhibitor	P14920	332	0.9994	0.999	Enzyme	Oxidoreductase
D-aspartate oxidase inhibitor	Q99489	3	0.9834	0.944	Enzyme	Oxidoreductase
DCN1-like protein 1 inhibitor	Q96GG9	123	0.9977	0.998	Unclassified protein	NA
dCTP pyrophosphatase 1 inhibitor	Q9H773	203	0.9974	0.997	Enzyme	Hydrolase
Death-associated protein kinase 1 inhibitor	P53355	35	0.9457	0.945	Enzyme	Kinase

Death-associated protein kinase 2 inhibitor	Q9UIK4	18	0.9048	0.91	Enzyme	Kinase
Death-associated protein kinase 3 inhibitor	O43293	44	0.9162	0.911	Enzyme	Kinase
Dedicator of cytokinesis protein 2 inhibitor	Q92608	5	1.000	1.000	Unclassified protein	NA
Delta(24)-sterol reductase inhibitor	Q15392	12	1.000	1.000	Enzyme	Oxidoreductase
Delta-type opioid receptor agonist	P41143	618	0.9893	0.989	Membrane receptor	Family A G protein-coupled receptor
Delta-type opioid receptor antagonist	P41143	1530	0.9889	0.989	Membrane receptor	Family A G protein-coupled receptor
Deoxycytidine kinase inhibitor	P27707	96	1.000	1.000	Enzyme	Transferase
Deoxyhypusine hydroxylase inhibitor	Q9BU89	5	0.9556	0.958	Enzyme	Oxidoreductase
Deoxyhypusine synthase inhibitor	P49366	8	0.9886	0.989	Enzyme	Transferase
Deoxyuridine 5'-triphosphate nucleotidohydrolase, mitochondrial inhibitor	P33316	173	1.000	1.000	Enzyme	NA
DEP domain-containing mTOR-interacting protein inhibitor	Q8TB45	7	1.000	1.000	Unclassified protein	NA
DGAT1 protein inhibitor	Q9BRH5	15	0.999	0.999	Enzyme	Transferase
Diacylglycerol kinase alpha inhibitor	P23743	12	0.9774	0.813	Enzyme	Transferase
Diacylglycerol kinase zeta inhibitor	Q13574	7	1.000	1.000	Enzyme	Transferase
Diacylglycerol lipase-alpha inhibitor	Q9Y4D2	135	0.9992	0.999	Enzyme	Hydrolase
Diacylglycerol lipase-beta inhibitor	Q8NCG7	8	0.9969	0.997	Enzyme	NA
Diacylglycerol O-acyltransferase 1 inhibitor	Q9BRH5, O75907	1118	0.9988	0.999	Enzyme	Transferase
Diacylglycerol O-acyltransferase 2 inhibitor	Q96PD7	49	0.9873	0.987	Enzyme	Transferase
Dickkopf-related protein 1 inhibitor	O94907	31	0.9975	0.998	Secreted protein	NA
Dihydrofolate reductase inhibitor	P00374	888	0.9964	0.996	Enzyme	Oxidoreductase
Dihydroorotate dehydrogenase (quinone), mitochondrial inhibitor	Q02127	708	0.9964	0.996	Enzyme	Oxidoreductase
Dihydropteridine reductase inhibitor	P09417	11	1.000	1.000	Enzyme	Oxidoreductase
Dihydropyrimidine dehydrogenase [NADP(+)] inhibitor	Q12882	11	1.000	1.000	Enzyme	NA
Dihydroxyacetone phosphate acyltransferase inhibitor	O15228	3	1.000	1.000	Enzyme	Transferase
Dipeptidase 1 inhibitor	P16444	10	1.000	1.000	Enzyme	Protease
Dipeptidyl peptidase 1 inhibitor	P53634	659	0.9985	0.998	Enzyme	Protease
Dipeptidyl peptidase 2 inhibitor	Q9UHL4	517	0.9988	0.999	Enzyme	Protease
Dipeptidyl peptidase 4 inhibitor	P27487	3394	0.9948	0.995	Enzyme	Protease
Dipeptidyl peptidase 8 inhibitor	Q6V1X1	574	0.9986	0.999	Enzyme	Protease
Dipeptidyl peptidase 9 inhibitor	Q86TI2	401	0.9965	0.997	Enzyme	Protease
Diphosphomevalonate decarboxylase inhibitor	P53602	5	1.000	1.000	Enzyme	Lyase

Disabled homolog 2-interacting protein inhibitor	Q5VWQ8	6	0.9583	0.959	Unclassified protein	NA
Discoidin domain-containing receptor 2 antagonist	Q16832	193	0.9726	0.973	Enzyme	Kinase
Disintegrin and metalloproteinase domain-containing protein 10 inhibitor	O14672	133	0.9968	0.997	Enzyme	Protease
Disintegrin and metalloproteinase domain-containing protein 12 inhibitor	O43184	5	0.9736	0.974	Enzyme	Protease
Disintegrin and metalloproteinase domain-containing protein 17 inhibitor	P78536	1641	0.9971	0.997	Enzyme	Protease
Disintegrin and metalloproteinase domain-containing protein 33 inhibitor	Q9BZ11	12	1.000	1.000	Enzyme	Protease
Disintegrin and metalloproteinase domain-containing protein 8 inhibitor	P78325	27	0.9822	0.982	Enzyme	Protease
Disintegrin and metalloproteinase domain-containing protein 9 inhibitor	Q13443	41	0.9868	0.986	Enzyme	Protease
Disks large homolog 4 inhibitor	P78352	33	1.000	1.000	Unclassified protein	NA
DNA (cytosine-5)-methyltransferase 1 inhibitor	P26358	111	0.9888	0.989	Epigenetic regulator	Writer
DNA (cytosine-5)-methyltransferase 3A inhibitor	Q9Y6K1	29	0.9542	0.954	Epigenetic regulator	Reader; Writer
DNA (cytosine-5)-methyltransferase 3B inhibitor	Q9UBC3	23	0.9726	0.973	Epigenetic regulator	Reader; Writer
DNA (cytosine-5)-methyltransferase 3-like inhibitor	Q9UJW3	4	0.9674	0.97	Epigenetic regulator	Writer
DNA dC->dU-editing enzyme APOBEC-3A inhibitor	P31941	209	0.9302	0.929	Enzyme	Hydrolase
DNA dC->dU-editing enzyme APOBEC-3G inhibitor	Q9HC16	469	0.9512	0.952	Enzyme	Hydrolase
DNA ligase 1 inhibitor	P18858	10	0.8219	0.826	Enzyme	Ligase
DNA nucleotidylexotransferase inhibitor	P04053	5	1.000	1.000	Enzyme	Transferase
DNA polymerase alpha catalytic subunit inhibitor	P09884	33	0.9724	0.973	Enzyme	Transferase
DNA polymerase beta inhibitor	P06746	5111	0.8611	0.861	Enzyme	NA
DNA polymerase eta inhibitor	Q9Y253	4	0.9976	0.998	Enzyme	Transferase
DNA polymerase kappa inhibitor	Q9UBT6	6	0.841	0.83	Enzyme	NA
DNA polymerase lambda inhibitor	Q9UGP5	19	0.9992	0.999	Enzyme	NA
DNA polymerase subunit gamma-1 inhibitor	P54098	5	0.9276	0.935	Enzyme	Transferase
DNA repair endonuclease XPF inhibitor	Q92889	55	0.9992	0.999	Enzyme	Hydrolase
DNA repair protein complementing XP-A cells inhibitor	P23025	12	1.000	1.000	Other nuclear protein	NA
DNA repair protein complementing XP-G cells inhibitor	P28715	46	1.000	1.000	Other nuclear protein	NA
DNA repair protein RAD51 homolog 1 inhibitor	Q06609	26	0.9898	0.99	Unclassified protein	NA
DNA repair protein RAD52 homolog inhibitor	P43351	33	0.9593	0.958	Other nuclear protein	NA
DNA topoisomerase 1 inhibitor	P11387	173	0.9914	0.992	Enzyme	Isomerase
DNA topoisomerase 2-alpha inhibitor	P11388	145	0.9775	0.978	Enzyme	Isomerase
DNA topoisomerase 2-beta inhibitor	Q02880	108	0.9791	0.979	Enzyme	Isomerase
DNA-(apurinic or apyrimidinic site) endonuclease inhibitor	P27695	102	0.9588	0.957	Enzyme	NA

DNA-3-methyladenine glycosylase inhibitor	P29372	4	0.9757	0.978	Enzyme	NA
DNA-dependent protein kinase catalytic subunit inhibitor	P78527	926	0.9892	0.989	Enzyme	Kinase
DNA-directed RNA polymerase I subunit RPA1 activator	O95602	19	1.000	1.000	Enzyme	Transferase
Dolichyl-diphosphooligosaccharide--protein glycosyltransferase 48 kDa subunit inhibitor	P39656	12	1.000	1.000	Enzyme	NA
Dopamine beta-hydroxylase inhibitor	P09172	13	1.000	1.000	Enzyme	Oxidoreductase
Dual 3',5'-cyclic-AMP and -GMP phosphodiesterase 11A inhibitor	Q9HCR9	161	0.9792	0.979	Enzyme	Phosphodiesterase
Dual specificity calcium/calmodulin-dependent 3',5'-cyclic nucleotide phosphodiesterase 1A inhibitor	P54750	24	0.8991	0.889	Enzyme	Phosphodiesterase
Dual specificity calcium/calmodulin-dependent 3',5'-cyclic nucleotide phosphodiesterase 1B inhibitor	Q01064	64	0.955	0.956	Enzyme	Phosphodiesterase
Dual specificity calcium/calmodulin-dependent 3',5'-cyclic nucleotide phosphodiesterase 1C inhibitor	Q14123	56	0.9967	0.997	Enzyme	Phosphodiesterase
Dual specificity mitogen-activated protein kinase kinase 1 inhibitor	Q02750	887	0.9801	0.98	Enzyme	Kinase
Dual specificity mitogen-activated protein kinase kinase 2 inhibitor	P36507	91	0.951	0.935	Enzyme	Kinase
Dual specificity mitogen-activated protein kinase kinase 3 inhibitor	P46734	11	0.8884	0.887	Enzyme	Kinase
Dual specificity mitogen-activated protein kinase kinase 4 inhibitor	P45985	9	0.811	0.814	Enzyme	Kinase
Dual specificity mitogen-activated protein kinase kinase 5 inhibitor	Q13163	10	0.8092	0.807	Enzyme	Kinase
Dual specificity mitogen-activated protein kinase kinase 7 inhibitor	O14733	141	0.9758	0.976	Enzyme	Kinase
Dual specificity protein kinase CLK1 inhibitor	P49759	288	0.9624	0.962	Enzyme	Kinase
Dual specificity protein kinase CLK2 inhibitor	P49760	107	0.9546	0.955	Enzyme	Kinase
Dual specificity protein kinase CLK3 inhibitor	P49761	47	0.9305	0.931	Enzyme	Kinase
Dual specificity protein kinase CLK4 inhibitor	Q9HAZ1	341	0.9856	0.986	Enzyme	Kinase
Dual specificity protein kinase TTK inhibitor	P33981	1034	0.9973	0.997	Enzyme	Kinase
Dual specificity protein phosphatase 1 inhibitor	P28562	11	1.000	1.000	Enzyme	Phosphatase
Dual specificity protein phosphatase 22 inhibitor	Q9NRW4	17	0.9999	1.000	Enzyme	Phosphatase
Dual specificity protein phosphatase 23 inhibitor	Q9BVJ7	3	0.9977	0.998	Enzyme	Phosphatase
Dual specificity protein phosphatase 3 inhibitor	P51452	179	0.9443	0.945	Enzyme	Phosphatase
Dual specificity protein phosphatase CDC14A inhibitor	Q9UNH5	3	0.9999	1.000	Enzyme	NA
Dual specificity testis-specific protein kinase 2 inhibitor	Q96S53	6	1.000	1.000	Enzyme	Kinase
Dual specificity tyrosine-phosphorylation-regulated kinase 1A inhibitor	Q13627	674	0.9797	0.98	Enzyme	Kinase
Dual specificity tyrosine-phosphorylation-regulated kinase 1B inhibitor	Q9Y463	289	0.9833	0.983	Enzyme	Kinase
Dual specificity tyrosine-phosphorylation-regulated kinase 2 inhibitor	Q92630	168	0.9757	0.976	Enzyme	Kinase
Dual specificity tyrosine-phosphorylation-regulated kinase 3 inhibitor	O43781	65	0.9589	0.938	Enzyme	Kinase
Dynamin-1 inhibitor	Q05193	18	0.9999	1.000	Enzyme; Structural protein	NA

Dynamin-1-like protein inhibitor	O00429	41	1.000	1.000	Enzyme	Hydrolase
Dynamin-2 inhibitor	P50570	12	1.000	1.000	Enzyme	NA
E3 ubiquitin-protein ligase Mdm2 inhibitor	Q00987	1703	0.9961	0.996	Other nuclear protein	NA
E3 ubiquitin-protein ligase SMURF1 inhibitor	Q9HCE7	78	1.000	1.000	Enzyme	Transferase
E3 ubiquitin-protein ligase SMURF2 inhibitor	Q9HAU4	26	1.000	1.000	Enzyme	Transferase
E3 ubiquitin-protein ligase TRIM33 inhibitor	Q9UPN9	34	0.9616	0.961	Epigenetic regulator	Reader
E3 ubiquitin-protein ligase XIAP inhibitor	P98170	713	0.9997	1.000	Other cytosolic protein	NA
Early activation antigen CD69 inhibitor	Q07108	7	0.9992	0.999	Membrane receptor	NA
Ectonucleotide pyrophosphatase/phosphodiesterase family member 1 inhibitor	P22413	129	0.9945	0.994	Enzyme	NA
Ectonucleotide pyrophosphatase/phosphodiesterase family member 2 inhibitor	Q13822	701	0.9824	0.982	Enzyme	NA
Ectonucleotide pyrophosphatase/phosphodiesterase family member 3 inhibitor	O14638	46	0.9996	1.000	Enzyme	Hydrolase
EF-hand calcium-binding domain-containing protein 4B inhibitor	Q9BSW2	56	1.000	1.000	Unclassified protein	NA
Egl nine homolog 1 inhibitor	Q9GZT9	447	0.999	0.999	Enzyme	Oxidoreductase
eIF-2-alpha kinase GCN2 inhibitor	Q9P2K8	27	0.9969	0.997	Enzyme	Kinase
ELAV-like protein 1 inhibitor	Q15717	16	0.9989	0.999	Unclassified protein	NA
ELAV-like protein 3 inhibitor	Q14576	6	0.9999	1.000	Unclassified protein	NA
Elongation of very long chain fatty acids protein 3 inhibitor	Q9HB03	9	0.8776	0.879	Enzyme	Transferase
Elongation of very long chain fatty acids protein 6 inhibitor	Q9H5J4	98	1.000	1.000	Enzyme	Transferase
Elongin-B inhibitor	Q15370	5	1.000	1.000	Unclassified protein	NA
Emopamil-binding protein-like inhibitor	Q9BY08	4	0.9343	0.937	Unclassified protein	NA
Endoplasmic reticulum aminopeptidase 1 inhibitor	Q9NZ08	61	0.9879	0.985	Enzyme	Protease
Endoplasmic reticulum aminopeptidase 2 inhibitor	Q6P179	81	0.9974	0.997	Enzyme	Protease
Endoplasmic reticulum chaperone BiP inhibitor	P11021	3	0.8946	0.898	Unclassified protein	NA
Endoplasmic reticulum mannosyl-oligosaccharide 1,2-alpha-mannosidase inhibitor	Q9UKM7	11	1.000	1.000	Enzyme	Hydrolase
Endoplasmin inhibitor	P14625	22	0.9325	0.937	Other membrane protein	NA
Endothelial lipase inhibitor	Q9Y5X9	210	0.9942	0.994	Enzyme	Hydrolase
Endothelial PAS domain-containing protein 1 inhibitor	Q99814	277	0.9836	0.984	Transcription factor	NA
Endothelin receptor type B antagonist	P24530	742	0.9984	0.999	Membrane receptor	Family A G protein-coupled receptor
Endothelin-1 receptor antagonist	P25101	1149	0.9988	0.999	Membrane receptor	Family A G protein-coupled receptor
Endothelin-converting enzyme 1 inhibitor	P42892	275	0.9939	0.994	Enzyme	Protease
Enteropeptidase inhibitor	P98073	226	1.000	1.000	Enzyme	Protease

Eosinophil peroxidase inhibitor	P11678	3	0.997	0.953	Enzyme	Oxidoreductase
Ephrin type-A receptor 1 antagonist	P21709	10	0.8316	0.842	Enzyme	Kinase
Ephrin type-A receptor 2 agonist	P29317	4	1.000	1.000	Enzyme	Kinase
Ephrin type-A receptor 2 antagonist	P29317	405	0.9785	0.978	Enzyme	Kinase
Ephrin type-A receptor 3 antagonist	P29320	21	0.8616	0.863	Enzyme	Kinase
Ephrin type-A receptor 4 antagonist	P54764	36	0.9087	0.911	Enzyme	Kinase
Ephrin type-B receptor 1 antagonist	P54762	7	0.8654	0.79	Enzyme	Kinase
Ephrin type-B receptor 2 antagonist	P29323	37	0.9149	0.905	Enzyme	Kinase
Ephrin type-B receptor 3 antagonist	P54753	27	0.921	0.918	Enzyme	Kinase
Ephrin type-B receptor 4 antagonist	P54760	756	0.9877	0.987	Enzyme	Kinase
Epidermal growth factor receptor antagonist	P00533	6496	0.9753	0.975	Enzyme	Kinase
Epithelial discoidin domain-containing receptor 1 antagonist	Q08345	180	0.9732	0.973	Enzyme	Kinase
Epoxide hydrolase 1 inhibitor	P07099	103	0.9952	0.995	Enzyme	Protease
Equilibrative nucleoside transporter 1 inhibitor	Q99808	176	0.9985	0.999	Transporter	Electrochemical transporter
Erythropoietin receptor agonist	P19235	10	1.000	1.000	Membrane receptor	NA
Erythropoietin receptor antagonist	P19235	8	0.9761	0.976	Membrane receptor	NA
E-selectin inhibitor	P16581	100	0.9993	0.999	Adhesion	NA
Estrogen receptor agonist	P03372	284	0.9979	0.998	Transcription factor	Nuclear receptor
Estrogen receptor antagonist	P03372	1369	0.9891	0.989	Transcription factor	Nuclear receptor
Estrogen receptor beta agonist	Q92731	263	0.9976	0.998	Transcription factor	Nuclear receptor
Estrogen receptor beta antagonist	Q92731	1321	0.9947	0.995	Transcription factor	Nuclear receptor
Estrogen-related receptor gamma agonist	P62508	51	0.9999	1.000	Transcription factor	Nuclear receptor
Estrogen-related receptor gamma antagonist	P62508	55	0.9863	0.986	Transcription factor	Nuclear receptor
Eukaryotic elongation factor 2 kinase inhibitor	O00418	39	0.9768	0.948	Enzyme	Kinase
Eukaryotic initiation factor 4A-I inhibitor	P60842	29	0.9961	0.996	Enzyme	Hydrolase
Eukaryotic initiation factor 4A-III inhibitor	P38919	39	1.000	1.000	Enzyme	Hydrolase
Eukaryotic translation initiation factor 2-alpha kinase 1 inhibitor	Q9BQI3	34	0.992	0.992	Enzyme	Kinase
Eukaryotic translation initiation factor 2-alpha kinase 3 inhibitor	Q9NZJ5	109	0.999	0.999	Enzyme	Kinase
Eukaryotic translation initiation factor 4E inhibitor	P06730	121	1.000	1.000	Other nuclear protein	NA
Eukaryotic translation initiation factor 4E-binding protein 1 inhibitor	Q13541	7	0.9999	1.000	Other nuclear protein	NA
Excitatory amino acid transporter 1 inhibitor	P43003	65	0.9999	1.000	Transporter	Electrochemical transporter

Excitatory amino acid transporter 2 inhibitor	P43004	63	0.9997	1.000	Transporter	Electrochemical transporter
Excitatory amino acid transporter 3 inhibitor	P43005	64	0.9999	1.000	Transporter	Electrochemical transporter
Exportin-1 inhibitor	O14980	34	0.9967	0.997	Unclassified protein	NA
Extracellular calcium-sensing receptor agonist	P41180	60	0.9999	1.000	Membrane receptor	Family C G protein-coupled receptor
Extracellular calcium-sensing receptor antagonist	P41180	296	0.9999	1.000	Membrane receptor	Family C G protein-coupled receptor
Eyes absent homolog 2 inhibitor	O00167	5	0.9993	0.999	Enzyme	NA
Eyes absent homolog 3 inhibitor	Q99504	4	1.000	1.000	Enzyme	Hydrolase
Farnesyl pyrophosphate synthase allosteric inhibitor	P14324	13	0.9927	0.993	Enzyme	Transferase
Farnesyl pyrophosphate synthase inhibitor	P14324	239	0.9986	0.999	Enzyme	Transferase
Fascin inhibitor	Q16658	41	0.9938	0.994	Unclassified protein	NA
Fatty acid synthase inhibitor	P49327	184	0.9845	0.984	Enzyme	Transferase
Fatty acid-binding protein 5 inhibitor	Q01469	8	0.9921	0.992	Auxiliary transport protein	Fatty acid binding protein family
Fatty acid-binding protein, adipocyte inhibitor	P15090	62	0.9984	0.998	Auxiliary transport protein	Fatty acid binding protein family
Fatty acid-binding protein, heart inhibitor	P05413	22	0.9874	0.987	Auxiliary transport protein	Fatty acid binding protein family
Fatty-acid amide hydrolase 1 inhibitor	O00519	1073	0.9925	0.993	Enzyme	Hydrolase
Fibroblast growth factor 2 inhibitor	P09038	5	0.8921	0.894	Secreted protein	NA
Fibroblast growth factor receptor 1 agonist	P11362	5	1.000	1.000	Enzyme	Kinase
Fibroblast growth factor receptor 1 antagonist	P11362	2215	0.9878	0.988	Enzyme	Kinase
Fibroblast growth factor receptor 2 antagonist	P21802	970	0.9864	0.986	Enzyme	Kinase
Fibroblast growth factor receptor 3 antagonist	P22607	1294	0.9918	0.992	Enzyme	Kinase
Fibroblast growth factor receptor 4 antagonist	P22455	918	0.9885	0.989	Enzyme	Kinase
Flap endonuclease 1 inhibitor	P39748	62	1.000	1.000	Enzyme	Hydrolase
fMet-Leu-Phe receptor agonist	P21462	83	0.9996	1.000	Membrane receptor	Family A G protein-coupled receptor
fMet-Leu-Phe receptor antagonist	P21462	123	0.9892	0.99	Membrane receptor	Family A G protein-coupled receptor
Focal adhesion kinase 1 allosteric inhibitor	Q05397	12	1.000	1.000	Enzyme	Kinase
Focal adhesion kinase 1 inhibitor	Q05397	1226	0.9869	0.986	Enzyme	Kinase
Folate receptor alpha antagonist	P15328	43	1.000	1.000	Membrane receptor	NA
Folate receptor beta antagonist	P14207	29	0.9999	1.000	Membrane receptor	NA

Follicle-stimulating hormone receptor agonist	P23945	24	0.9958	0.996	Membrane receptor	Family A G protein-coupled receptor
Follicle-stimulating hormone receptor antagonist	P23945	20	0.9927	0.993	Membrane receptor	Family A G protein-coupled receptor
Folylpolyglutamate synthase, mitochondrial inhibitor	Q05932	28	1.000	1.000	Enzyme	Ligase
Free fatty acid receptor 1 agonist	O14842	1226	0.9992	0.999	Membrane receptor	Family A G protein-coupled receptor
Free fatty acid receptor 1 antagonist	O14842	106	0.9972	0.995	Membrane receptor	Family A G protein-coupled receptor
Free fatty acid receptor 2 agonist	O15552	78	0.9993	0.999	Membrane receptor	Family A G protein-coupled receptor
Free fatty acid receptor 2 antagonist	O15552	74	0.9969	0.997	Membrane receptor	Family A G protein-coupled receptor
Free fatty acid receptor 3 agonist	O14843	42	1.000	1.000	Membrane receptor	Family A G protein-coupled receptor
Free fatty acid receptor 3 antagonist	O14843	13	1.000	1.000	Membrane receptor	Family A G protein-coupled receptor
Free fatty acid receptor 4 agonist	Q5NUL3	347	0.9994	0.999	Membrane receptor	Family A G protein-coupled receptor
Frizzled-7 inhibitor	O75084	5	1.000	1.000	Membrane receptor	Frizzled family G protein-coupled receptor
Fructose-1,6-bisphosphatase 1 inhibitor	P09467	378	0.9992	0.999	Enzyme	NA
Furin inhibitor	P09958	347	0.9999	1.000	Enzyme	Protease
G protein-activated inward rectifier potassium channel 2 blocker	P48051	144	1.000	1.000	Ion channel	Voltage-gated ion channel
G protein-activated inward rectifier potassium channel 4 blocker	P48544	175	0.992	0.992	Ion channel	Voltage-gated ion channel
G protein-coupled receptor kinase 4 antagonist	P32298	6	0.8103	0.804	Enzyme	Kinase
G protein-coupled receptor kinase 5 antagonist	P34947	108	0.9743	0.975	Enzyme	Kinase
G protein-coupled receptor kinase 6 antagonist	P43250	120	0.9719	0.96	Enzyme	Kinase
G1/S-specific cyclin-D3 inhibitor	P30281	407	0.9924	0.992	Other cytosolic protein	NA
G1/S-specific cyclin-E1 inhibitor	P24864	722	0.9835	0.983	Other cytosolic protein	NA
G1/S-specific cyclin-E2 inhibitor	O96020	587	0.9947	0.994	Other cytosolic protein	NA
Galactokinase inhibitor	P51570	74	0.9849	0.985	Enzyme	Transferase
Galanin receptor type 1 antagonist	P47211	13	0.9999	1.000	Membrane receptor	Family A G protein-coupled receptor
Galanin receptor type 2 antagonist	O43603	19	0.9374	0.94	Membrane receptor	Family A G protein-coupled receptor

Galanin receptor type 3 antagonist	O60755	145	0.846	0.836	Membrane receptor	Family A G protein-coupled receptor
Gamma-aminobutyric acid receptor subunit alpha-1 antagonist	P14867	26	0.9355	0.937	Ion channel	Ligand-gated ion channel
Gamma-aminobutyric acid receptor subunit alpha-2 antagonist	P47869	421	0.9972	0.997	Ion channel	Ligand-gated ion channel
Gamma-aminobutyric acid receptor subunit alpha-3 antagonist	P34903	20	0.9235	0.925	Ion channel	Ligand-gated ion channel
Gamma-aminobutyric acid receptor subunit alpha-4 antagonist	P48169	12	0.9964	0.997	Ion channel	Ligand-gated ion channel
Gamma-aminobutyric acid receptor subunit alpha-5 antagonist	P31644	384	0.9938	0.994	Ion channel	Ligand-gated ion channel
Gamma-aminobutyric acid receptor subunit alpha-6 antagonist	Q16445	61	0.9939	0.994	Ion channel	Ligand-gated ion channel
Gamma-aminobutyric acid receptor subunit beta-2 agonist	P47870	68	0.9808	0.981	Ion channel	Ligand-gated ion channel
Gamma-aminobutyric acid receptor subunit beta-2 antagonist	P47870	308	0.9897	0.99	Ion channel	Ligand-gated ion channel
Gamma-aminobutyric acid receptor subunit gamma-2 antagonist	P18507	584	0.9969	0.997	Ion channel	Ligand-gated ion channel
Gamma-aminobutyric acid receptor subunit rho-1 agonist	P24046	15	0.999	0.999	Ion channel	Ligand-gated ion channel
Gamma-aminobutyric acid receptor subunit rho-1 antagonist	P24046	6	1.000	1.000	Ion channel	Ligand-gated ion channel
Gamma-aminobutyric acid type B receptor subunit 1 agonist	Q9UBS5	5	0.9815	0.98	Membrane receptor	Family C G protein-coupled receptor
Gamma-aminobutyric acid type B receptor subunit 1 antagonist	Q9UBS5	27	0.9956	0.996	Membrane receptor	Family C G protein-coupled receptor
Gamma-butyrobetaine dioxygenase inhibitor	O75936	29	0.9969	0.997	Enzyme	Oxidoreductase
Gamma-secretase subunit APH-1B inhibitor	Q8WW43	1673	0.9957	0.996	Enzyme	NA
Gap junction beta-2 protein inhibitor	P29033	9	1.000	1.000	Unclassified protein	NA
Gastric inhibitory polypeptide inhibitor	P09681	7	1.000	1.000	Secreted protein	NA
Gastric inhibitory polypeptide receptor agonist	P48546	15	0.9999	1.000	Membrane receptor	Family B G protein-coupled receptor
Gastric inhibitory polypeptide receptor antagonist	P48546	92	0.9999	1.000	Membrane receptor	Family B G protein-coupled receptor
Gastricsin inhibitor	P20142	28	0.9998	1.000	Enzyme	Protease
Gastrin/cholecystokinin type B receptor agonist	P32239	30	0.997	0.997	Membrane receptor	Family A G protein-coupled receptor
Gastrin/cholecystokinin type B receptor antagonist	P32239	765	0.9987	0.999	Membrane receptor	Family A G protein-coupled receptor

Gastrin-releasing peptide receptor antagonist	P30550	31	0.9992	0.999	Membrane receptor	Family A G protein-coupled receptor
Geranylgeranyl pyrophosphate synthase inhibitor	O95749	65	0.9873	0.987	Enzyme	Transferase
Geranylgeranyl transferase type-1 subunit beta inhibitor	P53609	411	0.9957	0.996	Enzyme	Transferase
Geranylgeranyl transferase type-2 subunit alpha inhibitor	Q92696	8	1.000	1.000	Enzyme	Transferase
Ghrelin O-acyltransferase inhibitor	Q96T53	4	0.8276	0.829	Enzyme	Transferase
Glucagon inhibitor	P01275	82	1.000	1.000	Secreted protein	NA
Glucagon receptor agonist	P47871	49	0.9999	1.000	Membrane receptor	Family B G protein-coupled receptor
Glucagon receptor antagonist	P47871	907	0.9957	0.996	Membrane receptor	Family B G protein-coupled receptor
Glucagon-like peptide 1 receptor agonist	P43220	451	0.9995	1.000	Membrane receptor	Family B G protein-coupled receptor
Glucagon-like peptide 1 receptor antagonist	P43220	64	0.9742	0.975	Membrane receptor	Family B G protein-coupled receptor
Glucagon-like peptide 2 receptor agonist	O95838	37	1.000	1.000	Membrane receptor	Family B G protein-coupled receptor
Glucocorticoid receptor agonist	P04150	433	0.9995	1.000	Transcription factor	Nuclear receptor
Glucocorticoid receptor antagonist	P04150	1679	0.9971	0.997	Transcription factor	Nuclear receptor
Glucokinase regulatory protein inhibitor	Q14397	103	1.000	1.000	Enzyme	NA
Glucose-6-phosphatase catalytic subunit 1 inhibitor	P35575	13	1.000	1.000	Enzyme	Phosphatase
Glucose-6-phosphatase inhibitor	P35575	13	1.000	1.000	Enzyme	Phosphatase
Glucose-6-phosphate 1-dehydrogenase inhibitor	P11413	20	1.000	1.000	Enzyme	Oxidoreductase
Glucose-6-phosphate exchanger SLC37A4 inhibitor	O43826	11	0.9999	1.000	Transporter	Electrochemical transporter
Glucose-dependent insulinotropic receptor agonist	Q8TDV5	1044	0.9974	0.997	Membrane receptor	Family A G protein-coupled receptor
Glucose-dependent insulinotropic receptor antagonist	Q8TDV5	76	0.9983	0.998	Membrane receptor	Family A G protein-coupled receptor
Glutamate carboxypeptidase 2 inhibitor	Q04609	229	0.9996	1.000	Enzyme	Protease
Glutamate receptor 1 agonist	P42261	10	0.981	0.981	Ion channel	Ligand-gated ion channel
Glutamate receptor 1 antagonist	P42261	65	0.9612	0.962	Ion channel	Ligand-gated ion channel
Glutamate receptor 2 agonist	P42262	8	0.9611	0.963	Ion channel	Ligand-gated ion channel
Glutamate receptor 2 antagonist	P42262	29	0.9986	0.999	Ion channel	Ligand-gated ion channel
Glutamate receptor 3 antagonist	P42263	10	0.9485	0.949	Ion channel	Ligand-gated ion

						channel
Glutamate receptor 4 antagonist	P48058	67	0.9712	0.971	Ion channel	Ligand-gated ion channel
Glutamate receptor ionotropic, kainate 1 agonist	P39086	3	0.9992	0.999	Ion channel	Ligand-gated ion channel
Glutamate receptor ionotropic, kainate 1 antagonist	P39086	121	0.9997	1.000	Ion channel	Ligand-gated ion channel
Glutamate receptor ionotropic, kainate 2 antagonist	Q13002	70	0.9999	1.000	Ion channel	Ligand-gated ion channel
Glutamate receptor ionotropic, kainate 3 antagonist	Q13003	30	0.9967	0.997	Ion channel	Ligand-gated ion channel
Glutamate receptor ionotropic, kainate 5 antagonist	Q16478	6	0.9966	0.997	Ion channel	Ligand-gated ion channel
Glutamate receptor ionotropic, NMDA 1 antagonist	Q05586	72	1.000	1.000	Ion channel	Ligand-gated ion channel
Glutamate receptor ionotropic, NMDA 2A antagonist	Q12879	66	0.9814	0.982	Ion channel	Ligand-gated ion channel
Glutamate receptor ionotropic, NMDA 2B antagonist	Q13224	276	0.9953	0.992	Ion channel	Ligand-gated ion channel
Glutamate receptor ionotropic, NMDA 2C antagonist	Q14957	254	0.9928	0.993	Ion channel	Ligand-gated ion channel
Glutamate--cysteine ligase catalytic subunit inhibitor	P48506	3	1.000	1.000	Enzyme	Ligase
Glutaminase kidney isoform, mitochondrial inhibitor	O94925	671	0.9979	0.998	Enzyme	Hydrolase
Glutaminase liver isoform, mitochondrial inhibitor	Q9UI32	16	0.9999	1.000	Enzyme	Hydrolase
Glutamine--tRNA ligase inhibitor	P47897	3	0.9998	1.000	Enzyme	Ligase
Glutaminyl-peptide cyclotransferase inhibitor	Q16769	401	0.9991	0.999	Enzyme	Aminoacyltransferase
Glutamyl aminopeptidase inhibitor	Q07075	35	0.9999	1.000	Enzyme	Protease
Glutathione hydrolase 1 proenzyme inhibitor	P19440	5	1.000	1.000	Enzyme	NA
Glutathione reductase, mitochondrial inhibitor	P00390	33	0.9675	0.968	Enzyme	Oxidoreductase
Glutathione S-transferase A1 inhibitor	P08263	17	0.9997	1.000	Enzyme	NA
Glutathione S-transferase A2 inhibitor	P09210	3	0.9735	0.974	Enzyme	Transferase
Glutathione S-transferase Mu 1 inhibitor	P09488	3	0.9748	0.975	Enzyme	Transferase
Glutathione S-transferase Mu 2 inhibitor	P28161	36	1.000	1.000	Enzyme	Transferase
Glutathione S-transferase omega-1 inhibitor	P78417	70	0.9846	0.985	Enzyme	NA
Glutathione S-transferase P inhibitor	P09211	84	0.9903	0.99	Enzyme	Transferase
Glyceraldehyde-3-phosphate dehydrogenase inhibitor	P04406	69	0.9092	0.908	Enzyme	Oxidoreductase
Glycine receptor subunit alpha-1 antagonist	P23415	25	0.9887	0.989	Ion channel	Ligand-gated ion channel

Glycogen [starch] synthase, muscle inhibitor	P13807	4	1.000	1.000	Enzyme	NA
Glycogen debranching enzyme inhibitor	P35573	5	1.000	1.000	Enzyme	NA
Glycogen phosphorylase, brain form inhibitor	P11216	4	0.8135	0.815	Enzyme	NA
Glycogen phosphorylase, liver form inhibitor	P06737	441	0.998	0.998	Enzyme	NA
Glycogen phosphorylase, muscle form inhibitor	P11217	97	0.9966	0.997	Enzyme	NA
Glycogen synthase kinase-3 alpha inhibitor	P49840	350	0.9733	0.973	Enzyme	Kinase
Glycogen synthase kinase-3 beta allosteric inhibitor	P49841	17	1.000	1.000	Enzyme	Kinase
Glycogen synthase kinase-3 beta inhibitor	P49841	2817	0.9802	0.98	Enzyme	Kinase
Glycylpeptide N-tetradecanoyltransferase 1 inhibitor	P30419	370	0.9987	0.999	Enzyme	Transferase
Glycylpeptide N-tetradecanoyltransferase 2 inhibitor	O60551	10	0.9852	0.985	Enzyme	Transferase
Gonadotropin-releasing hormone receptor agonist	P30968	37	0.9574	0.958	Membrane receptor	Family A G protein-coupled receptor
Gonadotropin-releasing hormone receptor antagonist	P30968	1169	0.9989	0.999	Membrane receptor	Family A G protein-coupled receptor
G-protein coupled bile acid receptor 1 agonist	Q8TDU6	523	0.9986	0.999	Membrane receptor	Family A G protein-coupled receptor
G-protein coupled estrogen receptor 1 agonist	Q99527	5	0.8729	0.875	Membrane receptor	Family A G protein-coupled receptor
G-protein coupled estrogen receptor 1 antagonist	Q99527	3	0.9465	0.948	Membrane receptor	Family A G protein-coupled receptor
G-protein coupled receptor 183 antagonist	P32249	22	0.9996	1.000	Membrane receptor	Family A G protein-coupled receptor
G-protein coupled receptor 35 agonist	Q9HC97	194	0.9962	0.996	Membrane receptor	Family A G protein-coupled receptor
G-protein coupled receptor 35 antagonist	Q9HC97	53	0.9857	0.986	Membrane receptor	Family A G protein-coupled receptor
G-protein coupled receptor 39 agonist	O43194	65	0.9653	0.967	Membrane receptor	Family A G protein-coupled receptor
G-protein coupled receptor 4 antagonist	P46093	71	1.000	1.000	Membrane receptor	Family A G protein-coupled receptor
G-protein coupled receptor 52 agonist	Q9Y2T5	45	1.000	1.000	Membrane receptor	Family A G protein-coupled receptor
G-protein coupled receptor 55 agonist	Q9Y2T6	82	0.9905	0.991	Membrane receptor	Family A G protein-coupled receptor
G-protein coupled receptor 55 antagonist	Q9Y2T6	66	0.9991	0.999	Membrane receptor	Family A G protein-coupled receptor
G-protein coupled receptor 6 agonist	P46095	32	1.000	1.000	Membrane receptor	Family A G protein-coupled receptor
G-protein coupled receptor 6 antagonist	P46095	600	1.000	1.000	Membrane receptor	Family A G protein-coupled receptor

G-protein coupled receptor 84 agonist	Q9NQS5	66	0.9998	1.000	Membrane receptor	Family A G protein-coupled receptor
G-protein coupled receptor 84 antagonist	Q9NQS5	328	0.9999	1.000	Membrane receptor	Family A G protein-coupled receptor
Granzyme B inhibitor	P10144	22	0.9974	0.997	Enzyme	Protease
Group 10 secretory phospholipase A2 inhibitor	O15496	93	0.9931	0.993	Enzyme	Hydrolase
Group IID secretory phospholipase A2 inhibitor	Q9UNK4	15	0.9987	0.999	Enzyme	Hydrolase
Group IIE secretory phospholipase A2 inhibitor	Q9NZK7	14	1.000	1.000	Enzyme	Hydrolase
Group IIF secretory phospholipase A2 inhibitor	Q9BZM2	9	1.000	1.000	Enzyme	Hydrolase
Growth factor receptor-bound protein 2 antagonist	P62993	155	0.9975	0.998	Other cytosolic protein	NA
Growth hormone secretagogue receptor type 1 agonist	Q92847	674	0.9978	0.997	Membrane receptor	Family A G protein-coupled receptor
Growth hormone secretagogue receptor type 1 antagonist	Q92847	561	0.9931	0.993	Membrane receptor	Family A G protein-coupled receptor
Growth/differentiation factor 8 inhibitor	O14793	7	0.9991	0.999	Secreted protein	NA
GTPase HRas inhibitor	P01112	29	0.9681	0.968	Other cytosolic protein	NA
GTPase KRas inhibitor	P01116	162	0.9934	0.994	Enzyme	Hydrolase
Guanine deaminase inhibitor	Q9Y2T3	12	1.000	1.000	Enzyme	Hydrolase
Guanine nucleotide-binding protein G(i) subunit alpha inhibitor	P08754	6	0.9999	1.000	Other membrane protein	NA
Guanine nucleotide-binding protein G(i) subunit alpha-1 inhibitor	P63096	6	0.9999	1.000	Other membrane protein	NA
Guanine nucleotide-binding protein G(I)/G(S)/G(T) subunit beta-1 inhibitor	P62873	19	0.9889	0.978	Unclassified protein	NA
Guanine nucleotide-binding protein G(o) subunit alpha inhibitor	P09471	7	1.000	1.000	Other membrane protein	NA
Guanine nucleotide-binding protein subunit alpha-11 inhibitor	P29992	6	1.000	1.000	Unclassified protein	NA
Guanylate cyclase soluble subunit beta-2 inhibitor	O75343	295	0.9996	1.000	Enzyme	Lyase
Heat shock factor protein 1 inhibitor	Q00613	34	0.9455	0.946	Other cytosolic protein	NA
Heat shock protein 75 kDa, mitochondrial inhibitor	Q12931	21	0.9514	0.954	Other cytosolic protein	NA
Heat shock protein beta-1 inhibitor	P04792	10	0.8762	0.876	Other cytosolic protein	NA
Heat shock protein HSP 90-alpha inhibitor	P07900	1169	0.993	0.993	Other cytosolic protein	NA
Heat shock protein HSP 90-beta inhibitor	P08238	359	0.994	0.993	Other cytosolic protein	NA
Heat shock-related 70 kDa protein 2 inhibitor	P54652	9	0.9294	0.93	Other cytosolic protein	NA
Hematopoietic prostaglandin D synthase inhibitor	O60760	248	0.9898	0.99	Enzyme	Transferase
Heme oxygenase 1 inhibitor	P09601	6	0.9991	0.999	Enzyme	Oxidoreductase
Heparanase inhibitor	Q9Y251	209	0.9941	0.994	Enzyme	Hydrolase
Hepatic triacylglycerol lipase inhibitor	P11150	60	0.9759	0.977	Enzyme	NA

Hepatocyte growth factor activator inhibitor	Q04756	112	0.9986	0.999	Enzyme	Protease
Hepatocyte growth factor inhibitor	P14210	4	1.000	1.000	Unclassified protein	NA
Hepatocyte growth factor receptor antagonist	P08581	3153	0.9868	0.987	Enzyme	Kinase
Hepatocyte growth factor-like protein inhibitor	P26927	3	1.000	1.000	Secreted protein	NA
Hepatocyte nuclear factor 4-alpha inhibitor	P41235	20	0.9999	1.000	Unclassified protein	NA
Hexokinase HKDC1 inhibitor	Q2TB90	99	0.9336	0.932	Enzyme	Transferase
Hexokinase-1 inhibitor	P19367	20	0.9858	0.986	Enzyme	NA
Hexokinase-2 inhibitor	P52789	25	0.9881	0.988	Enzyme	NA
Hexokinase-4 activator	P35557	28	1.000	1.000	Enzyme	NA
Hexokinase-4 inhibitor	P35557	88	0.9791	0.979	Enzyme	NA
High affinity cAMP-specific 3',5'-cyclic phosphodiesterase 7A inhibitor	Q13946	540	0.9865	0.986	Enzyme	Phosphodiesterase
High affinity cAMP-specific and IBMX-insensitive 3',5'-cyclic phosphodiesterase 8A inhibitor	O60658	14	0.8353	0.839	Enzyme	Phosphodiesterase
High affinity cAMP-specific and IBMX-insensitive 3',5'-cyclic phosphodiesterase 8B inhibitor	O95263	121	0.997	0.997	Enzyme	Phosphodiesterase
High affinity cGMP-specific 3',5'-cyclic phosphodiesterase 9A inhibitor	O76083	395	0.9905	0.991	Enzyme	Phosphodiesterase
High affinity choline transporter 1 inhibitor	Q9GZV3	24	1.000	1.000	Transporter	Electrochemical transporter
High affinity nerve growth factor receptor agonist	P04629	702	0.9995	1.000	Enzyme	Kinase
High affinity nerve growth factor receptor antagonist	P04629	1184	0.9716	0.971	Enzyme	Kinase
Histamine H1 receptor agonist	P35367	17	0.9711	0.97	Membrane receptor	Family A G protein-coupled receptor
Histamine H1 receptor antagonist	P35367	807	0.9838	0.984	Membrane receptor	Family A G protein-coupled receptor
Histamine H2 receptor agonist	P25021	89	0.9999	1.000	Membrane receptor	Family A G protein-coupled receptor
Histamine H2 receptor antagonist	P25021	136	0.9615	0.962	Membrane receptor	Family A G protein-coupled receptor
Histamine H3 receptor agonist	Q9Y5N1	388	0.9955	0.995	Membrane receptor	Family A G protein-coupled receptor
Histamine H3 receptor antagonist	Q9Y5N1	2048	0.9926	0.993	Membrane receptor	Family A G protein-coupled receptor
Histamine H4 receptor agonist	Q9H3N8	245	0.9973	0.997	Membrane receptor	Family A G protein-coupled receptor
Histamine H4 receptor antagonist	Q9H3N8	265	0.9867	0.987	Membrane receptor	Family A G protein-coupled receptor
Histamine N-methyltransferase inhibitor	P50135	4	0.8908	0.895	Enzyme	Transferase
Histone acetyltransferase KAT2A inhibitor	Q92830	11	0.8398	0.841	Epigenetic regulator	Reader; Writer

Histone acetyltransferase KAT2B inhibitor	Q92831	86	0.9958	0.974	Epigenetic regulator	Reader; Writer
Histone acetyltransferase KAT6A inhibitor	Q92794	222	1.000	1.000	Enzyme	NA
Histone acetyltransferase KAT8 inhibitor	Q9H7Z6	5	0.9999	1.000	Epigenetic regulator	Reader; Writer
Histone acetyltransferase p300 inhibitor	Q09472	247	0.9762	0.977	Epigenetic regulator	Reader; Writer
Histone chaperone ASF1A inhibitor	Q9Y294	3	0.9987	0.999	Other nuclear protein	NA
Histone deacetylase 1 inhibitor	Q13547	3501	0.9952	0.995	Epigenetic regulator	Eraser
Histone deacetylase 11 inhibitor	Q96DB2	230	0.9974	0.997	Epigenetic regulator	Eraser
Histone deacetylase 2 inhibitor	Q92769	1311	0.9948	0.995	Epigenetic regulator	Eraser
Histone deacetylase 3 inhibitor	O15379	938	0.9955	0.996	Epigenetic regulator	Eraser
Histone deacetylase 4 inhibitor	P56524	526	0.9949	0.995	Epigenetic regulator	Eraser
Histone deacetylase 5 inhibitor	Q9UQL6	1758	0.9936	0.994	Epigenetic regulator	Eraser
Histone deacetylase 6 inhibitor	Q9UBN7	2202	0.9958	0.996	Epigenetic regulator	Eraser
Histone deacetylase 7 inhibitor	Q8WUI4	144	0.9885	0.988	Epigenetic regulator	Eraser
Histone deacetylase 8 inhibitor	Q9BY41	1294	0.9944	0.994	Epigenetic regulator	Eraser
Histone deacetylase 9 inhibitor	Q9UKV0	120	0.9747	0.975	Epigenetic regulator	Eraser
Histone H1.0 inhibitor	P07305	7	0.9812	0.983	Other nuclear protein	NA
Histone lysine demethylase PHF8 inhibitor	Q9UPP1	63	0.9931	0.993	Epigenetic regulator	Eraser; Reader
Histone-arginine methyltransferase CARM1 inhibitor	Q86X55	154	0.9876	0.988	Epigenetic regulator	Writer
Histone-lysine N-methyltransferase 2A inhibitor	Q03164	7	0.8102	0.814	Epigenetic regulator	Reader; Writer
Histone-lysine N-methyltransferase EHMT1 inhibitor	Q9H9B1	120	0.9973	0.996	Epigenetic regulator	Writer
Histone-lysine N-methyltransferase EHMT2 inhibitor	Q96KQ7	258	0.9969	0.996	Epigenetic regulator	Writer
Histone-lysine N-methyltransferase EZH1 inhibitor	Q92800	29	0.9773	0.91	Epigenetic regulator	Writer
Histone-lysine N-methyltransferase EZH2 inhibitor	Q15910	414	0.9994	0.999	Epigenetic regulator	Writer
Histone-lysine N-methyltransferase NSD2 inhibitor	O96028	3	0.9878	0.988	Epigenetic regulator	Writer
Histone-lysine N-methyltransferase SETD7 inhibitor	Q8WTS6	12	0.9531	0.822	Epigenetic regulator	Writer
Histone-lysine N-methyltransferase SMYD3 inhibitor	Q9H7B4	84	1.000	1.000	Epigenetic regulator	Writer
Histone-lysine N-methyltransferase SUV39H1 inhibitor	O43463	7	0.8718	0.604	Epigenetic regulator	Reader; Writer
Histone-lysine N-methyltransferase SUV39H2 inhibitor	Q9H5I1	437	1.000	1.000	Epigenetic regulator	Reader; Writer
Histone-lysine N-methyltransferase, H3 lysine-79 specific inhibitor	Q8TEK3	101	0.9956	0.996	Epigenetic regulator	Writer
HLA class I histocompatibility antigen, A alpha chain inhibitor	P04439	149	0.9997	1.000	Surface antigen	NA
HLA class II histocompatibility antigen gamma chain inhibitor	P04233	37	0.9869	0.988	Surface antigen	NA
HLA class II histocompatibility antigen, DR beta 3 chain inhibitor	P79483	16	0.9999	1.000	Surface antigen	NA

HLA class II histocompatibility antigen, DRB1 beta chain inhibitor	P01911	3	1.000	1.000	Surface antigen	NA
HLA class II histocompatibility antigen, DRB1-1 beta chain inhibitor	P04229	38	1.000	1.000	Surface antigen	NA
Homeodomain-interacting protein kinase 1 inhibitor	Q86Z02	14	0.882	0.885	Enzyme	Kinase
Homeodomain-interacting protein kinase 2 inhibitor	Q9H2X6	19	0.8681	0.866	Enzyme	Kinase
Hormone-sensitive lipase inhibitor	Q05469	251	1.000	1.000	Enzyme	Hydrolase
Huntingtin inhibitor	P42858	15	0.9677	0.968	Unclassified protein	NA
Hydroxycarboxylic acid receptor 1 agonist	Q9BXC0	302	0.9999	1.000	Membrane receptor	Family A G protein-coupled receptor
Hydroxycarboxylic acid receptor 2 agonist	Q8TDS4	425	0.9968	0.996	Membrane receptor	Family A G protein-coupled receptor
Hydroxycarboxylic acid receptor 2 antagonist	Q8TDS4	30	0.9886	0.989	Membrane receptor	Family A G protein-coupled receptor
Hydroxycarboxylic acid receptor 3 agonist	P49019	103	0.9997	1.000	Membrane receptor	Family A G protein-coupled receptor
Hydroxycarboxylic acid receptor 3 antagonist	P49019	15	1.000	1.000	Membrane receptor	Family A G protein-coupled receptor
Hypoxanthine-guanine phosphoribosyltransferase inhibitor	P00492	104	0.9996	1.000	Enzyme	Transferase
Hypoxia-inducible factor 1-alpha inhibitor	Q16665	197	0.9735	0.974	Transcription factor	NA
IgG receptor FcRn large subunit p51 antagonist	P55899	70	1.000	1.000	Unclassified protein	NA
Ileal sodium/bile acid cotransporter inhibitor	Q12908	253	0.9997	1.000	Transporter	Electrochemical transporter
Indoleamine 2,3-dioxygenase 1 inhibitor	P14902	1456	0.9935	0.993	Enzyme	Oxidoreductase
Indoleamine 2,3-dioxygenase 2 inhibitor	Q6ZQW0	20	0.9998	1.000	Enzyme	Oxidoreductase
Indolethylamine N-methyltransferase inhibitor	O95050	15	0.9994	1.000	Enzyme	Transferase
Induced myeloid leukemia cell differentiation protein Mcl-1 inhibitor	Q07820	582	0.9896	0.989	Other cytosolic protein	NA
Inhibitor of nuclear factor kappa-B kinase subunit alpha inhibitor	O15111	340	0.9894	0.989	Enzyme	Kinase
Inhibitor of nuclear factor kappa-B kinase subunit beta inhibitor	O14920	894	0.986	0.985	Enzyme	Kinase
Inhibitor of nuclear factor kappa-B kinase subunit epsilon inhibitor	Q14164	414	0.9942	0.994	Enzyme	Kinase
Inosine-5'-monophosphate dehydrogenase 1 inhibitor	P20839	140	0.9995	1.000	Enzyme	Oxidoreductase
Inosine-5'-monophosphate dehydrogenase 2 inhibitor	P12268	619	0.9987	0.999	Enzyme	Oxidoreductase
Inositol 1,4,5-trisphosphate receptor type 1 antagonist	Q14643	4	1.000	1.000	Ion channel	Ligand-gated ion channel
Inositol 1,4,5-trisphosphate receptor type 3 antagonist	Q14573	3	1.000	1.000	Ion channel	Ligand-gated ion channel
Inositol hexakisphosphate kinase 1 inhibitor	Q92551	14	1.000	1.000	Enzyme	Transferase
Inositol hexakisphosphate kinase 2 inhibitor	Q9UHH9	11	0.9683	0.97	Enzyme	Transferase

Inositol polyphosphate multikinase inhibitor	Q8NFU5	6	1.000	1.000	Enzyme	Transferase
Inositol polyphosphate-5-phosphatase A inhibitor	Q14642	3	0.9963	0.997	Enzyme	Phosphatase
Inositol-trisphosphate 3-kinase B inhibitor	P27987	38	1.000	1.000	Enzyme	NA
Insulin receptor antagonist	P06213	857	0.9791	0.979	Enzyme	Kinase
Insulin receptor-related protein inhibitor	P14616	11	0.8118	0.817	Enzyme	Kinase
Insulin-degrading enzyme inhibitor	P14735	88	0.8975	0.895	Enzyme	NA
Insulin-like growth factor 1 receptor agonist	P08069	5	1.000	1.000	Enzyme	Kinase
Insulin-like growth factor 1 receptor antagonist	P08069	2013	0.9854	0.985	Enzyme	Kinase
Insulin-like growth factor-binding protein 3 inhibitor	P17936	39	1.000	1.000	Secreted protein	NA
Insulin-like growth factor-binding protein 5 inhibitor	P24593	9	1.000	1.000	Secreted protein	NA
Integrin alpha-3 inhibitor	P26006	7	1.000	1.000	Membrane receptor	NA
Integrin alpha-4 inhibitor	P13612	1180	0.9982	0.998	Membrane receptor	NA
Integrin alpha-5 inhibitor	P08648	10	1.000	1.000	Membrane receptor	NA
Integrin alpha-IIb inhibitor	P08514	1279	0.9973	0.997	Membrane receptor	NA
Integrin alpha-L inhibitor	P20701	79	0.9985	0.999	Adhesion; Membrane receptor	NA
Integrin alpha-V inhibitor	P06756	1129	0.9967	0.997	Membrane receptor	NA
Integrin beta-1 inhibitor	P05556	336	0.9932	0.993	Membrane receptor	NA
Integrin beta-2 inhibitor	P05107	345	0.9996	1.000	Membrane receptor	NA
Integrin-linked protein kinase inhibitor	Q13418	160	0.9976	0.998	Enzyme	Kinase
Intercellular adhesion molecule 1 inhibitor	P05362	125	0.9883	0.988	Adhesion	NA
Interferon-induced, double-stranded RNA-activated protein kinase inhibitor	P19525	60	0.9599	0.958	Enzyme	Kinase
Interleukin-1 receptor antagonist protein antagonist	P18510	11	0.9723	0.953	Unclassified protein	NA
Interleukin-1 receptor-associated kinase 1 antagonist	P51617	46	0.8924	0.891	Enzyme	Kinase
Interleukin-1 receptor-associated kinase 3 inhibitor	Q9Y616	3	0.8373	0.84	Enzyme	Kinase
Interleukin-1 receptor-associated kinase 4 antagonist	Q9NWZ3	1199	0.9936	0.994	Enzyme	Kinase
Interleukin-15 receptor subunit alpha antagonist	Q13261	20	1.000	1.000	Membrane receptor	NA
Interleukin-2 inhibitor	P60568	11	0.9965	0.997	Secreted protein	NA
Interleukin-23 receptor antagonist	Q5VWK5	528	0.9999	1.000	Unclassified protein	NA
Interleukin-6 inhibitor	P05231	13	0.9598	0.96	Secreted protein	NA
Interleukin-6 receptor subunit beta antagonist	P40189	4	1.000	1.000	Membrane receptor	NA
Interleukin-8 inhibitor	P10145	24	0.9992	0.999	Secreted protein	NA
Intermediate conductance calcium-activated potassium channel protein 4 blocker	O15554	37	0.9882	0.988	Ion channel	Voltage-gated ion

						channel
Interstitial collagenase inhibitor	P03956	2016	0.9937	0.994	Enzyme	Protease
Intestinal-type alkaline phosphatase inhibitor	P09923	95	0.9537	0.955	Enzyme	Phosphatase
Isocitrate dehydrogenase [NADP] cytoplasmic inhibitor	O75874	1264	0.9966	0.997	Enzyme	Oxidoreductase
Isocitrate dehydrogenase [NADP], mitochondrial inhibitor	P48735	248	0.9975	0.997	Enzyme	Oxidoreductase
Isoleucine--tRNA ligase, cytoplasmic inhibitor	P41252	20	0.9995	1.000	Enzyme	Ligase
Kallikrein-1 inhibitor	P06870	181	0.9744	0.974	Enzyme	Protease
Kallikrein-14 inhibitor	Q9P0G3	24	0.9723	0.973	Enzyme	Protease
Kallikrein-2 inhibitor	P20151	15	0.9755	0.976	Enzyme	Protease
Kallikrein-4 inhibitor	Q9Y5K2	20	0.9356	0.936	Enzyme	Protease
Kallikrein-5 inhibitor	Q9Y337	97	0.9866	0.986	Enzyme	Protease
Kallikrein-6 inhibitor	Q92876	34	0.9867	0.987	Enzyme	Protease
Kallikrein-7 inhibitor	P49862	111	0.9896	0.99	Enzyme	Protease
Kallikrein-8 inhibitor	O60259	28	0.9542	0.957	Enzyme	Protease
Kappa-type opioid receptor agonist	P41145	1022	0.9917	0.991	Membrane receptor	Family A G protein-coupled receptor
Kappa-type opioid receptor antagonist	P41145	1637	0.9847	0.985	Membrane receptor	Family A G protein-coupled receptor
Kelch-like ECH-associated protein 1 inhibitor	Q14145	140	0.9928	0.993	Unclassified protein	NA
Ketohexokinase inhibitor	P50053	196	0.9992	0.999	Enzyme	Transferase
Kinesin-like protein KIF11 inhibitor	P52732	774	0.9955	0.996	Other cytosolic protein	NA
Kinesin-like protein KIF20A inhibitor	O95235	8	1.000	1.000	Other cytosolic protein	NA
Kinesin-like protein KIFC1 inhibitor	Q9BW19	17	1.000	1.000	Other cytosolic protein	NA
Kininogen-1 inhibitor	P01042	59	1.000	1.000	Unclassified protein	NA
KiSS-1 receptor agonist	Q969F8	237	0.9998	1.000	Membrane receptor	Family A G protein-coupled receptor
KiSS-1 receptor antagonist	Q969F8	38	0.9995	0.999	Membrane receptor	Family A G protein-coupled receptor
Krueppel-like factor 5 inhibitor	Q13887	5	1.000	1.000	Transcription factor	NA
Kynureninase inhibitor	Q16719	4	0.9524	0.953	Enzyme	Hydrolase
Kynurenine 3-monooxygenase inhibitor	O15229	157	0.9967	0.997	Enzyme	Oxidoreductase
Kynurenine/alpha-aminoadipate aminotransferase, mitochondrial inhibitor	Q8N5Z0	59	0.9744	0.974	Enzyme	Transferase
Kynurenine--oxoglutarate transaminase 3 inhibitor	Q6YP21	3	1.000	1.000	Enzyme	NA
Lactoylglutathione lyase inhibitor	Q04760	94	0.9978	0.998	Enzyme	Lyase

Lanosterol 14-alpha demethylase inhibitor	Q16850	29	0.9998	1.000	Enzyme	Cytochrome P450
Lanosterol synthase inhibitor	P48449	114	0.9938	0.994	Enzyme	Isomerase
Large neutral amino acids transporter small subunit 1 inhibitor	Q01650	5	0.9322	0.933	Transporter	Electrochemical transporter
Legumain inhibitor	Q99538	113	0.9997	1.000	Enzyme	Protease
Lethal(3)malignant brain tumor-like protein 1 inhibitor	Q9Y468	50	0.9693	0.97	Epigenetic regulator	Reader
Lethal(3)malignant brain tumor-like protein 3 inhibitor	Q96JM7	76	0.997	0.997	Epigenetic regulator	Reader
Leucine-rich repeat serine/threonine-protein kinase 2 inhibitor	Q5S007	1472	0.9925	0.993	Enzyme	Kinase
Leucine--tRNA ligase, cytoplasmic inhibitor	Q9P2J5	18	0.9761	0.977	Enzyme	Ligase
Leucyl-cystinyl aminopeptidase inhibitor	Q9UIQ6	137	0.9913	0.986	Enzyme	Protease
Leukocyte tyrosine kinase receptor antagonist	P29376	20	0.883	0.888	Enzyme	Kinase
Leukotriene A-4 hydrolase inhibitor	P09960	457	0.9979	0.998	Enzyme	Protease
Leukotriene B4 receptor 1 antagonist	Q15722	300	0.9981	0.998	Membrane receptor	Family A G protein-coupled receptor
Leukotriene B4 receptor 2 antagonist	Q9NPC1	237	0.9978	0.998	Membrane receptor	Family A G protein-coupled receptor
Leukotriene C4 synthase inhibitor	Q16873	81	0.9977	0.998	Enzyme	Lyase
LIM domain kinase 1 inhibitor	P53667	221	0.9838	0.984	Enzyme	Kinase
LIM domain kinase 2 inhibitor	P53671	211	0.9934	0.993	Enzyme	Kinase
Lipoprotein lipase inhibitor	P06858	11	0.8761	0.881	Enzyme	Hydrolase
Liver carboxylesterase 1 inhibitor	P23141	244	0.993	0.993	Enzyme	NA
L-lactate dehydrogenase A chain inhibitor	P00338	274	0.9731	0.973	Enzyme	NA
L-lactate dehydrogenase B chain inhibitor	P07195	105	0.9849	0.985	Enzyme	Oxidoreductase
L-lactate dehydrogenase inhibitor	V9HWB9	7	0.963	0.963	Enzyme	Oxidoreductase
Long-chain fatty acid transport protein 1 inhibitor	Q6PCB7	36	1.000	1.000	Transporter	Electrochemical transporter
Long-chain fatty acid transport protein 4 inhibitor	Q6P1M0	21	1.000	1.000	Transporter	Electrochemical transporter
Low affinity immunoglobulin epsilon Fc receptor antagonist	P06734	48	1.000	1.000	Membrane receptor	NA
Low molecular weight phosphotyrosine protein phosphatase inhibitor	P24666	49	0.9913	0.991	Enzyme	Phosphatase
Low-density lipoprotein receptor-related protein 6 antagonist	O75581	11	1.000	1.000	Unclassified protein	NA
L-selectin inhibitor	P14151	22	0.9998	1.000	Adhesion	NA
Lutropin-choriogonadotropic hormone receptor agonist	P22888	22	0.972	0.969	Membrane receptor	Family A G protein-coupled receptor
Lutropin-choriogonadotropic hormone receptor antagonist	P22888	27	1.000	1.000	Membrane receptor	Family A G protein-coupled receptor

Lymphocyte antigen 96 inhibitor	Q9Y6Y9	4	1.000	1.000	Surface antigen	NA
Lymphokine-activated killer T-cell-originated protein kinase inhibitor	Q96KB5	138	0.9915	0.992	Enzyme	Kinase
Lysine-specific demethylase 2A inhibitor	Q9Y2K7	60	0.9856	0.986	Epigenetic regulator	Eraser; Reader
Lysine-specific demethylase 2B inhibitor	Q8NHM5	96	0.9998	1.000	Epigenetic regulator	Eraser
Lysine-specific demethylase 3A inhibitor	Q9Y4C1	26	0.998	0.998	Epigenetic regulator	Eraser
Lysine-specific demethylase 4A inhibitor	O75164	175	0.9911	0.991	Epigenetic regulator	Eraser; Reader
Lysine-specific demethylase 4B inhibitor	O94953	89	0.9962	0.996	Epigenetic regulator	Eraser
Lysine-specific demethylase 4C inhibitor	Q9H3R0	418	0.9957	0.996	Epigenetic regulator	Eraser; Reader
Lysine-specific demethylase 4D inhibitor	Q6B0I6	22	0.9995	0.999	Epigenetic regulator	Eraser
Lysine-specific demethylase 4E inhibitor	B2RXH2	42	0.99	0.99	Epigenetic regulator	Eraser
Lysine-specific demethylase 5A inhibitor	P29375	247	0.9893	0.989	Epigenetic regulator	Eraser; Reader
Lysine-specific demethylase 5B inhibitor	Q9UGL1	306	0.9956	0.996	Epigenetic regulator	Eraser
Lysine-specific demethylase 5C inhibitor	P41229	135	0.9981	0.998	Epigenetic regulator	Eraser; Reader
Lysine-specific demethylase 6B inhibitor	O15054	51	0.9933	0.994	Epigenetic regulator	Eraser
Lysine-specific demethylase 7A inhibitor	Q6ZMT4	3	1.000	1.000	Epigenetic regulator	Eraser; Reader
Lysine-specific histone demethylase 1A inhibitor	O60341	638	0.9879	0.988	Epigenetic regulator	Eraser
Lysine-specific histone demethylase 1B inhibitor	Q8NB78	6	0.8471	0.848	Epigenetic regulator	Eraser
Lysine--tRNA ligase inhibitor	Q15046	81	0.9816	0.981	Enzyme	NA
Lysophosphatidic acid receptor 1 agonist	Q92633	33	0.9902	0.99	Membrane receptor	Family A G protein-coupled receptor
Lysophosphatidic acid receptor 1 antagonist	Q92633	192	0.9957	0.996	Membrane receptor	Family A G protein-coupled receptor
Lysophosphatidic acid receptor 2 agonist	Q9HBW0	22	0.9999	1.000	Membrane receptor	Family A G protein-coupled receptor
Lysophosphatidic acid receptor 2 antagonist	Q9HBW0	52	0.9961	0.996	Membrane receptor	Family A G protein-coupled receptor
Lysophosphatidic acid receptor 3 agonist	Q9UBY5	16	1.000	1.000	Membrane receptor	Family A G protein-coupled receptor
Lysophosphatidic acid receptor 3 antagonist	Q9UBY5	68	0.998	0.998	Membrane receptor	Family A G protein-coupled receptor
Lysophosphatidic acid receptor 4 agonist	Q99677	7	1.000	1.000	Membrane receptor	Family A G protein-coupled receptor
Lysophosphatidic acid receptor 4 antagonist	Q99677	3	1.000	1.000	Membrane receptor	Family A G protein-coupled receptor
Lysophosphatidic acid receptor 5 agonist	Q9H1C0	7	1.000	1.000	Membrane receptor	Family A G protein-coupled receptor
Lysophosphatidic acid receptor 5 antagonist	Q9H1C0	83	0.9993	0.999	Membrane receptor	Family A G protein-

						coupled receptor
Lysophosphatidic acid receptor 6 agonist	P43657	7	1.000	1.000	Membrane receptor	Family A G protein-coupled receptor
Lysosomal acid glucosylceramidase inhibitor	P04062	413	0.9829	0.983	Enzyme	NA
Lysosomal acid lipase/cholesteryl ester hydrolase inhibitor	P38571	8	1.000	1.000	Enzyme	Hydrolase
Lysosomal alpha-glucosidase inhibitor	P10253	5172	0.8612	0.861	Enzyme	Hydrolase
Lysosomal alpha-mannosidase inhibitor	O00754	5	0.9999	1.000	Enzyme	Hydrolase
Lysosomal protective protein inhibitor	P10619	740	0.9994	0.999	Enzyme	Protease
Lysosomal Pro-X carboxypeptidase inhibitor	P42785	342	0.9986	0.999	Enzyme	Protease
Lysyl oxidase homolog 2 inhibitor	Q9Y4K0	532	0.9993	0.999	Enzyme	Oxidoreductase
Lysyl oxidase homolog 3 inhibitor	P58215	20	0.9926	0.903	Enzyme	Oxidoreductase
Lysyl oxidase homolog 4 inhibitor	Q96JB6	6	0.9677	0.767	Enzyme	Oxidoreductase
m7GpppX diphosphatase inhibitor	Q96C86	41	0.9999	1.000	Enzyme	Hydrolase
Macrophage colony-stimulating factor 1 receptor antagonist	P07333	1238	0.9859	0.986	Enzyme	Kinase
Macrophage metalloelastase inhibitor	P39900	457	0.993	0.993	Enzyme	Protease
Macrophage migration inhibitory factor inhibitor	P14174	171	0.9709	0.971	Enzyme	NA
Macrophage-expressed gene 1 protein inhibitor	Q2M385	4	0.8695	0.871	Unclassified protein	NA
Macrophage-stimulating protein receptor antagonist	Q04912	99	0.9768	0.977	Enzyme	Kinase
Major prion protein inhibitor	P04156	7	1.000	1.000	Surface antigen	NA
Malate dehydrogenase, cytoplasmic inhibitor	P40925	19	0.9984	0.998	Enzyme	Oxidoreductase
Malate dehydrogenase, mitochondrial inhibitor	P40926	32	0.9963	0.997	Enzyme	Oxidoreductase
Malonyl-CoA decarboxylase, mitochondrial inhibitor	O95822	250	1.000	1.000	Enzyme	Lyase
Maltase-glucoamylase inhibitor	O43451	66	0.9917	0.992	Enzyme	Hydrolase
Mannan-binding lectin serine protease 1 inhibitor	P48740	3	0.9996	1.000	Enzyme	Hydrolase
Mannan-binding lectin serine protease 2 inhibitor	O00187	5	0.9993	0.998	Enzyme	Hydrolase
Mannose-6-phosphate isomerase inhibitor	P34949	102	0.9423	0.943	Enzyme	Isomerase
MAP kinase-activated protein kinase 2 inhibitor	P49137	793	0.9855	0.985	Enzyme	Kinase
MAP kinase-activated protein kinase 3 inhibitor	Q16644	14	0.9632	0.95	Enzyme	Kinase
MAP kinase-activated protein kinase 5 inhibitor	Q8IW41	60	0.8933	0.894	Enzyme	Kinase
MAP kinase-interacting serine/threonine-protein kinase 1 inhibitor	Q9BUB5	325	0.9843	0.984	Enzyme	Kinase
MAP kinase-interacting serine/threonine-protein kinase 2 inhibitor	Q9HBH9	639	0.9928	0.993	Enzyme	Kinase
MAP/microtubule affinity-regulating kinase 3 inhibitor	P27448	62	0.9373	0.938	Enzyme	Kinase
MAP/microtubule affinity-regulating kinase 4 inhibitor	Q96L34	24	0.9321	0.933	Enzyme	Kinase

Mas-related G-protein coupled receptor member X1 agonist	Q96LB2	48	0.9999	1.000	Membrane receptor	Family A G protein-coupled receptor
Mas-related G-protein coupled receptor member X1 antagonist	Q96LB2	31	0.9998	1.000	Membrane receptor	Family A G protein-coupled receptor
Mas-related G-protein coupled receptor member X2 agonist	Q96LB1	8	0.8625	0.867	Membrane receptor	Family A G protein-coupled receptor
Mast cell carboxypeptidase A inhibitor	P15088	3	1.000	1.000	Enzyme	Protease
Mast/stem cell growth factor receptor Kit antagonist	P10721	1267	0.9775	0.977	Enzyme	Kinase
Maternal embryonic leucine zipper kinase inhibitor	Q14680	815	0.9854	0.985	Enzyme	Kinase
Matrilysin inhibitor	P09237	388	0.9968	0.997	Enzyme	Protease
Matrix metalloproteinase-14 inhibitor	P50281	546	0.9898	0.99	Enzyme	Protease
Matrix metalloproteinase-15 inhibitor	P51511	19	0.9991	0.992	Enzyme	Protease
Matrix metalloproteinase-16 inhibitor	P51512	48	0.9992	0.999	Enzyme	Protease
Matrix metalloproteinase-17 inhibitor	Q9ULZ9	18	1.000	1.000	Enzyme	Protease
Matrix metalloproteinase-25 inhibitor	Q9NPA2	9	0.9984	0.998	Enzyme	Protease
Matrix metalloproteinase-26 inhibitor	Q9NRE1	13	0.9995	1.000	Enzyme	Protease
Matrix metalloproteinase-9 inhibitor	P14780	2078	0.9894	0.989	Enzyme	Protease
MBT domain-containing protein 1 inhibitor	Q05BQ5	14	1.000	1.000	Epigenetic regulator	Reader
Melanin-concentrating hormone receptor 1 agonist	Q99705	8	0.992	0.992	Membrane receptor	Family A G protein-coupled receptor
Melanin-concentrating hormone receptor 1 antagonist	Q99705	2127	0.9926	0.993	Membrane receptor	Family A G protein-coupled receptor
Melanin-concentrating hormone receptor 2 antagonist	Q969V1	74	0.9995	1.000	Membrane receptor	Family A G protein-coupled receptor
Melanocortin receptor 3 agonist	P41968	352	0.9983	0.998	Membrane receptor	Family A G protein-coupled receptor
Melanocortin receptor 3 antagonist	P41968	269	0.9961	0.996	Membrane receptor	Family A G protein-coupled receptor
Melanocortin receptor 4 agonist	P32245	876	0.998	0.998	Membrane receptor	Family A G protein-coupled receptor
Melanocortin receptor 4 antagonist	P32245	1124	0.9969	0.997	Membrane receptor	Family A G protein-coupled receptor
Melanocortin receptor 5 agonist	P33032	124	0.9989	0.999	Membrane receptor	Family A G protein-coupled receptor
Melanocortin receptor 5 antagonist	P33032	641	0.9981	0.998	Membrane receptor	Family A G protein-coupled receptor
Melanocyte-stimulating hormone receptor agonist	Q01726	529	0.9979	0.998	Membrane receptor	Family A G protein-coupled receptor
Melanocyte-stimulating hormone receptor antagonist	Q01726	310	0.9959	0.996	Membrane receptor	Family A G protein-

						coupled receptor
Melatonin receptor type 1A agonist	P48039	79	0.9923	0.992	Membrane receptor	Family A G protein-coupled receptor
Melatonin receptor type 1A antagonist	P48039	540	0.9981	0.998	Membrane receptor	Family A G protein-coupled receptor
Melatonin receptor type 1B agonist	P49286	88	0.9959	0.995	Membrane receptor	Family A G protein-coupled receptor
Melatonin receptor type 1B antagonist	P49286	579	0.9978	0.998	Membrane receptor	Family A G protein-coupled receptor
Membrane metallo-endopeptidase-like 1 inhibitor	Q495T6	56	0.9999	1.000	Enzyme	Hydrolase
Membrane primary amine oxidase inhibitor	Q16853	209	0.9984	0.999	Enzyme	Oxidoreductase
Membrane-associated guanylate kinase, WW and PDZ domain-containing protein 3 inhibitor	Q5TCQ9	6	1.000	1.000	Enzyme	NA
Membrane-associated tyrosine- and threonine-specific cdc2-inhibitory kinase inhibitor	Q99640	31	0.956	0.957	Enzyme	Kinase
Membrane-bound transcription factor site-1 protease inhibitor	Q14703	19	1.000	1.000	Enzyme	Protease
Menin inhibitor	O00255	184	0.9945	0.995	Unclassified protein	NA
Meprin A subunit alpha inhibitor	Q16819	7	0.9999	1.000	Enzyme	Hydrolase
Meprin A subunit beta inhibitor	Q16820	57	0.9977	0.998	Enzyme	Hydrolase
Metabotropic glutamate receptor 1 agonist	Q13255	15	0.9946	0.995	Membrane receptor	Family C G protein-coupled receptor
Metabotropic glutamate receptor 1 antagonist	Q13255	523	0.9921	0.992	Membrane receptor	Family C G protein-coupled receptor
Metabotropic glutamate receptor 2 agonist	Q14416	467	0.9992	0.999	Membrane receptor	Family C G protein-coupled receptor
Metabotropic glutamate receptor 2 antagonist	Q14416	858	0.9979	0.998	Membrane receptor	Family C G protein-coupled receptor
Metabotropic glutamate receptor 3 agonist	Q14832	51	0.9925	0.993	Membrane receptor	Family C G protein-coupled receptor
Metabotropic glutamate receptor 3 antagonist	Q14832	75	0.993	0.993	Membrane receptor	Family C G protein-coupled receptor
Metabotropic glutamate receptor 4 agonist	Q14833	50	0.9719	0.972	Membrane receptor	Family C G protein-coupled receptor
Metabotropic glutamate receptor 4 antagonist	Q14833	9	0.9997	1.000	Membrane receptor	Family C G protein-coupled receptor
Metabotropic glutamate receptor 5 agonist	P41594	321	0.9931	0.993	Membrane receptor	Family C G protein-coupled receptor
Metabotropic glutamate receptor 5 antagonist	P41594	1128	0.9948	0.995	Membrane receptor	Family C G protein-coupled receptor
Metabotropic glutamate receptor 6 agonist	O15303	35	0.9999	1.000	Membrane receptor	Family C G protein-coupled receptor

Metabotropic glutamate receptor 6 antagonist	O15303	23	0.9639	0.964	Membrane receptor	Family C G protein-coupled receptor
Metabotropic glutamate receptor 7 agonist	Q14831	15	0.9386	0.796	Membrane receptor	Family C G protein-coupled receptor
Metabotropic glutamate receptor 7 antagonist	Q14831	4	1.000	1.000	Membrane receptor	Family C G protein-coupled receptor
Metabotropic glutamate receptor 8 agonist	O00222	45	0.9998	0.969	Membrane receptor	Family C G protein-coupled receptor
Metabotropic glutamate receptor 8 antagonist	O00222	13	0.9997	1.000	Membrane receptor	Family C G protein-coupled receptor
Methionine aminopeptidase 1 inhibitor	P53582	124	0.9954	0.996	Enzyme	Protease
Methionine aminopeptidase 2 inhibitor	P50579	653	0.9987	0.999	Enzyme	Protease
Methionine synthase inhibitor	Q99707	3	1.000	1.000	Enzyme	Transferase
Methionine--tRNA ligase, cytoplasmic inhibitor	P56192	59	0.9999	1.000	Enzyme	Ligase
Methylated-DNA--protein-cysteine methyltransferase inhibitor	P16455	31	0.9966	0.997	Enzyme	NA
Methyl-CpG-binding domain protein 2 inhibitor	Q9UBB5	7	0.9779	0.978	Epigenetic regulator	Reader
Methyl-CpG-binding protein 2 inhibitor	P51608	3	0.9191	0.923	Epigenetic regulator	Reader
Microsomal triglyceride transfer protein large subunit inhibitor	P55157	154	1.000	1.000	Other cytosolic protein	NA
Microtubule-associated protein 2 inhibitor	P11137	20	0.9628	0.963	Other cytosolic protein	NA
Microtubule-associated protein tau inhibitor	P10636	75	0.9898	0.99	Other cytosolic protein	NA
Microtubule-associated serine/threonine-protein kinase 3 inhibitor	O60307	8	1.000	1.000	Enzyme	Kinase
Microtubule-associated serine/threonine-protein kinase 4 inhibitor	O15021	4	1.000	1.000	Enzyme	Kinase
Mineralocorticoid receptor antagonist	P08235	709	0.9936	0.993	Transcription factor	Nuclear receptor
Misshapen-like kinase 1 inhibitor	Q8N4C8	22	0.8988	0.898	Enzyme	Kinase
Mitogen-activated protein kinase 1 inhibitor	P28482	3920	0.8309	0.831	Enzyme	Kinase
Mitogen-activated protein kinase 10 inhibitor	P53779	920	0.9824	0.983	Enzyme	Kinase
Mitogen-activated protein kinase 11 inhibitor	Q15759	743	0.9853	0.985	Enzyme	Kinase
Mitogen-activated protein kinase 12 inhibitor	P53778	91	0.9524	0.954	Enzyme	Kinase
Mitogen-activated protein kinase 13 inhibitor	O15264	80	0.9201	0.92	Enzyme	Kinase
Mitogen-activated protein kinase 14 inhibitor	Q16539	3681	0.984	0.984	Enzyme	Kinase
Mitogen-activated protein kinase 3 inhibitor	P27361	244	0.952	0.952	Enzyme	Kinase
Mitogen-activated protein kinase 6 inhibitor	Q16659	3	0.983	0.983	Enzyme	Kinase
Mitogen-activated protein kinase 7 inhibitor	Q13164	85	0.9835	0.983	Enzyme	Kinase
Mitogen-activated protein kinase 8 inhibitor	P45983	1087	0.9862	0.986	Enzyme	Kinase
Mitogen-activated protein kinase 9 inhibitor	P45984	654	0.9809	0.981	Enzyme	Kinase

Mitogen-activated protein kinase kinase kinase 10 inhibitor	Q02779	23	0.9367	0.934	Enzyme	Kinase
Mitogen-activated protein kinase kinase kinase 11 inhibitor	Q16584	47	0.9392	0.939	Enzyme	Kinase
Mitogen-activated protein kinase kinase kinase 12 inhibitor	Q12852	586	0.9988	0.999	Enzyme	Kinase
Mitogen-activated protein kinase kinase kinase 14 inhibitor	Q99558	592	0.9971	0.997	Enzyme	Kinase
Mitogen-activated protein kinase kinase kinase 15 inhibitor	Q6ZN16	3	0.983	0.983	Enzyme	Kinase
Mitogen-activated protein kinase kinase kinase 20 inhibitor	Q9NYL2	53	0.934	0.934	Enzyme	Kinase
Mitogen-activated protein kinase kinase kinase 4 inhibitor	Q9Y6R4	3	0.983	0.983	Enzyme	Kinase
Mitogen-activated protein kinase kinase kinase 5 inhibitor	Q99683	207	0.9824	0.978	Enzyme	Kinase
Mitogen-activated protein kinase kinase kinase 7 inhibitor	O43318	213	0.9815	0.981	Enzyme	Kinase
Mitogen-activated protein kinase kinase kinase 8 inhibitor	P41279	226	0.9916	0.992	Enzyme	Kinase
Mitogen-activated protein kinase kinase kinase 9 inhibitor	P80192	54	0.9468	0.947	Enzyme	Kinase
Mitogen-activated protein kinase kinase kinase kinase 1 inhibitor	Q92918	5	0.9359	0.937	Enzyme	Kinase
Mitogen-activated protein kinase kinase kinase kinase 2 inhibitor	Q12851	51	0.9507	0.949	Enzyme	Kinase
Mitogen-activated protein kinase kinase kinase kinase 4 inhibitor	O95819	113	0.9491	0.95	Enzyme	Kinase
Mitotic checkpoint serine/threonine-protein kinase BUB1 inhibitor	O43683	7	0.874	0.875	Enzyme	Kinase
Monoacylglycerol lipase ABHD6 inhibitor	Q9BV23	74	0.9838	0.984	Enzyme	Hydrolase
Monocarboxylate transporter 1 inhibitor	P53985	58	1.000	1.000	Transporter	Electrochemical transporter
Monocarboxylate transporter 4 inhibitor	O15427	9	1.000	1.000	Transporter	Electrochemical transporter
Monoglyceride lipase inhibitor	Q99685	422	0.9942	0.994	Enzyme	Hydrolase
Motilin receptor agonist	O43193	175	0.9864	0.986	Membrane receptor	Family A G protein-coupled receptor
Motilin receptor antagonist	O43193	27	0.9764	0.977	Membrane receptor	Family A G protein-coupled receptor
M-phase inducer phosphatase 1 inhibitor	P30304	178	0.9875	0.987	Enzyme	Phosphatase
M-phase inducer phosphatase 2 inhibitor	P30305	356	0.9911	0.991	Enzyme	Phosphatase
M-phase inducer phosphatase 3 inhibitor	P30307	54	0.9786	0.979	Enzyme	Phosphatase
Mucosa-associated lymphoid tissue lymphoma translocation protein 1 inhibitor	Q9UDY8	84	0.9974	0.997	Enzyme	Hydrolase
Multidrug resistance-associated protein 1 inhibitor	P33527	263	0.9708	0.965	Transporter	Primary active transporter
Multidrug resistance-associated protein 5 inhibitor	O15440	3	1.000	1.000	Transporter	Primary active transporter
Muscarinic acetylcholine receptor M1 agonist	P11229	283	0.9971	0.997	Membrane receptor	Family A G protein-coupled receptor
Muscarinic acetylcholine receptor M1 antagonist	P11229	807	0.9794	0.98	Membrane receptor	Family A G protein-

						coupled receptor
Muscarinic acetylcholine receptor M2 agonist	P08172	66	0.9931	0.993	Membrane receptor	Family A G protein-coupled receptor
Muscarinic acetylcholine receptor M2 antagonist	P08172	920	0.9835	0.984	Membrane receptor	Family A G protein-coupled receptor
Muscarinic acetylcholine receptor M3 agonist	P20309	34	0.9935	0.994	Membrane receptor	Family A G protein-coupled receptor
Muscarinic acetylcholine receptor M3 antagonist	P20309	829	0.9871	0.987	Membrane receptor	Family A G protein-coupled receptor
Muscarinic acetylcholine receptor M4 agonist	P08173	87	0.9972	0.997	Membrane receptor	Family A G protein-coupled receptor
Muscarinic acetylcholine receptor M4 antagonist	P08173	440	0.9831	0.983	Membrane receptor	Family A G protein-coupled receptor
Muscarinic acetylcholine receptor M5 agonist	P08912	47	0.9849	0.986	Membrane receptor	Family A G protein-coupled receptor
Muscarinic acetylcholine receptor M5 antagonist	P08912	331	0.9778	0.978	Membrane receptor	Family A G protein-coupled receptor
Mu-type opioid receptor agonist	P35372	911	0.9926	0.993	Membrane receptor	Family A G protein-coupled receptor
Mu-type opioid receptor antagonist	P35372	1887	0.9866	0.987	Membrane receptor	Family A G protein-coupled receptor
Myc proto-oncogene protein inhibitor	P01106	407	0.9992	0.999	Transcription factor	NA
Myelin-associated glycoprotein inhibitor	P20916	12	1.000	1.000	Adhesion	NA
Myeloblastin inhibitor	P24158	31	0.9985	0.997	Enzyme	Protease
Myeloperoxidase inhibitor	P05164	281	0.9852	0.985	Enzyme	Oxidoreductase
Myoglobin inhibitor	P02144	4	0.9989	0.999	Unclassified protein	NA
Myosin light chain kinase, smooth muscle inhibitor	Q15746	57	0.9164	0.913	Enzyme	Kinase
N(G),N(G)-dimethylarginine dimethylaminohydrolase 1 inhibitor	O94760	18	0.9607	0.963	Enzyme	Hydrolase
Na(+)/H(+) exchange regulatory cofactor NHE-RF1 inhibitor	O14745	4	1.000	1.000	Unclassified protein	NA
N-acetylated-alpha-linked acidic dipeptidase 2 inhibitor	Q9Y3Q0	21	0.9999	1.000	Enzyme	Protease
NACHT, LRR and PYD domains-containing protein 3 inhibitor	Q96P20	48	0.9904	0.991	Unclassified protein	NA
N-acylethanolamine-hydrolyzing acid amidase inhibitor	Q02083	123	0.9906	0.991	Enzyme	Hydrolase
NAD(+) hydrolase SARM1 inhibitor	Q6SZW1	5	1.000	1.000	Enzyme	Hydrolase
NAD(P)H dehydrogenase [quinone] 1 inhibitor	P15559	130	0.9937	0.994	Enzyme	Oxidoreductase
NAD-dependent protein deacetylase sirtuin-1 inhibitor	Q96EB6	226	0.9753	0.97	Epigenetic regulator	Eraser
NAD-dependent protein deacetylase sirtuin-2 inhibitor	Q8IXJ6	474	0.9912	0.991	Epigenetic regulator	Eraser
NAD-dependent protein deacetylase sirtuin-3, mitochondrial inhibitor	Q9NTG7	139	0.987	0.987	Epigenetic regulator	Eraser

NAD-dependent protein deacetylase sirtuin-6 inhibitor	Q8N6T7	9	0.8447	0.848	Epigenetic regulator	Eraser
NAD-dependent protein deacylase sirtuin-5, mitochondrial inhibitor	Q9NXA8	54	0.9825	0.983	Epigenetic regulator	Eraser
NADH dehydrogenase [ubiquinone] 1 alpha subcomplex subunit 6 inhibitor	P56556	3	0.9886	0.989	Enzyme	Oxidoreductase
NADH-ubiquinone oxidoreductase chain 4 inhibitor	P03905	4	1.000	1.000	Enzyme	Oxidoreductase
NADP-dependent malic enzyme inhibitor	P48163	6	1.000	1.000	Enzyme	NA
NADPH oxidase 1 inhibitor	Q9Y5S8	12	0.9992	0.918	Transporter	Transmembrane 1-electron transfer carriers
NADPH oxidase 4 inhibitor	Q9NPH5	109	0.9927	0.993	Enzyme	NA
N-alpha-acetyltransferase 50 inhibitor	Q9GZZ1	11	0.9161	0.917	Enzyme	Transferase
N-arachidonyl glycine receptor agonist	Q14330	4	0.9211	0.878	Membrane receptor	Family A G protein-coupled receptor
N-arachidonyl glycine receptor antagonist	Q14330	26	0.972	0.972	Membrane receptor	Family A G protein-coupled receptor
Natriuretic peptides A inhibitor	P01160	11	0.999	0.999	Secreted protein	NA
Natural resistance-associated macrophage protein 2 inhibitor	P49281	39	1.000	1.000	Transporter	Electrochemical transporter
NEDD8-activating enzyme E1 catalytic subunit inhibitor	Q8TBC4	47	0.9943	0.994	Enzyme	Ligase
Neprilysin inhibitor	P08473	425	0.9983	0.998	Enzyme	Protease
Neuroendocrine convertase 1 inhibitor	P29120	12	1.000	1.000	Enzyme	Protease
Neuroendocrine convertase 2 inhibitor	P16519	12	1.000	1.000	Enzyme	Protease
Neurogenic locus notch homolog protein 1 inhibitor	P46531	42	0.9997	1.000	Membrane receptor	NA
Neurogenic locus notch homolog protein 3 inhibitor	Q9UM47	20	1.000	1.000	Membrane receptor	NA
Neuromedin-B receptor agonist	P28336	3	0.85	0.853	Membrane receptor	Family A G protein-coupled receptor
Neuromedin-B receptor antagonist	P28336	63	0.9983	0.998	Membrane receptor	Family A G protein-coupled receptor
Neuromedin-K receptor agonist	P29371	62	1.000	1.000	Membrane receptor	Family A G protein-coupled receptor
Neuromedin-K receptor antagonist	P29371	571	0.9964	0.997	Membrane receptor	Family A G protein-coupled receptor
Neuromedin-U receptor 1 agonist	Q9HB89	151	0.9997	1.000	Membrane receptor	Family A G protein-coupled receptor
Neuromedin-U receptor 1 antagonist	Q9HB89	4	0.9999	1.000	Membrane receptor	Family A G protein-coupled receptor
Neuromedin-U receptor 2 agonist	Q9GZQ4	166	0.9957	0.996	Membrane receptor	Family A G protein-coupled receptor
Neuromedin-U receptor 2 antagonist	Q9GZQ4	4	0.9999	1.000	Membrane receptor	Family A G protein-

						coupled receptor
Neuronal acetylcholine receptor subunit alpha-2 antagonist	Q15822	17	0.9705	0.971	Ion channel	Ligand-gated ion channel
Neuronal acetylcholine receptor subunit alpha-3 agonist	P32297	136	0.9963	0.996	Ion channel	Ligand-gated ion channel
Neuronal acetylcholine receptor subunit alpha-3 antagonist	P32297	238	0.9834	0.984	Ion channel	Ligand-gated ion channel
Neuronal acetylcholine receptor subunit alpha-4 agonist	P43681	259	0.9993	0.999	Ion channel	Ligand-gated ion channel
Neuronal acetylcholine receptor subunit alpha-4 antagonist	P43681	416	0.9866	0.984	Ion channel	Ligand-gated ion channel
Neuronal acetylcholine receptor subunit alpha-6 antagonist	Q15825	6	1.000	1.000	Ion channel	Ligand-gated ion channel
Neuronal acetylcholine receptor subunit alpha-7 agonist	P36544	225	0.9974	0.998	Ion channel	Ligand-gated ion channel
Neuronal acetylcholine receptor subunit alpha-7 antagonist	P36544	155	0.9721	0.972	Ion channel	Ligand-gated ion channel
Neuronal acetylcholine receptor subunit alpha-9 antagonist	Q9UGM1	35	0.9988	0.999	Ion channel	Ligand-gated ion channel
Neuronal acetylcholine receptor subunit beta-4 antagonist	P30926	50	0.9962	0.997	Ion channel	Ligand-gated ion channel
Neuropeptide FF receptor 1 agonist	Q9GZQ6	26	0.9993	0.999	Membrane receptor	Family A G protein-coupled receptor
Neuropeptide FF receptor 1 antagonist	Q9GZQ6	10	0.9987	0.999	Membrane receptor	Family A G protein-coupled receptor
Neuropeptide FF receptor 2 agonist	Q9Y5X5	35	0.9998	1.000	Membrane receptor	Family A G protein-coupled receptor
Neuropeptide FF receptor 2 antagonist	Q9Y5X5	7	0.9979	0.998	Membrane receptor	Family A G protein-coupled receptor
Neuropeptide S receptor antagonist	Q6W5P4	7063	0.8783	0.878	Membrane receptor	Family A G protein-coupled receptor
Neuropeptide Y receptor type 1 agonist	P25929	12	0.812	0.812	Membrane receptor	Family A G protein-coupled receptor
Neuropeptide Y receptor type 1 antagonist	P25929	292	0.9831	0.983	Membrane receptor	Family A G protein-coupled receptor
Neuropeptide Y receptor type 2 agonist	P49146	82	0.9996	1.000	Membrane receptor	Family A G protein-coupled receptor
Neuropeptide Y receptor type 2 antagonist	P49146	238	0.9232	0.925	Membrane receptor	Family A G protein-coupled receptor
Neuropeptide Y receptor type 4 agonist	P50391	65	0.9991	0.999	Membrane receptor	Family A G protein-coupled receptor
Neuropeptide Y receptor type 4 antagonist	P50391	30	0.9998	1.000	Membrane receptor	Family A G protein-coupled receptor

Neuropeptide Y receptor type 5 agonist	Q15761	6	0.9989	0.999	Membrane receptor	Family A G protein-coupled receptor
Neuropeptide Y receptor type 5 antagonist	Q15761	776	0.993	0.993	Membrane receptor	Family A G protein-coupled receptor
Neuropeptides B/W receptor type 1 antagonist	P48145	169	0.9871	0.987	Membrane receptor	Family A G protein-coupled receptor
Neuropilin-1 inhibitor	O14786	37	0.9811	0.981	Membrane receptor; Secreted protein	NA
Neurotensin receptor type 1 agonist	P30989	92	0.9957	0.996	Membrane receptor	Family A G protein-coupled receptor
Neurotensin receptor type 1 antagonist	P30989	69	0.9908	0.988	Membrane receptor	Family A G protein-coupled receptor
Neurotensin receptor type 2 agonist	O95665	6	0.9999	1.000	Membrane receptor	Family A G protein-coupled receptor
Neurotensin receptor type 2 antagonist	O95665	27	0.9743	0.975	Membrane receptor	Family A G protein-coupled receptor
Neurotrypsin inhibitor	P56730	114	1.000	1.000	Enzyme	Hydrolase
Neutral alpha-glucosidase AB inhibitor	Q14697	4	0.9999	1.000	Enzyme	Hydrolase
Neutral amino acid transporter B(0) inhibitor	Q15758	4	0.874	0.876	Transporter	Electrochemical transporter
Neutral cholesterol ester hydrolase 1 inhibitor	Q6PIU2	27	0.9971	0.997	Enzyme	NA
Neutrophil collagenase inhibitor	P22894	912	0.9937	0.994	Enzyme	Protease
Neutrophil elastase inhibitor	P08246	1494	0.9951	0.995	Enzyme	Protease
N-formyl peptide receptor 2 agonist	P25090	465	0.9967	0.996	Membrane receptor	Family A G protein-coupled receptor
N-formyl peptide receptor 2 antagonist	P25090	14	0.9525	0.954	Membrane receptor	Family A G protein-coupled receptor
N-glycosylase/DNA lyase inhibitor	O15527	15	0.9692	0.97	Enzyme	NA
Nicotinamide N-methyltransferase inhibitor	P40261	57	0.9996	1.000	Enzyme	Transferase
Nicotinamide phosphoribosyltransferase inhibitor	P43490	816	0.9982	0.998	Enzyme	NA
Nicotinate phosphoribosyltransferase inhibitor	Q6XQN6	22	0.9942	0.994	Enzyme	Ligase
Nischarin inhibitor	Q9Y2I1	139	0.999	0.999	Other cytosolic protein	NA
Nitric oxide synthase, brain inhibitor	P29475	830	0.9987	0.999	Enzyme	NA
Nitric oxide synthase, endothelial inhibitor	P29474	335	0.998	0.998	Enzyme	NA
Nitric oxide synthase, inducible inhibitor	P35228	648	0.9957	0.996	Enzyme	NA
N-lysine methyltransferase KMT5A inhibitor	Q9NQR1	13	0.8561	0.859	Epigenetic regulator	Writer
N-lysine methyltransferase SMYD2 inhibitor	Q9NRG4	34	0.9929	0.993	Epigenetic regulator	Writer
Nociceptin receptor agonist	P41146	213	0.9991	0.999	Membrane receptor	Family A G protein-

						coupled receptor
Nociceptin receptor antagonist	P41146	504	0.9949	0.995	Membrane receptor	Family A G protein-coupled receptor
Non-lysosomal glucosylceramidase inhibitor	Q9HCG7	57	1.000	1.000	Enzyme	NA
Non-receptor tyrosine-protein kinase TYK2 inhibitor	P29597	869	0.9896	0.99	Enzyme	Kinase
NPC1-like intracellular cholesterol transporter 1 inhibitor	Q9UHC9	31	1.000	1.000	Other membrane protein	NA
NT-3 growth factor receptor agonist	Q16288	11	1.000	1.000	Enzyme	Kinase
NT-3 growth factor receptor antagonist	Q16288	88	0.9401	0.938	Enzyme	Kinase
N-terminal Xaa-Pro-Lys N-methyltransferase 1 inhibitor	Q9BV86	18	1.000	1.000	Enzyme	Transferase
NUAK family SNF1-like kinase 1 inhibitor	O60285	37	0.9495	0.947	Enzyme	Kinase
Nuclear factor NF-kappa-B p100 subunit inhibitor	Q00653	165	0.9865	0.986	Transcription factor; Other cytosolic protein	NA
Nuclear factor NF-kappa-B p105 subunit inhibitor	P19838	38	0.994	0.994	Transcription factor; Other cytosolic protein	NA
Nuclear receptor corepressor 1 antagonist	O75376	60	0.9977	0.998	Epigenetic regulator	Writer
Nuclear receptor corepressor 2 antagonist	Q9Y618	280	0.998	0.998	Unclassified protein	NA
Nuclear receptor ROR-alpha agonist	P35398	64	0.9915	0.992	Transcription factor	Nuclear receptor
Nuclear receptor ROR-alpha antagonist	P35398	16	0.9996	1.000	Transcription factor	Nuclear receptor
Nuclear receptor ROR-beta agonist	Q92753	71	0.9944	0.994	Transcription factor	Nuclear receptor
Nuclear receptor ROR-beta antagonist	Q92753	14	0.998	0.998	Transcription factor	Nuclear receptor
Nuclear receptor ROR-gamma agonist	P51449	1163	0.9956	0.996	Transcription factor	Nuclear receptor
Nuclear receptor ROR-gamma antagonist	P51449	847	0.9928	0.993	Transcription factor	Nuclear receptor
Nuclear receptor subfamily 1 group D member 1 agonist	P20393	68	1.000	1.000	Transcription factor	Nuclear receptor
Nuclear receptor subfamily 1 group I member 2 agonist	O75469	95	0.9745	0.976	Transcription factor	Nuclear receptor
Nuclear receptor subfamily 1 group I member 2 antagonist	O75469	62	0.8906	0.887	Transcription factor	Nuclear receptor
Nuclear receptor subfamily 1 group I member 3 agonist	Q14994	101	0.9954	0.996	Transcription factor	Nuclear receptor
Nuclear receptor subfamily 4 group A member 2 agonist	P43354	12	0.9995	1.000	Transcription factor	Nuclear receptor
Nuclear receptor subfamily 5 group A member 2 agonist	O00482	40	1.000	1.000	Transcription factor	Nuclear receptor
Nuclear receptor subfamily 5 group A member 2 antagonist	O00482	27	0.9982	0.998	Transcription factor	Nuclear receptor
Nucleotide-binding oligomerization domain-containing protein 1 inhibitor	Q9Y239	44	0.9992	0.999	Unclassified protein	NA
Nucleotide-binding oligomerization domain-containing protein 2 inhibitor	Q9HC29	31	0.958	0.958	Unclassified protein	NA
Olfactory receptor 51E2 agonist	Q9H255	21	0.9748	0.976	Membrane receptor	Family A G protein-coupled receptor
Opioid growth factor receptor-like protein 1 agonist	Q5TC84	25	0.9999	1.000	Membrane receptor	NA

Opioid growth factor receptor-like protein 1 antagonist	Q5TC84	357	0.9977	0.998	Membrane receptor	NA
Orexin receptor type 1 agonist	O43613	61	0.9977	0.998	Membrane receptor	Family A G protein-coupled receptor
Orexin receptor type 1 antagonist	O43613	2440	0.9959	0.996	Membrane receptor	Family A G protein-coupled receptor
Orexin receptor type 2 agonist	O43614	75	0.9977	0.998	Membrane receptor	Family A G protein-coupled receptor
Orexin receptor type 2 antagonist	O43614	2636	0.996	0.996	Membrane receptor	Family A G protein-coupled receptor
Ornithine decarboxylase inhibitor	P11926	10	0.9996	1.000	Enzyme	Lyase
Oxidized purine nucleoside triphosphate hydrolase inhibitor	P36639	102	0.9945	0.995	Enzyme	Hydrolase
Oxoeicosanoid receptor 1 agonist	Q8TDS5	5	1.000	1.000	Membrane receptor	Family A G protein-coupled receptor
Oxoeicosanoid receptor 1 antagonist	Q8TDS5	43	1.000	1.000	Membrane receptor	Family A G protein-coupled receptor
Oxysterols receptor LXR-alpha agonist	Q13133	280	0.9933	0.993	Transcription factor	Nuclear receptor
Oxysterols receptor LXR-alpha antagonist	Q13133	314	0.9961	0.996	Transcription factor	Nuclear receptor
Oxysterols receptor LXR-beta agonist	P55055	448	0.9932	0.993	Transcription factor	Nuclear receptor
Oxysterols receptor LXR-beta antagonist	P55055	345	0.9947	0.995	Transcription factor	Nuclear receptor
Oxytocin receptor agonist	P30559	220	0.9949	0.995	Membrane receptor	Family A G protein-coupled receptor
Oxytocin receptor antagonist	P30559	425	0.998	0.998	Membrane receptor	Family A G protein-coupled receptor
P2Y purinoceptor 1 agonist	P47900	23	0.9997	1.000	Membrane receptor	Family A G protein-coupled receptor
P2Y purinoceptor 1 antagonist	P47900	114	0.9997	1.000	Membrane receptor	Family A G protein-coupled receptor
P2Y purinoceptor 11 agonist	Q96G91	15	1.000	1.000	Membrane receptor	Family A G protein-coupled receptor
P2Y purinoceptor 12 antagonist	Q9H244	328	0.9987	0.999	Membrane receptor	Family A G protein-coupled receptor
P2Y purinoceptor 14 agonist	Q15391	21	1.000	1.000	Membrane receptor	Family A G protein-coupled receptor
P2Y purinoceptor 14 antagonist	Q15391	8	1.000	1.000	Membrane receptor	Family A G protein-coupled receptor
P2Y purinoceptor 2 agonist	P41231	181	0.9999	1.000	Membrane receptor	Family A G protein-coupled receptor
P2Y purinoceptor 2 antagonist	P41231	17	1.000	1.000	Membrane receptor	Family A G protein-coupled receptor
P2Y purinoceptor 4 agonist	P51582	91	0.9999	1.000	Membrane receptor	Family A G protein-coupled receptor

P2Y purinoceptor 6 agonist	Q15077	107	0.9999	1.000	Membrane receptor	Family A G protein-coupled receptor
Palmitoleoyl-protein carboxylesterase NOTUM inhibitor	Q6P988	199	0.9994	0.999	Enzyme	Hydrolase
Pancreatic alpha-amylase inhibitor	P04746	12	1.000	1.000	Enzyme	Hydrolase
Pancreatic triacylglycerol lipase inhibitor	P16233	14	0.9828	0.984	Enzyme	Hydrolase
Pantothenate kinase 1 inhibitor	Q8TE04	11	1.000	1.000	Enzyme	Transferase
Pantothenate kinase 3 inhibitor	Q9H999	10	0.9217	0.921	Enzyme	Transferase
Parathyroid hormone/parathyroid hormone-related peptide receptor agonist	Q03431	49	0.9998	1.000	Membrane receptor	Family B G protein-coupled receptor
Parathyroid hormone/parathyroid hormone-related peptide receptor antagonist	Q03431	25	0.9837	0.984	Membrane receptor	Family B G protein-coupled receptor
PC4 and SFRS1-interacting protein inhibitor	O75475	3	1.000	1.000	Epigenetic regulator	Reader
Pepsin A-5 inhibitor	P0DJD9	23	0.9926	0.993	Enzyme	Protease
Peptide deformylase, mitochondrial inhibitor	Q9HBH1	64	0.9999	1.000	Enzyme	Hydrolase
Peptidyl-glycine alpha-amidating monooxygenase inhibitor	P19021	9	0.9996	1.000	Enzyme	NA
Peptidyl-prolyl cis-trans isomerase A inhibitor	P62937	180	0.9984	0.998	Enzyme	Isomerase
Peptidyl-prolyl cis-trans isomerase B inhibitor	P23284	21	1.000	1.000	Enzyme	Isomerase
Peptidyl-prolyl cis-trans isomerase D inhibitor	Q08752	13	0.9531	0.951	Enzyme	Isomerase
Peptidyl-prolyl cis-trans isomerase FKBP1A inhibitor	P62942	436	0.9999	1.000	Enzyme	Isomerase
Peptidyl-prolyl cis-trans isomerase FKBP1B inhibitor	P68106	6	0.8607	0.862	Enzyme	Isomerase
Peptidyl-prolyl cis-trans isomerase FKBP4 inhibitor	Q02790	16	0.9732	0.974	Enzyme	Isomerase
Peptidyl-prolyl cis-trans isomerase FKBP5 inhibitor	Q13451	71	0.9999	1.000	Enzyme	NA
Peptidyl-prolyl cis-trans isomerase NIMA-interacting 1 inhibitor	Q13526	176	0.9917	0.992	Enzyme	Isomerase
Peregrin inhibitor	P55201	156	0.9879	0.988	Epigenetic regulator	Reader
Perforin-1 inhibitor	P14222	211	1.000	1.000	Ion channel	Other ion channel
Peripheral plasma membrane protein CASK inhibitor	O14936	3	0.983	0.983	Enzyme	NA
Peroxisomal membrane protein PEX14 inhibitor	O75381	8	1.000	1.000	Unclassified protein	NA
Peroxisomal N(1)-acetyl-spermine/spermidine oxidase inhibitor	Q6QHF9	9	1.000	1.000	Enzyme	NA
Peroxisome proliferator-activated receptor alpha agonist	Q07869	1326	0.9966	0.997	Transcription factor	Nuclear receptor
Peroxisome proliferator-activated receptor alpha antagonist	Q07869	659	0.9985	0.999	Transcription factor	Nuclear receptor
Peroxisome proliferator-activated receptor delta agonist	Q03181	882	0.9978	0.998	Transcription factor	Nuclear receptor
Peroxisome proliferator-activated receptor delta antagonist	Q03181	250	0.9907	0.991	Transcription factor	Nuclear receptor
Peroxisome proliferator-activated receptor gamma agonist	P37231	1703	0.9935	0.994	Transcription factor	Nuclear receptor
Peroxisome proliferator-activated receptor gamma antagonist	P37231	1238	0.9909	0.991	Transcription factor	Nuclear receptor

PH and SEC7 domain-containing protein 1 inhibitor	A5PKW4	112	0.9785	0.979	Unclassified protein	NA
PH domain leucine-rich repeat-containing protein phosphatase 2 inhibitor	Q6ZVD8	12	0.9521	0.952	Enzyme	Phosphatase
Phenylalanine--tRNA ligase, mitochondrial inhibitor	O95363	3	1.000	1.000	Enzyme	Ligase
Phenylethanolamine N-methyltransferase inhibitor	P11086	110	0.9996	1.000	Enzyme	Transferase
Phosphatidylcholine transfer protein inhibitor	Q9UKL6	11	1.000	1.000	Unclassified protein	NA
Phosphatidylcholine:ceramide cholinephosphotransferase 1 inhibitor	Q86VZ5	16	0.9995	0.999	Enzyme	Transferase
Phosphatidylcholine:ceramide cholinephosphotransferase 2 inhibitor	Q8NHU3	82	0.9996	0.999	Enzyme	Transferase
Phosphatidylinositol 3,4,5-trisphosphate 5-phosphatase 2 inhibitor	O15357	11	0.9959	0.996	Enzyme	NA
Phosphatidylinositol 3-kinase catalytic subunit type 3 inhibitor	Q8NEB9	75	0.9464	0.948	Enzyme	Transferase
Phosphatidylinositol 3-kinase regulatory subunit alpha inhibitor	P27986	1052	0.9931	0.993	Enzyme	NA
Phosphatidylinositol 3-kinase regulatory subunit beta inhibitor	O00459	377	0.9979	0.998	Enzyme	NA
Phosphatidylinositol 4,5-bisphosphate 3-kinase catalytic subunit alpha isoform inhibitor	P42336	6064	0.9907	0.991	Enzyme	Transferase
Phosphatidylinositol 4,5-bisphosphate 3-kinase catalytic subunit beta isoform inhibitor	P42338	1632	0.989	0.989	Enzyme	Transferase
Phosphatidylinositol 4,5-bisphosphate 3-kinase catalytic subunit delta isoform inhibitor	O00329	2351	0.989	0.989	Enzyme	Transferase
Phosphatidylinositol 4,5-bisphosphate 3-kinase catalytic subunit gamma isoform inhibitor	P48736	2505	0.9812	0.981	Enzyme	Transferase
Phosphatidylinositol 4-kinase alpha inhibitor	P42356	84	0.9981	0.998	Enzyme	Transferase
Phosphatidylinositol 4-kinase beta inhibitor	Q9UBF8	247	0.9857	0.985	Enzyme	Transferase
Phosphatidylinositol 4-phosphate 3-kinase C2 domain-containing subunit alpha inhibitor	O00443	15	0.9418	0.943	Enzyme	Transferase
Phosphatidylinositol 4-phosphate 3-kinase C2 domain-containing subunit beta inhibitor	O00750	45	0.9611	0.961	Enzyme	Transferase
Phosphatidylinositol 4-phosphate 3-kinase C2 domain-containing subunit gamma inhibitor	O75747	7	0.8979	0.902	Enzyme	Transferase
Phosphatidylinositol 4-phosphate 5-kinase type-1 gamma inhibitor	O60331	6	0.9546	0.959	Enzyme	Transferase
Phosphatidylinositol 5-phosphate 4-kinase type-2 alpha inhibitor	P48426	32	0.9727	0.973	Enzyme	Transferase
Phosphatidylinositol 5-phosphate 4-kinase type-2 beta inhibitor	P78356	13	0.9904	0.991	Enzyme	Kinase
Phosphatidylinositol 5-phosphate 4-kinase type-2 gamma inhibitor	Q8TBX8	51	0.9873	0.987	Enzyme	Transferase
Phosphatidylserine lipase ABHD16A inhibitor	O95870	6	0.9854	0.986	Enzyme	Hydrolase
Phosphoenolpyruvate carboxykinase, cytosolic [GTP] inhibitor	P35558	21	1.000	1.000	Enzyme	Lyase
Phosphoethanolamine/phosphocholine phosphatase inhibitor	Q8TCT1	53	0.9985	0.999	Enzyme	Phosphatase
Phosphoglycerate kinase 1 inhibitor	P00558	104	1.000	1.000	Enzyme	Transferase
Phosphoglycerate mutase 1 inhibitor	P18669	22	0.9982	0.998	Enzyme	NA
Phospholipase A and acyltransferase 2 inhibitor	Q9NWW9	33	1.000	1.000	Enzyme	Transferase
Phospholipase A and acyltransferase 3 inhibitor	P53816	33	1.000	1.000	Enzyme	Hydrolase
Phospholipase A and acyltransferase 4 inhibitor	Q9UL19	35	1.000	1.000	Enzyme	Transferase

Phospholipase A and acyltransferase 5 inhibitor	Q96KN8	35	1.000	1.000	Enzyme	Transferase
Phospholipase A2 group V inhibitor	P39877	35	0.9998	1.000	Enzyme	Hydrolase
Phospholipase A2 inhibitor	P04054	257	0.9148	0.914	Enzyme	Hydrolase
Phospholipase A2, membrane associated inhibitor	P14555	313	0.9987	0.999	Enzyme	Hydrolase
Phospholipase A-2-activating protein inhibitor	Q9Y263	17	0.9997	1.000	Unclassified protein	NA
Phospholipase D1 inhibitor	Q13393	130	0.9947	0.995	Enzyme	Hydrolase
Phospholipase D2 inhibitor	O14939	113	0.9948	0.995	Enzyme	Hydrolase
Phosphomannomutase 2 inhibitor	O15305	13	0.996	0.996	Enzyme	Isomerase
Phosphorylase b kinase gamma catalytic chain, liver/testis isoform inhibitor	P15735	9	0.9595	0.962	Enzyme	Kinase
Phosphorylase b kinase regulatory subunit alpha, skeletal muscle isoform inhibitor	P46020	3	0.9992	0.999	Enzyme	Kinase
Photoreceptor-specific nuclear receptor agonist	Q9Y5X4	17	1.000	1.000	Transcription factor	Nuclear receptor
Pituitary adenylate cyclase-activating polypeptide type I receptor agonist	P41586	44	1.000	1.000	Unclassified protein	NA
Placenta growth factor inhibitor	P49763	16	1.000	1.000	Secreted protein	NA
Plasma kallikrein inhibitor	P03952	833	0.9935	0.994	Enzyme	Protease
Plasminogen activator inhibitor 1 inhibitor	P05121	185	0.9983	0.998	Secreted protein	NA
Plasminogen inhibitor	P00747	749	0.9902	0.99	Enzyme	Protease
Platelet-activating factor acetylhydrolase IB subunit alpha2 inhibitor	P68402	4	0.959	0.959	Enzyme	NA
Platelet-activating factor acetylhydrolase IB subunit beta inhibitor	P68402	8	0.9714	0.971	Enzyme	NA
Platelet-activating factor acetylhydrolase inhibitor	Q13093	444	0.9961	0.996	Enzyme	Hydrolase
Platelet-activating factor receptor antagonist	P25105	310	0.9941	0.994	Membrane receptor	Family A G protein-coupled receptor
Platelet-derived growth factor receptor alpha antagonist	P16234	787	0.9775	0.977	Enzyme	Kinase
Platelet-derived growth factor receptor beta antagonist	P09619	1147	0.9776	0.978	Enzyme	Kinase
Poly [ADP-ribose] polymerase 1 inhibitor	P09874	3101	0.9951	0.995	Enzyme	Transferase
Poly [ADP-ribose] polymerase 2 inhibitor	Q9UGN5	241	0.9968	0.997	Enzyme	Transferase
Poly [ADP-ribose] polymerase tankyrase-1 inhibitor	O95271	733	0.9939	0.994	Enzyme	Transferase
Poly [ADP-ribose] polymerase tankyrase-2 inhibitor	Q9H2K2	586	0.9949	0.995	Enzyme	Transferase
Poly(ADP-ribose) glycohydrolase activator	Q86W56	41	1.000	1.000	Enzyme	NA
Poly(ADP-ribose) glycohydrolase inhibitor	Q86W56	95	0.9812	0.981	Enzyme	NA
Polyamine deacetylase HDAC10 inhibitor	Q969S8	260	0.9972	0.997	Epigenetic regulator	Eraser
Polycomb protein EED inhibitor	O75530	203	0.9968	0.994	Unclassified protein	NA
Polypeptide N-acetylgalactosaminyltransferase 2 inhibitor	Q10471	3	0.9998	1.000	Enzyme	Transferase
Polyunsaturated fatty acid 5-lipoxygenase inhibitor	P09917	1621	0.9831	0.983	Enzyme	Oxidoreductase

Polyunsaturated fatty acid lipoxygenase ALOX12 inhibitor	P18054	714	0.9039	0.903	Enzyme	NA
Polyunsaturated fatty acid lipoxygenase ALOX15 inhibitor	P16050	1855	0.8783	0.878	Enzyme	Oxidoreductase
Polyunsaturated fatty acid lipoxygenase ALOX15B inhibitor	O15296	6	0.9276	0.929	Enzyme	Oxidoreductase
Potassium channel subfamily K member 2 blocker	O95069	19	0.8565	0.846	Ion channel	Voltage-gated ion channel
Potassium channel subfamily K member 3 blocker	O14649	476	0.9959	0.996	Ion channel	Voltage-gated ion channel
Potassium channel subfamily K member 9 blocker	Q9NPC2	42	0.9582	0.958	Ion channel	Voltage-gated ion channel
Potassium voltage-gated channel subfamily A member 1 blocker	Q09470	53	0.9843	0.985	Ion channel	Voltage-gated ion channel
Potassium voltage-gated channel subfamily A member 2 blocker	P16389	5	0.9713	0.972	Ion channel	Voltage-gated ion channel
Potassium voltage-gated channel subfamily A member 3 blocker	P22001	383	0.9959	0.996	Ion channel	Voltage-gated ion channel
Potassium voltage-gated channel subfamily A member 4 blocker	P22459	10	0.9999	1.000	Ion channel	Voltage-gated ion channel
Potassium voltage-gated channel subfamily A member 5 blocker	P22460	674	0.9954	0.995	Ion channel	Voltage-gated ion channel
Potassium voltage-gated channel subfamily A member 6 blocker	P17658	5	0.9154	0.917	Ion channel	Voltage-gated ion channel
Potassium voltage-gated channel subfamily H member 1 blocker	O95259	5	1.000	1.000	Ion channel	Voltage-gated ion channel
Potassium voltage-gated channel subfamily H member 2 blocker	Q12809	5483	0.9399	0.94	Ion channel	Voltage-gated ion channel
Potassium voltage-gated channel subfamily H member 3 blocker	Q9ULD8	67	1.000	1.000	Ion channel	Voltage-gated ion channel
Potassium voltage-gated channel subfamily KQT member 1 blocker	P51787	50	0.9777	0.978	Ion channel	Voltage-gated ion channel
Potassium voltage-gated channel subfamily KQT member 2 blocker	O43526	210	0.9984	0.998	Ion channel	Voltage-gated ion channel
Potassium voltage-gated channel subfamily KQT member 4 blocker	P56696	14	0.981	0.983	Ion channel	Voltage-gated ion channel
Potassium voltage-gated channel subfamily KQT member 5 blocker	Q9NR82	15	0.9909	0.992	Ion channel	Voltage-gated ion channel
Potassium/sodium hyperpolarization-activated cyclic nucleotide-gated channel 1 blocker	O60741	17	0.9916	0.992	Ion channel	Voltage-gated ion channel
Potassium/sodium hyperpolarization-activated cyclic nucleotide-gated channel 4 blocker	Q9Y3Q4	19	0.9917	0.992	Ion channel	Voltage-gated ion channel
Potassium-transporting ATPase alpha chain 1 inhibitor	P20648	184	0.9987	0.996	Transporter; Enzyme	Primary active transporter; Hydrolase

Potassium-transporting ATPase alpha chain 2 inhibitor	P54707	10	1.000	1.000	Transporter; Enzyme	Primary active transporter; Hydrolase
Presenilin-1 inhibitor	P49768	87	0.9995	1.000	Ion channel	Other ion channel
Presenilin-2 inhibitor	P49810	7	1.000	1.000	Ion channel	Other ion channel
Probable global transcription activator SNF2L2 inhibitor	P51531	15	0.9413	0.939	Epigenetic regulator	Reader
Probable G-protein coupled receptor 139 agonist	Q6DWJ6	22	0.9986	0.999	Membrane receptor	Family A G protein-coupled receptor
Probable G-protein coupled receptor 142 agonist	Q7Z601	146	1.000	1.000	Membrane receptor	Family A G protein-coupled receptor
Probable G-protein coupled receptor 174 agonist	Q9BXC1	13	1.000	1.000	Membrane receptor	Family A G protein-coupled receptor
Probable G-protein coupled receptor 34 agonist	Q9UPC5	16	1.000	1.000	Membrane receptor	Family A G protein-coupled receptor
Probable G-protein coupled receptor 88 agonist	Q9GZN0	135	1.000	1.000	Membrane receptor	Family A G protein-coupled receptor
Probable G-protein coupled receptor 88 antagonist	Q9GZN0	70	1.000	1.000	Membrane receptor	Family A G protein-coupled receptor
Probable maltase-glucoamylase 2 inhibitor	Q2M2H8	346	0.993	0.993	Unclassified protein	NA
Probable ubiquitin carboxyl-terminal hydrolase FAF-X inhibitor	Q93008	10	1.000	1.000	Enzyme	Protease
Pro-cathepsin H inhibitor	P09668	26	0.9679	0.948	Enzyme	Protease
Procathepsin L inhibitor	P07711	1414	0.9925	0.993	Enzyme	Protease
Progesterone receptor agonist	P06401	338	0.9995	1.000	Transcription factor	Nuclear receptor
Progesterone receptor antagonist	P06401	1444	0.9955	0.995	Transcription factor	Nuclear receptor
Programmed cell death 1 ligand 1 inhibitor	Q9NZQ7	15	0.9425	0.943	Unclassified protein	NA
Programmed cell death protein 1 inhibitor	Q15116	73	0.9985	0.999	Surface antigen	NA
Programmed cell death protein 4 inhibitor	Q53EL6	7	0.9892	0.989	Unclassified protein	NA
Prokineticin receptor 1 antagonist	Q8TCW9	177	0.9969	0.996	Membrane receptor	Family A G protein-coupled receptor
Prolactin-releasing peptide receptor antagonist	P49683	95	0.9896	0.99	Membrane receptor	Family A G protein-coupled receptor
Proliferating cell nuclear antigen inhibitor	P12004	20	1.000	1.000	Other nuclear protein	NA
Proline-rich AKT1 substrate 1 inhibitor	Q96B36	3	0.9999	1.000	Unclassified protein	NA
Prolyl 3-hydroxylase OGFOD1 inhibitor	Q8N543	5	0.9994	1.000	Enzyme	Oxidoreductase
Prolyl 4-hydroxylase subunit alpha-1 inhibitor	P13674	3	1.000	1.000	Enzyme	Oxidoreductase
Prolyl endopeptidase FAP inhibitor	Q12884	168	0.9995	1.000	Enzyme	Protease
Prolyl endopeptidase inhibitor	P48147	296	0.9965	0.997	Enzyme	Protease

Prolyl hydroxylase EGLN2 inhibitor	Q96KS0	149	0.999	0.999	Enzyme	Oxidoreductase
Prolyl hydroxylase EGLN3 inhibitor	Q9H6Z9	108	0.9992	0.999	Enzyme	Oxidoreductase
Proprotein convertase subtilisin/kexin type 4 inhibitor	Q6UW60	6	1.000	1.000	Enzyme	Protease
Proprotein convertase subtilisin/kexin type 5 inhibitor	Q92824	18	0.9999	1.000	Enzyme	Protease
Proprotein convertase subtilisin/kexin type 6 inhibitor	P29122	121	0.9999	1.000	Enzyme	Protease
Proprotein convertase subtilisin/kexin type 7 inhibitor	Q16549	20	0.9756	0.975	Enzyme	Protease
Proprotein convertase subtilisin/kexin type 9 inhibitor	Q8NBP7	41	0.9584	0.959	Enzyme	Protease
Prostacyclin receptor agonist	P43119	147	0.9999	1.000	Membrane receptor	Family A G protein-coupled receptor
Prostacyclin receptor antagonist	P43119	129	0.9862	0.986	Membrane receptor	Family A G protein-coupled receptor
Prostacyclin synthase inhibitor	Q16647	16	1.000	1.000	Enzyme	Cytochrome P450
Prostaglandin D2 receptor 2 antagonist	Q9Y5Y4	1970	0.9992	0.999	Membrane receptor	Family A G protein-coupled receptor
Prostaglandin D2 receptor agonist	Q13258	46	0.991	0.991	Membrane receptor	Family A G protein-coupled receptor
Prostaglandin D2 receptor antagonist	Q13258	365	0.9975	0.997	Membrane receptor	Family A G protein-coupled receptor
Prostaglandin E synthase 2 inhibitor	Q9H7Z7	28	0.9938	0.994	Enzyme	NA
Prostaglandin E synthase inhibitor	O14684	1290	0.9875	0.988	Enzyme	Isomerase
Prostaglandin E2 receptor EP1 subtype agonist	P34995	17	0.9942	0.995	Membrane receptor	Family A G protein-coupled receptor
Prostaglandin E2 receptor EP1 subtype antagonist	P34995	405	0.9991	0.999	Membrane receptor	Family A G protein-coupled receptor
Prostaglandin E2 receptor EP2 subtype agonist	P43116	232	0.9987	0.999	Membrane receptor	Family A G protein-coupled receptor
Prostaglandin E2 receptor EP2 subtype antagonist	P43116	181	0.9942	0.993	Membrane receptor	Family A G protein-coupled receptor
Prostaglandin E2 receptor EP3 subtype agonist	P43115	64	0.9999	1.000	Membrane receptor	Family A G protein-coupled receptor
Prostaglandin E2 receptor EP3 subtype antagonist	P43115	262	0.9951	0.995	Membrane receptor	Family A G protein-coupled receptor
Prostaglandin E2 receptor EP4 subtype agonist	P35408	92	0.9999	1.000	Membrane receptor	Family A G protein-coupled receptor
Prostaglandin E2 receptor EP4 subtype antagonist	P35408	469	0.9976	0.998	Membrane receptor	Family A G protein-coupled receptor
Prostaglandin F2-alpha receptor agonist	P43088	21	1.000	1.000	Membrane receptor	Family A G protein-coupled receptor
Prostaglandin F2-alpha receptor antagonist	P43088	35	0.9857	0.986	Membrane receptor	Family A G protein-coupled receptor

Prostaglandin G/H synthase 1 inhibitor	P23219	863	0.9833	0.983	Enzyme	Oxidoreductase
Prostaglandin G/H synthase 2 inhibitor	P35354	2868	0.9809	0.981	Enzyme	Oxidoreductase
Prostasin inhibitor	Q16651	21	1.000	1.000	Enzyme	Protease
Prostate-specific antigen inhibitor	P07288	44	0.9821	0.982	Enzyme	Protease
Prostatic acid phosphatase inhibitor	P15309	9	1.000	1.000	Enzyme	Phosphatase
Proteasome subunit beta type-1 inhibitor	P20618	109	0.9868	0.987	Enzyme	Protease
Proteasome subunit beta type-10 inhibitor	P40306	65	0.999	0.999	Enzyme	Protease
Proteasome subunit beta type-2 inhibitor	P49721	167	0.9875	0.988	Enzyme	Protease
Proteasome subunit beta type-5 inhibitor	P28074	658	0.9913	0.991	Enzyme	Protease
Proteasome subunit beta type-8 inhibitor	P28062	240	0.9986	0.999	Enzyme	Protease
Proteasome subunit beta type-9 inhibitor	P28065	81	0.9997	1.000	Enzyme	Protease
Protein ABHD11 inhibitor	Q8NFV4	3	1.000	1.000	Enzyme	Hydrolase
Protein arginine N-methyltransferase 1 inhibitor	Q99873	66	0.9723	0.973	Epigenetic regulator	Writer
Protein arginine N-methyltransferase 3 inhibitor	O60678	63	0.9957	0.996	Epigenetic regulator	Writer
Protein arginine N-methyltransferase 5 inhibitor	O14744	585	0.9958	0.996	Epigenetic regulator	Writer
Protein arginine N-methyltransferase 6 inhibitor	Q96LA8	95	0.9999	1.000	Epigenetic regulator	Writer
Protein arginine N-methyltransferase 8 inhibitor	Q9NR22	34	0.9997	1.000	Epigenetic regulator	Writer
Protein cereblon inhibitor	Q96SW2	9	1.000	1.000	Unclassified protein	NA
Protein DBF4 homolog A inhibitor	Q9UBU7	190	0.9974	0.997	Enzyme	Kinase
Protein delta homolog 1 inhibitor	P80370	10	0.8897	0.892	Unclassified protein	NA
Protein disulfide-isomerase inhibitor	P07237	52	0.9996	1.000	Enzyme	Isomerase
Protein ENL inhibitor	Q03111	5	0.8869	0.891	Unclassified protein	NA
Protein farnesyltransferase subunit beta inhibitor	P49356	1555	0.9903	0.99	Enzyme	Transferase
Protein kinase C alpha type inhibitor	P17252	684	0.9803	0.98	Enzyme	Kinase
Protein kinase C beta type inhibitor	P05771	526	0.9853	0.985	Enzyme	Kinase
Protein kinase C delta type inhibitor	Q05655	488	0.992	0.992	Enzyme	Kinase
Protein kinase C epsilon type inhibitor	Q02156	260	0.9743	0.974	Enzyme	Kinase
Protein kinase C eta type inhibitor	P24723	161	0.9892	0.989	Enzyme	Kinase
Protein kinase C gamma type inhibitor	P05129	116	0.9807	0.981	Enzyme	Kinase
Protein kinase C iota type inhibitor	P41743	517	0.9974	0.997	Enzyme	Kinase
Protein kinase C theta type inhibitor	Q04759	728	0.9936	0.992	Enzyme	Kinase
Protein kinase C zeta type inhibitor	Q05513	153	0.9901	0.989	Enzyme	Kinase

Protein max inhibitor	P61244	6	0.8577	0.845	Transcription factor	NA
Protein Mdm4 inhibitor	O15151	161	0.9983	0.998	Unclassified protein	NA
Protein mono-ADP-ribosyltransferase PARP10 inhibitor	Q53GL7	85	0.9884	0.988	Enzyme	Transferase
Protein mono-ADP-ribosyltransferase PARP11 inhibitor	Q9NR21	12	0.9814	0.982	Enzyme	Transferase
Protein mono-ADP-ribosyltransferase PARP12 inhibitor	Q9H0J9	4	0.9837	0.984	Enzyme	Transferase
Protein mono-ADP-ribosyltransferase PARP14 inhibitor	Q460N5	41	0.9832	0.984	Enzyme	Transferase
Protein mono-ADP-ribosyltransferase PARP15 inhibitor	Q460N3	8	0.9873	0.988	Enzyme	Transferase
Protein mono-ADP-ribosyltransferase PARP16 inhibitor	Q8N5Y8	5	0.935	0.937	Enzyme	Transferase
Protein mono-ADP-ribosyltransferase PARP3 inhibitor	Q9Y6F1	88	0.9983	0.998	Enzyme	Transferase
Protein mono-ADP-ribosyltransferase PARP4 inhibitor	Q9UKK3	11	0.9927	0.993	Enzyme	Transferase
Protein mono-ADP-ribosyltransferase PARP6 inhibitor	Q2NL67	3	0.9991	0.999	Enzyme	Transferase
Protein O-GlcNAcase inhibitor	O60502	168	0.9999	1.000	Enzyme	NA
Protein phosphatase 1A inhibitor	P35813	3	0.893	0.895	Enzyme	Phosphatase
Protein phosphatase 1B inhibitor	O75688	11	0.9988	0.999	Enzyme	Phosphatase
Protein phosphatase 3 catalytic subunit alpha inhibitor	Q08209	11	1.000	1.000	Enzyme	Phosphatase
Protein phosphatase methylesterase 1 inhibitor	Q9Y570	10	0.9838	0.984	Enzyme	Hydrolase
Protein S100-A10 inhibitor	P60903	30	0.9485	0.949	Unclassified protein	NA
Protein S100-A4 inhibitor	P26447	17	0.9991	0.999	Unclassified protein	NA
Protein S100-A9 inhibitor	P06702	50	1.000	1.000	Unclassified protein	NA
Protein S100-B inhibitor	P04271	3	0.9932	0.993	Other cytosolic protein	NA
Protein tyrosine phosphatase receptor type C-associated protein antagonist	Q14761	4	0.956	0.957	Enzyme	NA
Protein tyrosine phosphatase type IVA 1 inhibitor	Q93096	4	0.8905	0.873	Enzyme	Phosphatase
Protein tyrosine phosphatase type IVA 2 inhibitor	Q12974	4	0.8905	0.873	Enzyme	NA
Protein tyrosine phosphatase type IVA 3 inhibitor	O75365	20	0.9773	0.978	Enzyme	Phosphatase
Protein Wnt-3a inhibitor	P56704	38	0.9831	0.982	Secreted protein	NA
Protein-arginine deiminase type-1 inhibitor	Q9ULC6	8	1.000	1.000	Enzyme	Hydrolase
Protein-arginine deiminase type-2 inhibitor	Q9Y2J8	11	1.000	1.000	Enzyme	Hydrolase
Protein-arginine deiminase type-3 inhibitor	Q9ULW8	16	0.9999	1.000	Enzyme	Hydrolase
Protein-arginine deiminase type-4 inhibitor	Q9UM07	19	0.9999	1.000	Enzyme	NA
Protein-arginine deiminase type-6 inhibitor	Q6TGC4	6	1.000	1.000	Enzyme	Hydrolase
Proteinase-activated receptor 1 agonist	P25116	76	0.9998	1.000	Membrane receptor	Family A G protein-coupled receptor
Proteinase-activated receptor 1 antagonist	P25116	544	0.9964	0.996	Membrane receptor	Family A G protein-

						coupled receptor
Proteinase-activated receptor 2 agonist	P55085	151	0.9997	1.000	Membrane receptor	Family A G protein-coupled receptor
Proteinase-activated receptor 2 antagonist	P55085	28	0.9327	0.932	Membrane receptor	Family A G protein-coupled receptor
Proteinase-activated receptor 4 antagonist	Q96RI0	885	0.999	0.999	Membrane receptor	Family A G protein-coupled receptor
Protein-glutamine gamma-glutamyltransferase 2 inhibitor	P21980	318	0.9905	0.991	Enzyme	Aminoacyltransferase
Protein-glutamine gamma-glutamyltransferase K inhibitor	P22735	61	0.9724	0.973	Enzyme	Aminoacyltransferase
Protein-lysine 6-oxidase inhibitor	P28300	23	0.9822	0.898	Enzyme	Oxidoreductase
Protein-serine O-palmitoleoyltransferase porcupine inhibitor	Q9H237	57	0.9999	1.000	Enzyme	Transferase
Protein-S-isoprenylcysteine O-methyltransferase inhibitor	O60725	165	0.999	0.999	Enzyme	Transferase
Protein-tyrosine kinase 2-beta inhibitor	Q14289	332	0.9389	0.939	Enzyme	Kinase
Protein-tyrosine kinase 6 inhibitor	Q13882	147	0.9605	0.961	Enzyme	Kinase
Prothrombin inhibitor	P00734	4246	0.9903	0.99	Enzyme	Protease
Proton-coupled folate transporter inhibitor	Q96NT5	33	0.9999	1.000	Transporter	Electrochemical transporter
Proto-oncogene tyrosine-protein kinase receptor Ret antagonist	P07949	566	0.9732	0.973	Enzyme	Kinase
Proto-oncogene tyrosine-protein kinase ROS inhibitor	P08922	150	0.9738	0.974	Enzyme	Kinase
Proto-oncogene tyrosine-protein kinase Src inhibitor	P12931	2783	0.9752	0.975	Enzyme	Kinase
Proto-oncogene Wnt-3 inhibitor	P56703	23	1.000	1.000	Secreted protein	NA
Protoporphyrinogen oxidase inhibitor	P50336	64	0.9975	0.998	Enzyme	Oxidoreductase
P-selectin inhibitor	P16109	65	0.9996	1.000	Adhesion	NA
PTPsigma-(Brain) inhibitor	Q9UM81	7	0.8778	0.88	Unclassified protein	NA
Purine nucleoside phosphorylase inhibitor	P00491	186	0.9999	1.000	Enzyme	Transferase
Puromycin-sensitive aminopeptidase inhibitor	P55786	15	0.9906	0.991	Enzyme	Protease
Putative inactive group IIC secretory phospholipase A2 inhibitor	Q5R387	10	0.9999	1.000	Enzyme	NA
Putative P2Y purinoceptor 10 agonist	O00398	22	1.000	1.000	Membrane receptor	Family A G protein-coupled receptor
Pyroglutamylated RF-amide peptide receptor agonist	Q96P65	100	0.9999	1.000	Membrane receptor	Family A G protein-coupled receptor
Pyroglutamylated RF-amide peptide receptor antagonist	Q96P65	63	0.9952	0.995	Membrane receptor	Family A G protein-coupled receptor
Pyrroline-5-carboxylate reductase 1, mitochondrial inhibitor	P32322	3	1.000	1.000	Enzyme	Oxidoreductase
Pyruvate kinase PKLR inhibitor	P30613	3	1.000	1.000	Enzyme	NA
Pyruvate kinase PKM inhibitor	P14618	1779	0.8595	0.859	Enzyme	NA

RAC-alpha serine/threonine-protein kinase inhibitor	P31749	2676	0.9843	0.984	Enzyme	Kinase
RAC-beta serine/threonine-protein kinase inhibitor	P31751	1020	0.9966	0.996	Enzyme	Kinase
RAC-gamma serine/threonine-protein kinase inhibitor	Q9Y243	237	0.9895	0.988	Enzyme	Kinase
RAF proto-oncogene serine/threonine-protein kinase inhibitor	P04049	1140	0.9871	0.987	Enzyme	Kinase
Rap guanine nucleotide exchange factor 3 inhibitor	O95398	12	0.9131	0.914	Unclassified protein	NA
Rap guanine nucleotide exchange factor 4 inhibitor	Q8WZA2	33	0.9965	0.996	Unclassified protein	NA
Ras guanyl-releasing protein 3 inhibitor	Q8IV61	11	1.000	1.000	Other cytosolic protein	NA
Ras-related C3 botulinum toxin substrate 1 inhibitor	P63000	8	0.8111	0.809	Enzyme	Hydrolase
Ras-related protein Rab-7a inhibitor	P51149	23	1.000	1.000	Enzyme	Hydrolase
Receptor tyrosine-protein kinase erbB-2 antagonist	P04626	1912	0.9863	0.986	Enzyme	Kinase
Receptor tyrosine-protein kinase erbB-3 antagonist	P21860	41	0.9984	0.998	Enzyme	Kinase
Receptor tyrosine-protein kinase erbB-4 antagonist	Q15303	642	0.9852	0.985	Enzyme	Kinase
Receptor-interacting serine/threonine-protein kinase 1 antagonist	Q13546	288	0.9876	0.988	Enzyme	Kinase
Receptor-interacting serine/threonine-protein kinase 2 antagonist	O43353	112	0.9664	0.965	Enzyme	Kinase
Receptor-type tyrosine-protein kinase FLT3 antagonist	P36888	2131	0.98	0.98	Enzyme	Kinase
Receptor-type tyrosine-protein phosphatase alpha antagonist	P18433	23	0.9892	0.989	Enzyme	Phosphatase
Receptor-type tyrosine-protein phosphatase beta antagonist	P23467	138	0.9937	0.989	Enzyme	Phosphatase
Receptor-type tyrosine-protein phosphatase C antagonist	P08575	125	0.9794	0.97	Enzyme	Phosphatase
Receptor-type tyrosine-protein phosphatase epsilon antagonist	P23469	5	1.000	1.000	Enzyme	Phosphatase
Receptor-type tyrosine-protein phosphatase F antagonist	P10586	44	0.9738	0.972	Enzyme; Membrane receptor	Phosphatase
Receptor-type tyrosine-protein phosphatase gamma antagonist	P23470	17	0.9995	1.000	Enzyme	Phosphatase
Receptor-type tyrosine-protein phosphatase O antagonist	Q16827	7	0.9961	0.996	Enzyme	Hydrolase
Receptor-type tyrosine-protein phosphatase S antagonist	Q13332	17	0.9995	1.000	Enzyme	Phosphatase
Receptor-type tyrosine-protein phosphatase zeta antagonist	P23471	5	1.000	1.000	Enzyme	Hydrolase
RecQ-like DNA helicase BLM inhibitor	P54132	499	0.8543	0.854	Enzyme	NA
Reduced folate transporter inhibitor	P41440	25	0.9999	1.000	Transporter	Electrochemical transporter
Regulator of G-protein signaling 12 inhibitor	O14924	34	0.9491	0.951	Enzyme	NA
Regulator of G-protein signaling 17 inhibitor	Q9UGC6	4	0.8766	0.879	Unclassified protein	NA
Regulator of G-protein signaling 4 inhibitor	P49798	39	1.000	1.000	Unclassified protein	NA
Regulator of G-protein signaling 8 inhibitor	P57771	10	1.000	1.000	Enzyme	NA
Relaxin receptor 1 agonist	Q9HBX9	315	0.9208	0.92	Membrane receptor	Family A G protein-coupled receptor

Relaxin-3 receptor 1 agonist	Q9NSD7	19	0.9999	1.000	Membrane receptor	Family A G protein-coupled receptor
Relaxin-3 receptor 1 antagonist	Q9NSD7	10	0.989	0.989	Membrane receptor	Family A G protein-coupled receptor
Relaxin-3 receptor 2 agonist	Q8TDU9	23	0.9999	1.000	Membrane receptor	Family A G protein-coupled receptor
Relaxin-3 receptor 2 antagonist	Q8TDU9	6	0.9998	1.000	Membrane receptor	Family A G protein-coupled receptor
Renin inhibitor	P00797	2998	0.9976	0.998	Enzyme	Protease
REST corepressor 1 inhibitor	Q9UKL0	107	0.9928	0.993	Unclassified protein	NA
REST corepressor 3 inhibitor	Q9P2K3	3	1.000	1.000	Unclassified protein	NA
Retinal dehydrogenase 2 inhibitor	O94788	29	0.9646	0.952	Enzyme	NA
Retinal rod rhodopsin-sensitive cGMP 3',5'-cyclic phosphodiesterase subunit delta inhibitor	O43924	68	0.9999	1.000	Enzyme	Phosphodiesterase
Retinoblastoma-associated protein inhibitor	P06400	16	0.9934	0.993	Unclassified protein	NA
Retinoblastoma-binding protein 5 inhibitor	Q15291	36	0.9999	1.000	Unclassified protein	NA
Retinoic acid receptor alpha agonist	P10276	29	0.9994	0.999	Transcription factor	Nuclear receptor
Retinoic acid receptor alpha antagonist	P10276	86	0.9994	0.999	Transcription factor	Nuclear receptor
Retinoic acid receptor beta agonist	P10826	51	0.9994	0.999	Transcription factor	Nuclear receptor
Retinoic acid receptor beta antagonist	P10826	89	0.9993	0.999	Transcription factor	Nuclear receptor
Retinoic acid receptor gamma agonist	P13631	65	0.9996	1.000	Transcription factor	Nuclear receptor
Retinoic acid receptor gamma antagonist	P13631	86	0.9917	0.992	Transcription factor	Nuclear receptor
Retinoic acid receptor RXR-alpha agonist	P19793	103	0.9986	0.999	Transcription factor	Nuclear receptor
Retinoic acid receptor RXR-alpha antagonist	P19793	232	0.993	0.993	Transcription factor	Nuclear receptor
Retinoic acid receptor RXR-beta agonist	P28702	33	0.9996	1.000	Transcription factor	Nuclear receptor
Retinoic acid receptor RXR-beta antagonist	P28702	50	0.9986	0.999	Transcription factor	Nuclear receptor
Retinoic acid receptor RXR-gamma agonist	P48443	32	0.9998	1.000	Transcription factor	Nuclear receptor
Retinoic acid receptor RXR-gamma antagonist	P48443	69	0.9971	0.997	Transcription factor	Nuclear receptor
Retinol-binding protein 4 inhibitor	P02753	197	0.9994	1.000	Secreted protein	NA
Rho GDP-dissociation inhibitor 1 inhibitor	P52565	6	1.000	1.000	Unclassified protein	NA
Rho-associated protein kinase 1 inhibitor	Q13464	1045	0.9903	0.99	Enzyme	Kinase
Rho-associated protein kinase 2 inhibitor	O75116	1844	0.9881	0.988	Enzyme	Kinase
Rhodopsin kinase GRK1 inhibitor	Q15835	12	0.8981	0.902	Enzyme	Kinase
Rhodopsin kinase GRK7 inhibitor	Q8WTQ7	8	0.8251	0.828	Enzyme	Kinase
Ribonuclease H1 inhibitor	O60930	15	0.9915	0.992	Enzyme	Hydrolase

Ribonucleoside-diphosphate reductase large subunit inhibitor	P23921	22	0.9989	0.999	Enzyme	Oxidoreductase
Ribonucleoside-diphosphate reductase subunit M2 B inhibitor	Q7LG56	48	1.000	1.000	Enzyme	NA
Ribonucleoside-diphosphate reductase subunit M2 inhibitor	P31350	23	1.000	1.000	Enzyme	Oxidoreductase
Ribosomal protein S6 kinase alpha-1 inhibitor	Q15418	83	0.928	0.926	Enzyme	Kinase
Ribosomal protein S6 kinase alpha-2 inhibitor	Q15349	272	0.9875	0.988	Enzyme	Kinase
Ribosomal protein S6 kinase alpha-3 inhibitor	P51812	330	0.9824	0.982	Enzyme	Kinase
Ribosomal protein S6 kinase alpha-5 inhibitor	O75582	103	0.9507	0.949	Enzyme	Kinase
Ribosomal protein S6 kinase alpha-6 inhibitor	Q9UK32	21	0.9393	0.941	Enzyme	Kinase
Ribosomal protein S6 kinase beta-1 inhibitor	P23443	1375	0.9881	0.988	Enzyme	Kinase
Ribosomal protein S6 kinase beta-2 inhibitor	Q9UBS0	25	0.8898	0.892	Enzyme	Kinase
Ribosyldihydronicotinamide dehydrogenase [quinone] inhibitor	P16083	261	0.9889	0.989	Enzyme	Oxidoreductase
RNA-binding protein EWS inhibitor	Q01844	5	1.000	1.000	Unclassified protein	NA
Rod cGMP-specific 3',5'-cyclic phosphodiesterase subunit alpha inhibitor	P16499	44	0.9819	0.982	Enzyme	Phosphodiesterase
Rod cGMP-specific 3',5'-cyclic phosphodiesterase subunit beta inhibitor	P35913	76	0.9879	0.988	Enzyme	Phosphodiesterase
Ryanodine receptor 1 antagonist	P21817	4	0.9502	0.951	Ion channel	Ligand-gated ion channel
Ryanodine receptor 2 antagonist	Q92736	50	1.000	1.000	Ion channel	Ligand-gated ion channel
S-adenosylhomocysteine hydrolase-like protein 1 inhibitor	O43865	3	1.000	1.000	Enzyme	NA
S-adenosylmethionine decarboxylase proenzyme inhibitor	P17707	18	1.000	1.000	Enzyme	Lyase
S-adenosylmethionine synthase isoform type-2 inhibitor	P31153	296	1.000	1.000	Enzyme	Transferase
Sarcoplasmic/endoplasmic reticulum calcium ATPase 1 inhibitor	O14983	8	1.000	1.000	Transporter; Enzyme	Primary active transporter; Hydrolase
Sarcoplasmic/endoplasmic reticulum calcium ATPase 2 inhibitor	P16615	8	0.9813	0.982	Transporter; Enzyme	Primary active transporter; Hydrolase
Sarcoplasmic/endoplasmic reticulum calcium ATPase 3 inhibitor	Q93084	5	0.9598	0.963	Transporter; Enzyme	Primary active transporter; Hydrolase
Scavenger receptor class B member 1 antagonist	Q8WTV0	53	1.000	1.000	Membrane receptor	NA
Sclerostin inhibitor	Q9BQB4	22	0.9995	0.999	Unclassified protein	NA
Secreted frizzled-related protein 1 inhibitor	Q8N474	68	0.9881	0.988	Unclassified protein	NA
Segment polarity protein dishevelled homolog DVL-2 inhibitor	O14641	3	0.9999	1.000	Unclassified protein	NA
Sentrin-specific protease 1 inhibitor	Q9P0U3	21	0.9921	0.992	Enzyme	Protease

Sentrin-specific protease 6 inhibitor	Q9GZR1	297	0.944	0.943	Enzyme	Protease
Sentrin-specific protease 7 inhibitor	Q9BQF6	572	0.9387	0.939	Enzyme	Protease
Sentrin-specific protease 8 inhibitor	Q96LD8	231	0.9498	0.945	Enzyme	Protease
Sepiapterin reductase inhibitor	P35270	1022	0.992	0.991	Enzyme	Oxidoreductase
Serine hydrolase RBBP9 inhibitor	O75884	5	1.000	1.000	Enzyme	Hydrolase
Serine hydroxymethyltransferase, mitochondrial inhibitor	P34897	177	0.9609	0.959	Enzyme	Transferase
Serine palmitoyltransferase 1 inhibitor	O15269	4	0.9866	0.987	Enzyme	Transferase
Serine palmitoyltransferase 2 inhibitor	O15270	8	0.9997	1.000	Enzyme	Transferase
Serine protease 1 inhibitor	P07477	769	0.9958	0.996	Enzyme	Protease
Serine protease hepsin inhibitor	P05981	195	0.9777	0.977	Enzyme	Protease
Serine protease HTRA1 inhibitor	Q92743	170	1.000	1.000	Enzyme	Hydrolase
Serine/threonine-protein kinase 17A inhibitor	Q9UEE5	65	0.9727	0.974	Enzyme	Kinase
Serine/threonine-protein kinase 17B inhibitor	O94768	77	0.9738	0.975	Enzyme	Kinase
Serine/threonine-protein kinase 26 inhibitor	Q9P289	17	0.9072	0.906	Enzyme	Kinase
Serine/threonine-protein kinase 3 inhibitor	Q13188	39	0.9172	0.902	Enzyme	Kinase
Serine/threonine-protein kinase 32A inhibitor	Q8WU08	3	0.983	0.983	Enzyme	Kinase
Serine/threonine-protein kinase 33 inhibitor	Q9BYT3	27	0.9068	0.908	Enzyme	Kinase
Serine/threonine-protein kinase 36 inhibitor	Q9NRP7	4	0.8938	0.907	Enzyme	Kinase
Serine/threonine-protein kinase 4 inhibitor	Q13043	184	0.9918	0.992	Enzyme	Kinase
Serine/threonine-protein kinase A-Raf inhibitor	P10398	64	0.9773	0.977	Enzyme	Kinase
Serine/threonine-protein kinase ATR inhibitor	Q13535	230	0.9908	0.991	Enzyme	Kinase
Serine/threonine-protein kinase B-raf inhibitor	P15056	2881	0.9891	0.989	Enzyme	Kinase
Serine/threonine-protein kinase BRSK1 inhibitor	Q8TDC3	6	0.8732	0.876	Enzyme	Kinase
Serine/threonine-protein kinase BRSK2 inhibitor	Q8IWQ3	12	0.8665	0.865	Enzyme	Kinase
Serine/threonine-protein kinase Chk1 inhibitor	O14757	1898	0.9908	0.991	Enzyme	Kinase
Serine/threonine-protein kinase Chk2 inhibitor	O96017	517	0.9828	0.983	Enzyme	Kinase
Serine/threonine-protein kinase D1 inhibitor	Q15139	125	0.9844	0.985	Enzyme	Kinase
Serine/threonine-protein kinase D2 inhibitor	Q9BZL6	62	0.9369	0.937	Enzyme	Kinase
Serine/threonine-protein kinase DCLK1 inhibitor	O15075	34	0.9834	0.983	Enzyme	Kinase
Serine/threonine-protein kinase DCLK3 inhibitor	Q9C098	3	0.9789	0.979	Enzyme	Kinase
Serine/threonine-protein kinase haspin inhibitor	Q8TF76	38	0.9462	0.949	Enzyme	Kinase
Serine/threonine-protein kinase LATS1 inhibitor	O95835	269	0.9984	0.998	Enzyme	Kinase

Serine/threonine-protein kinase LATS2 inhibitor	Q9NRM7	124	0.9934	0.993	Enzyme	Kinase
Serine/threonine-protein kinase LMTK3 inhibitor	Q96Q04	19	0.9643	0.965	Enzyme	Transferase
Serine/threonine-protein kinase MARK1 inhibitor	Q9P0L2	16	0.8546	0.858	Enzyme	Kinase
Serine/threonine-protein kinase MARK2 inhibitor	Q7KZI7	16	0.9136	0.913	Enzyme	Kinase
Serine/threonine-protein kinase MRCK alpha inhibitor	Q5VT25	30	0.9504	0.912	Enzyme	Kinase
Serine/threonine-protein kinase mTOR inhibitor	P42345	3432	0.9865	0.986	Enzyme	Kinase
Serine/threonine-protein kinase N1 inhibitor	Q16512	23	0.8705	0.852	Enzyme	Kinase
Serine/threonine-protein kinase N2 inhibitor	Q16513	30	0.9519	0.95	Enzyme	Kinase
Serine/threonine-protein kinase Nek1 inhibitor	Q96PY6	197	0.9895	0.99	Enzyme	Kinase
Serine/threonine-protein kinase Nek2 inhibitor	P51955	100	0.9537	0.955	Enzyme	Kinase
Serine/threonine-protein kinase Nek6 inhibitor	Q9HC98	18	0.9675	0.968	Enzyme	Kinase
Serine/threonine-protein kinase Nek7 inhibitor	Q8TDX7	6	0.9709	0.971	Enzyme	Kinase
Serine/threonine-protein kinase NLK inhibitor	Q9UBE8	10	0.8257	0.797	Enzyme	Kinase
Serine/threonine-protein kinase PAK 1 inhibitor	Q13153	216	0.9682	0.968	Enzyme	Kinase
Serine/threonine-protein kinase PAK 2 inhibitor	Q13177	44	0.9781	0.979	Enzyme	Kinase
Serine/threonine-protein kinase PAK 3 inhibitor	O75914	13	0.9457	0.947	Enzyme	Kinase
Serine/threonine-protein kinase PAK 4 inhibitor	O96013	203	0.9816	0.981	Enzyme	Kinase
Serine/threonine-protein kinase pim-1 inhibitor	P11309	2629	0.9896	0.99	Enzyme	Kinase
Serine/threonine-protein kinase pim-2 inhibitor	Q9P1W9	1348	0.9952	0.995	Enzyme	Kinase
Serine/threonine-protein kinase pim-3 inhibitor	Q86V86	912	0.9926	0.993	Enzyme	Kinase
Serine/threonine-protein kinase PLK1 inhibitor	P53350	724	0.9841	0.984	Enzyme	Kinase
Serine/threonine-protein kinase PLK2 inhibitor	Q9NYY3	179	0.9827	0.983	Enzyme	Kinase
Serine/threonine-protein kinase PLK3 inhibitor	Q9H4B4	163	0.9564	0.957	Enzyme	Kinase
Serine/threonine-protein kinase PLK4 inhibitor	O00444	156	0.9759	0.972	Enzyme	Kinase
Serine/threonine-protein kinase receptor R3 inhibitor	P37023	66	0.9709	0.972	Enzyme	Kinase
Serine/threonine-protein kinase Sgk1 inhibitor	O00141	144	0.95	0.949	Enzyme	Kinase
Serine/threonine-protein kinase Sgk2 inhibitor	Q9HBY8	6	0.8378	0.838	Enzyme	Kinase
Serine/threonine-protein kinase SMG1 inhibitor	Q96Q15	27	1.000	1.000	Enzyme	Kinase
Serine/threonine-protein kinase STK11 inhibitor	Q15831	7	0.8607	0.862	Enzyme	Kinase
Serine/threonine-protein kinase TAO1 inhibitor	Q7L7X3	65	0.9546	0.949	Enzyme	Kinase
Serine/threonine-protein kinase TAO3 inhibitor	Q9H2K8	69	0.959	0.954	Enzyme	Kinase
Serine/threonine-protein kinase TBK1 inhibitor	Q9UHD2	679	0.9953	0.995	Enzyme	Kinase

Serine/threonine-protein kinase TNNI3K inhibitor	Q59H18	62	0.999	0.999	Enzyme	Kinase
Serine/threonine-protein kinase ULK1 activator	O75385	4	0.9999	1.000	Enzyme	Kinase
Serine/threonine-protein kinase ULK1 inhibitor	O75385	43	0.9554	0.956	Enzyme	Kinase
Serine/threonine-protein kinase ULK2 inhibitor	Q8IYT8	14	0.9132	0.914	Enzyme	Kinase
Serine/threonine-protein kinase VRK1 inhibitor	Q99986	5	0.9502	0.951	Enzyme	Kinase
Serine/threonine-protein kinase VRK2 inhibitor	Q86Y07	4	0.9638	0.965	Enzyme	NA
Serine/threonine-protein kinase WNK1 allosteric inhibitor	Q9H4A3	11	0.9998	1.000	Enzyme	Kinase
Serine/threonine-protein kinase WNK1 inhibitor	Q9H4A3	12	0.9377	0.938	Enzyme	Kinase
Serine/threonine-protein kinase WNK2 inhibitor	Q9Y3S1	7	0.8992	0.902	Enzyme	Kinase
Serine/threonine-protein kinase WNK3 inhibitor	Q9BYP7	7	0.8395	0.845	Enzyme	Kinase
Serine/threonine-protein kinase/endoribonuclease IRE1 inhibitor	O75460	210	0.9898	0.99	Enzyme	NA
Serine/threonine-protein phosphatase 2A 56 kDa regulatory subunit alpha isoform inhibitor	Q15172	3	0.998	0.998	Enzyme	Phosphatase
Serine/threonine-protein phosphatase 2A activator inhibitor	Q15257	4	1.000	1.000	Enzyme	Phosphatase
Serine/threonine-protein phosphatase 2A catalytic subunit alpha isoform inhibitor	P67775	9	0.983	0.983	Enzyme	Phosphatase
Serine/threonine-protein phosphatase inhibitor	Q9BPW0	24	0.8829	0.834	Enzyme	Phosphatase
Serine/threonine-protein phosphatase PP1-alpha catalytic subunit inhibitor	P62136	61	0.9177	0.909	Enzyme	Phosphatase
Serine/threonine-protein phosphatase PP1-gamma catalytic subunit inhibitor	P36873	20	0.9999	1.000	Enzyme	Phosphatase
Serine-protein kinase ATM inhibitor	Q13315	154	0.9922	0.992	Enzyme	Kinase
Serum paraoxonase/arylesterase 1 inhibitor	P27169	3	0.9952	0.995	Enzyme	Hydrolase
Short transient receptor potential channel 3 blocker	Q13507	32	0.977	0.977	Ion channel	Voltage-gated ion channel
Short transient receptor potential channel 5 blocker	Q9UL62	74	0.9922	0.992	Ion channel	Voltage-gated ion channel
Short transient receptor potential channel 6 blocker	Q9Y210	39	1.000	1.000	Ion channel	Voltage-gated ion channel
Short transient receptor potential channel 7 blocker	Q9HCX4	5	0.8735	0.848	Unclassified protein	NA
Sialidase-1 inhibitor	Q99519	22	1.000	1.000	Enzyme	Hydrolase
Sialidase-2 inhibitor	Q9Y3R4	12	1.000	1.000	Enzyme	Hydrolase
Sialidase-3 inhibitor	Q9UQ49	31	0.9999	1.000	Enzyme	Hydrolase
Sialidase-4 inhibitor	Q8WWR8	23	1.000	1.000	Enzyme	Hydrolase
Sigma intracellular receptor 2 antagonist	Q5BJF2	50	0.8334	0.835	Membrane receptor	NA
Sigma non-opioid intracellular receptor 1 antagonist	Q99720	1377	0.9882	0.988	Membrane receptor	NA
Signal peptide peptidase-like 2A inhibitor	Q8TCT8	13	0.9964	0.997	Enzyme	Hydrolase

Signal transducer and activator of transcription 1-alpha/beta inhibitor	P42224	9	0.8808	0.881	Transcription factor	NA
Signal transducer and activator of transcription 3 inhibitor	P40763	264	0.976	0.976	Transcription factor	NA
Signal transducer and activator of transcription 5B inhibitor	P51692	10	0.9839	0.983	Transcription factor	NA
Signal transducer and activator of transcription 6 inhibitor	P42226	102	0.9994	1.000	Transcription factor	NA
Small conductance calcium-activated potassium channel protein 1 blocker	Q92952	9	0.8069	0.772	Ion channel	Voltage-gated ion channel
Small conductance calcium-activated potassium channel protein 2 blocker	Q9H2S1	11	0.9604	0.933	Ion channel	Voltage-gated ion channel
Small conductance calcium-activated potassium channel protein 3 blocker	Q9UGI6	22	0.9363	0.928	Ion channel	Voltage-gated ion channel
S-methyl-5'-thioadenosine phosphorylase inhibitor	Q13126	44	1.000	1.000	Enzyme	NA
Smoothened homolog inhibitor	Q99835	196	0.9941	0.994	Membrane receptor	Frizzled family G protein-coupled receptor
Sodium- and chloride-dependent betaine transporter inhibitor	P48065	9	0.9789	0.976	Transporter	Electrochemical transporter
Sodium- and chloride-dependent GABA transporter 1 inhibitor	P30531	56	0.9998	1.000	Transporter	Electrochemical transporter
Sodium- and chloride-dependent GABA transporter 2 inhibitor	Q9NSD5	4	0.8891	0.892	Transporter	Electrochemical transporter
Sodium- and chloride-dependent GABA transporter 3 inhibitor	P48066	9	0.9662	0.967	Transporter	Electrochemical transporter
Sodium- and chloride-dependent glycine transporter 1 inhibitor	P48067	1253	0.9976	0.998	Transporter	Electrochemical transporter
Sodium- and chloride-dependent glycine transporter 2 inhibitor	Q9Y345	143	0.998	0.998	Transporter	Electrochemical transporter
Sodium channel protein type 1 subunit alpha blocker	P35498	49	0.9718	0.973	Ion channel	Voltage-gated ion channel
Sodium channel protein type 10 subunit alpha blocker	Q9Y5Y9	189	0.9726	0.973	Ion channel	Voltage-gated ion channel
Sodium channel protein type 2 subunit alpha blocker	Q99250	154	0.9708	0.968	Ion channel	Voltage-gated ion channel
Sodium channel protein type 3 subunit alpha blocker	Q9NY46	71	0.9421	0.943	Ion channel	Voltage-gated ion channel
Sodium channel protein type 4 subunit alpha blocker	P35499	131	0.9747	0.975	Ion channel	Voltage-gated ion channel
Sodium channel protein type 5 subunit alpha activator	Q14524	9	1.000	1.000	Ion channel	Voltage-gated ion channel
Sodium channel protein type 5 subunit alpha blocker	Q14524	695	0.9769	0.977	Ion channel	Voltage-gated ion channel
Sodium channel protein type 7 subunit alpha blocker	Q01118	14	0.8522	0.858	Ion channel	Voltage-gated ion

						channel
Sodium channel protein type 8 subunit alpha blocker	Q9UQD0	61	0.9455	0.945	Ion channel	Voltage-gated ion channel
Sodium channel protein type 9 subunit alpha blocker	Q15858	5312	0.9902	0.99	Ion channel	Voltage-gated ion channel
Sodium/bile acid cotransporter inhibitor	Q14973	19	0.8855	0.887	Transporter	Electrochemical transporter
Sodium/glucose cotransporter 1 inhibitor	P13866	875	0.9968	0.997	Transporter	Electrochemical transporter
Sodium/glucose cotransporter 2 inhibitor	P31639	1210	0.9993	0.999	Transporter	Electrochemical transporter
Sodium/hydrogen exchanger 1 inhibitor	P19634	220	0.9999	1.000	Transporter	Electrochemical transporter
Sodium/hydrogen exchanger 2 inhibitor	Q9UBY0	7	0.9571	0.958	Transporter	Electrochemical transporter
Sodium/hydrogen exchanger 3 inhibitor	P48764	204	1.000	1.000	Transporter	Electrochemical transporter
Sodium/hydrogen exchanger 5 inhibitor	Q14940	4	0.8188	0.823	Transporter	Electrochemical transporter
Sodium/nucleoside cotransporter 2 inhibitor	O43868	24	1.000	1.000	Transporter	Electrochemical transporter
Sodium/potassium-transporting ATPase subunit alpha-1 inhibitor	P05023	4	1.000	1.000	Transporter; Enzyme	Primary active transporter; Hydrolase
Sodium/potassium-transporting ATPase subunit beta-2 inhibitor	P14415	148	0.9987	0.999	Transporter; Enzyme	Primary active transporter; Hydrolase
Sodium-dependent dopamine transporter inhibitor	Q01959	1476	0.9888	0.988	Transporter	Electrochemical transporter
Sodium-dependent neutral amino acid transporter B(0)AT2 inhibitor	Q9H2J7	11	1.000	1.000	Transporter	Electrochemical transporter
Sodium-dependent noradrenaline transporter inhibitor	P23975	1869	0.9928	0.993	Transporter	Electrochemical transporter
Sodium-dependent phosphate transport protein 2A inhibitor	Q06495	9	0.9086	0.91	Transporter	Electrochemical transporter
Sodium-dependent proline transporter inhibitor	Q99884	49	0.9999	1.000	Transporter	Electrochemical transporter
Sodium-dependent serotonin transporter inhibitor	P31645	2538	0.9935	0.993	Transporter	Electrochemical transporter
Solute carrier family 13 member 5 inhibitor	Q86YT5	7	1.000	1.000	Transporter	Electrochemical transporter
Solute carrier family 15 member 1 inhibitor	P46059	6	0.971	0.971	Transporter	Electrochemical transporter

Solute carrier family 15 member 2 inhibitor	Q16348	21	0.9955	0.996	Transporter	Electrochemical transporter
Solute carrier family 2, facilitated glucose transporter member 1 inhibitor	P11166	180	0.9732	0.966	Transporter	Electrochemical transporter
Solute carrier family 2, facilitated glucose transporter member 2 inhibitor	P11168	85	0.9999	1.000	Transporter	Electrochemical transporter
Solute carrier family 2, facilitated glucose transporter member 3 inhibitor	P11169	86	0.9951	0.995	Transporter	Electrochemical transporter
Solute carrier family 22 member 1 inhibitor	O15245	36	0.8819	0.887	Transporter	Electrochemical transporter
Solute carrier family 22 member 11 inhibitor	Q9NSA0	10	0.971	0.972	Transporter	Electrochemical transporter
Solute carrier family 22 member 12 inhibitor	Q96S37	370	0.9978	0.998	Transporter	Electrochemical transporter
Solute carrier family 22 member 2 inhibitor	O15244	35	0.8196	0.819	Transporter	Electrochemical transporter
Solute carrier family 22 member 3 inhibitor	O75751	19	0.9885	0.988	Transporter	Electrochemical transporter
Solute carrier family 22 member 6 inhibitor	Q4U2R8	30	0.9384	0.939	Transporter	Electrochemical transporter
Solute carrier family 22 member 8 inhibitor	Q8TCC7	28	0.9096	0.912	Transporter	Electrochemical transporter
Solute carrier family 28 member 3 inhibitor	Q9HAS3	3	0.9976	0.998	Transporter	Electrochemical transporter
Solute carrier family 40 member 1 inhibitor	Q9NP59	176	0.9941	0.991	Transporter	Electrochemical transporter
Solute carrier family 5 member 4 inhibitor	Q9NY91	26	0.9999	1.000	Transporter	Electrochemical transporter
Solute carrier organic anion transporter family member 1B1 inhibitor	Q9Y6L6	41	0.9146	0.916	Transporter	Electrochemical transporter
Solute carrier organic anion transporter family member 1B3 inhibitor	Q9NPD5	15	0.9188	0.918	Transporter	Electrochemical transporter
Solute carrier organic anion transporter family member 2A1 inhibitor	Q92959	3	1.000	1.000	Transporter	Electrochemical transporter
Solute carrier organic anion transporter family member 2B1 inhibitor	O94956	9	0.8251	0.829	Transporter	Electrochemical transporter
Solute carrier organic anion transporter family member 4C1 inhibitor	Q6ZQN7	3	0.9539	0.955	Transporter	Electrochemical transporter
Somatostatin receptor type 1 agonist	P30872	4	0.9999	1.000	Membrane receptor	Family A G protein-coupled receptor
Somatostatin receptor type 1 antagonist	P30872	203	0.995	0.995	Membrane receptor	Family A G protein-coupled receptor
Somatostatin receptor type 2 agonist	P30874	17	1.000	1.000	Membrane receptor	Family A G protein-

						coupled receptor
Somatostatin receptor type 2 antagonist	P30874	335	0.9963	0.996	Membrane receptor	Family A G protein-coupled receptor
Somatostatin receptor type 3 agonist	P32745	88	1.000	1.000	Membrane receptor	Family A G protein-coupled receptor
Somatostatin receptor type 3 antagonist	P32745	414	0.9993	0.999	Membrane receptor	Family A G protein-coupled receptor
Somatostatin receptor type 4 agonist	P31391	8	1.000	1.000	Membrane receptor	Family A G protein-coupled receptor
Somatostatin receptor type 4 antagonist	P31391	217	0.9954	0.996	Membrane receptor	Family A G protein-coupled receptor
Somatostatin receptor type 5 antagonist	P35346	523	0.998	0.998	Membrane receptor	Family A G protein-coupled receptor
Son of sevenless homolog 1 inhibitor	Q07889	201	0.9987	0.999	Unclassified protein	NA
Sonic hedgehog protein inhibitor	Q15465	6	0.8539	0.856	Unclassified protein	NA
Sorbitol dehydrogenase inhibitor	Q00796	58	1.000	1.000	Enzyme	Oxidoreductase
Sortilin inhibitor	Q99523	18	0.9255	0.926	Membrane receptor	NA
Spermidine synthase inhibitor	P19623	3	1.000	1.000	Enzyme	Transferase
Sphingomyelin phosphodiesterase 3 inhibitor	Q9NY59	93	0.9985	0.999	Enzyme	Hydrolase
Sphingomyelin phosphodiesterase inhibitor	P17405	32	0.9911	0.991	Enzyme	Phosphodiesterase
Sphingosine 1-phosphate receptor 1 agonist	P21453	1543	0.9932	0.993	Membrane receptor	Family A G protein-coupled receptor
Sphingosine 1-phosphate receptor 1 antagonist	P21453	698	0.9811	0.981	Membrane receptor	Family A G protein-coupled receptor
Sphingosine 1-phosphate receptor 2 agonist	O95136	34	0.9495	0.95	Membrane receptor	Family A G protein-coupled receptor
Sphingosine 1-phosphate receptor 2 antagonist	O95136	168	0.9417	0.941	Membrane receptor	Family A G protein-coupled receptor
Sphingosine 1-phosphate receptor 3 agonist	Q99500	590	0.9966	0.997	Membrane receptor	Family A G protein-coupled receptor
Sphingosine 1-phosphate receptor 3 antagonist	Q99500	306	0.9844	0.985	Membrane receptor	Family A G protein-coupled receptor
Sphingosine 1-phosphate receptor 4 agonist	O95977	160	0.9863	0.987	Membrane receptor	Family A G protein-coupled receptor
Sphingosine 1-phosphate receptor 4 antagonist	O95977	205	0.9532	0.954	Membrane receptor	Family A G protein-coupled receptor
Sphingosine 1-phosphate receptor 5 agonist	Q9H228	197	0.9953	0.995	Membrane receptor	Family A G protein-coupled receptor
Sphingosine 1-phosphate receptor 5 antagonist	Q9H228	43	0.9617	0.961	Membrane receptor	Family A G protein-coupled receptor

Sphingosine kinase 1 inhibitor	Q9NYA1	179	0.995	0.995	Enzyme	NA
Sphingosine kinase 2 inhibitor	Q9NRA0	120	0.9946	0.994	Enzyme	NA
Sphingosine-1-phosphate lyase 1 inhibitor	O95470	26	1.000	1.000	Enzyme	Lyase
Spindlin-1 inhibitor	Q9Y657	5	0.999	0.999	Epigenetic regulator	Reader
Splicing factor 3B subunit 3 inhibitor	Q15393	12	0.9852	0.986	Other nuclear protein	NA
Squalene monooxygenase inhibitor	Q14534	55	0.9999	1.000	Enzyme	Oxidoreductase
Squalene synthase inhibitor	P37268	167	0.9975	0.998	Enzyme	NA
SRSF protein kinase 2 inhibitor	P78362	6	0.8155	0.818	Enzyme	Kinase
STAM-binding protein inhibitor	O95630	21	0.9984	0.999	Enzyme	Hydrolase
STE20/SPS1-related proline-alanine-rich protein kinase inhibitor	Q9UEW8	5	0.8231	0.825	Enzyme	Kinase
Stearoyl-CoA desaturase 5 inhibitor	Q86SK9	3	0.9988	0.999	Enzyme	Oxidoreductase
Stearoyl-CoA desaturase inhibitor	O00767	488	0.9983	0.998	Enzyme	NA
Steroid 17-alpha-hydroxylase/17,20 lyase inhibitor	P05093	637	0.9982	0.998	Enzyme	Cytochrome P450
Steroid 21-hydroxylase inhibitor	P08686	105	0.999	0.999	Enzyme	Cytochrome P450
Steroid hormone receptor ERR1 agonist	P11474	13	0.863	0.866	Transcription factor	Nuclear receptor
Steroid hormone receptor ERR1 antagonist	P11474	152	0.9959	0.996	Transcription factor	Nuclear receptor
Steroid hormone receptor ERR2 agonist	O95718	20	0.9617	0.962	Transcription factor	Nuclear receptor
Steroid hormone receptor ERR2 antagonist	O95718	37	0.9985	0.999	Transcription factor	Nuclear receptor
Steroidogenic factor 1 inhibitor	Q13285	38	1.000	1.000	Transcription factor	Nuclear receptor
Sterol 26-hydroxylase, mitochondrial inhibitor	Q02318	5	1.000	1.000	Enzyme	Cytochrome P450
Sterol O-acyltransferase 1 inhibitor	P35610	276	0.992	0.992	Enzyme	Transferase
Sterol O-acyltransferase 2 inhibitor	O75908	122	0.9881	0.989	Enzyme	Transferase
Sterol regulatory element-binding protein 2 inhibitor	Q12772	17	0.969	0.969	Transcription factor	NA
Steryl-sulfatase inhibitor	P08842	371	0.9986	0.999	Enzyme	Hydrolase
Stimulator of interferon genes protein inhibitor	Q86WV6	22	0.9997	1.000	Unclassified protein	NA
Stromal cell-derived factor 1 inhibitor	P48061	28	0.9997	1.000	Secreted protein	NA
Stromal interaction molecule 1 inhibitor	Q13586	93	0.9998	1.000	Unclassified protein	NA
Stromelysin-1 inhibitor	P08254	1588	0.9914	0.991	Enzyme	Protease
Stromelysin-2 inhibitor	P09238	138	0.9967	0.997	Enzyme	Protease
Stromelysin-3 inhibitor	P24347	5	0.9744	0.975	Enzyme	Protease
Substance-K receptor antagonist	P21452	496	0.9902	0.99	Membrane receptor	Family A G protein-coupled receptor
Substance-P receptor agonist	P25103	5	1.000	1.000	Membrane receptor	Family A G protein-

						coupled receptor
Substance-P receptor antagonist	P25103	1196	0.9949	0.995	Membrane receptor	Family A G protein-coupled receptor
Succinate dehydrogenase [ubiquinone] iron-sulfur subunit, mitochondrial inhibitor	P21912	34	1.000	1.000	Enzyme	Oxidoreductase
Succinate receptor 1 antagonist	Q9BXA5	47	1.000	1.000	Membrane receptor	Family A G protein-coupled receptor
Sucrase-isomaltase, intestinal inhibitor	P14410	37	1.000	1.000	Enzyme	NA
Sulfotransferase 1A1 inhibitor	P50225	5	0.9862	0.986	Enzyme	Transferase
SUMO-activating enzyme subunit 1 inhibitor	Q9UBE0	4	0.9996	1.000	Unclassified protein	NA
SUMO-activating enzyme subunit 2 inhibitor	Q9UBT2	31	0.9999	1.000	Enzyme	Aminoacyltransferase
SUMO-conjugating enzyme UBC9 inhibitor	P63279	3	1.000	1.000	Enzyme	Aminoacyltransferase
Suppressor of tumorigenicity 14 protein inhibitor	Q9Y5Y6	477	0.9954	0.995	Enzyme	Protease
Synaptic vesicular amine transporter inhibitor	Q05940	20	0.9586	0.96	Transporter	Electrochemical transporter
Synaptojanin-2 inhibitor	O15056	25	0.979	0.98	Enzyme	Hydrolase
Tachykinin-3 inhibitor	Q9UHF0	6	1.000	1.000	Secreted protein	NA
Target of rapamycin complex subunit LST8 inhibitor	Q9BVC4	96	0.9815	0.981	Unclassified protein	NA
Taste receptor type 1 member 2 agonist	Q8TE23	105	0.9907	0.991	Membrane receptor	Family C G protein-coupled receptor
Taste receptor type 1 member 3 agonist	Q7RTX0	138	0.9988	0.999	Membrane receptor	Family C G protein-coupled receptor
Taste receptor type 1 member 3 antagonist	Q7RTX0	243	0.9967	0.997	Membrane receptor	Family C G protein-coupled receptor
Taste receptor type 2 member 14 antagonist	Q9NYV8	101	0.9991	0.999	Membrane receptor	Taste family G protein-coupled receptor
Taste receptor type 2 member 31 antagonist	P59538	4	0.9325	0.933	Membrane receptor	Taste family G protein-coupled receptor
Taste receptor type 2 member 46 agonist	P59540	5	0.8816	0.884	Membrane receptor	Taste family G protein-coupled receptor
Taste receptor type 2 member 8 antagonist	Q9NYW2	302	0.9997	1.000	Membrane receptor	Taste family G protein-coupled receptor
Telomerase reverse transcriptase inhibitor	O14746	506	0.9869	0.986	Enzyme	Transferase
Telomeric repeat-binding factor 2-interacting protein 1 inhibitor	Q9NYB0	12	1.000	1.000	Unclassified protein	NA
Testis-specific serine/threonine-protein kinase 1 inhibitor	Q9BXA7	13	0.9	0.904	Enzyme	Kinase

Testis-specific serine/threonine-protein kinase 2 inhibitor	Q96PF2	5	0.817	0.836	Enzyme	Kinase
Testis-specific serine/threonine-protein kinase 3 inhibitor	Q96PN8	4	0.835	0.838	Enzyme	Kinase
TGF-beta receptor type-1 antagonist	P36897	1009	0.9921	0.992	Enzyme	Kinase
TGF-beta receptor type-2 antagonist	P37173	79	0.9747	0.975	Enzyme	Kinase
TGF-beta-activated kinase 1 and MAP3K7-binding protein 1 inhibitor	Q15750	35	0.9784	0.979	Unclassified protein	NA
Thiopurine S-methyltransferase inhibitor	P51580	6	0.9027	0.904	Enzyme	NA
Thioredoxin inhibitor	P10599	12	0.9206	0.921	Enzyme	Oxidoreductase
Thioredoxin reductase 1, cytoplasmic inhibitor	Q16881	9	0.8684	0.864	Enzyme	Oxidoreductase
Thioredoxin reductase 3 inhibitor	Q86VQ6	27	0.9898	0.961	Enzyme	Oxidoreductase
Thiosulfate sulfurtransferase inhibitor	Q16762	16	0.9604	0.962	Enzyme	Transferase
Threonine aspartase 1 inhibitor	Q9H6P5	8	0.9798	0.98	Enzyme	Protease
Threonine--tRNA ligase 1, cytoplasmic inhibitor	P26639	18	1.000	1.000	Enzyme	Ligase
Thrombopoietin receptor agonist	P40238	202	0.9998	1.000	Membrane receptor	NA
Thrombopoietin receptor antagonist	P40238	9	1.000	1.000	Membrane receptor	NA
Thrombospondin-1 inhibitor	P07996	11	1.000	1.000	Unclassified protein	NA
Thromboxane A2 receptor agonist	P21731	26	1.000	1.000	Membrane receptor	Family A G protein-coupled receptor
Thromboxane A2 receptor antagonist	P21731	615	0.9964	0.996	Membrane receptor	Family A G protein-coupled receptor
Thromboxane-A synthase inhibitor	P24557	713	0.9882	0.988	Enzyme	Cytochrome P450
Thymidine kinase 2, mitochondrial inhibitor	O00142	26	1.000	1.000	Enzyme	Transferase
Thymidine kinase, cytosolic inhibitor	P04183	28	0.9998	1.000	Enzyme	Transferase
Thymidine phosphorylase inhibitor	P19971	93	0.9986	0.999	Enzyme	NA
Thymidylate synthase inhibitor	P04818	585	0.9982	0.998	Enzyme	Transferase
Thyroid hormone receptor alpha agonist	P10827	32	0.9996	1.000	Transcription factor	Nuclear receptor
Thyroid hormone receptor alpha antagonist	P10827	207	0.9988	0.999	Transcription factor	Nuclear receptor
Thyroid hormone receptor beta agonist	P10828	72	0.9998	1.000	Transcription factor	Nuclear receptor
Thyroid hormone receptor beta antagonist	P10828	332	0.9995	1.000	Transcription factor	Nuclear receptor
Thyroid peroxidase inhibitor	P07202	8	0.9914	0.968	Enzyme	Oxidoreductase
Thyrotropin receptor agonist	P16473	5651	0.8809	0.881	Membrane receptor	Family A G protein-coupled receptor
Thyrotropin receptor antagonist	P16473	21	0.9957	0.996	Membrane receptor	Family A G protein-coupled receptor
Thyrotropin-releasing hormone receptor agonist	P34981	7	1.000	1.000	Membrane receptor	Family A G protein-coupled receptor

Tissue alpha-L-fucosidase inhibitor	P04066	58	0.9991	0.999	Enzyme	Hydrolase
Tissue factor inhibitor	P13726	501	0.9945	0.995	Membrane receptor; Surface antigen	NA
Tissue factor pathway inhibitor inhibitor	P10646	549	0.9998	1.000	Unclassified protein	NA
Tissue-type plasminogen activator inhibitor	P00750	258	0.9882	0.988	Enzyme	Protease
TNF receptor-associated factor 6 inhibitor	Q9Y4K3	3	0.9998	1.000	Enzyme	Ligase
Toll-like receptor 2 agonist	O60603	45	0.9926	0.993	Membrane receptor	Toll-like and Il-1 receptors
Toll-like receptor 2 antagonist	O60603	18	0.9833	0.983	Membrane receptor	Toll-like and Il-1 receptors
Toll-like receptor 4 agonist	O00206	47	1.000	1.000	Membrane receptor	Toll-like and Il-1 receptors
Toll-like receptor 4 antagonist	O00206	33	0.9648	0.965	Membrane receptor	Toll-like and Il-1 receptors
Toll-like receptor 7 agonist	Q9NYK1	271	0.9996	1.000	Membrane receptor	Toll-like and Il-1 receptors
Toll-like receptor 7 antagonist	Q9NYK1	97	0.9893	0.99	Membrane receptor	Toll-like and Il-1 receptors
Toll-like receptor 8 agonist	Q9NR97	105	0.9999	1.000	Membrane receptor	Toll-like and Il-1 receptors
Toll-like receptor 8 antagonist	Q9NR97	149	0.9904	0.991	Membrane receptor	Toll-like and Il-1 receptors
Toll-like receptor 9 antagonist	Q9NR96	133	0.9954	0.996	Membrane receptor	Toll-like and Il-1 receptors
Tolloid-like protein 1 inhibitor	O43897	392	1.000	1.000	Enzyme	Hydrolase
Tolloid-like protein 2 inhibitor	Q9Y6L7	388	1.000	1.000	Enzyme	Hydrolase
TP53-binding protein 1 inhibitor	Q12888	5	0.998	0.998	Epigenetic regulator	Reader
Trace amine-associated receptor 1 agonist	Q96RJ0	384	0.965	0.965	Membrane receptor	Family A G protein-coupled receptor
Trace amine-associated receptor 1 antagonist	Q96RJ0	35	0.8168	0.82	Membrane receptor	Family A G protein-coupled receptor
TRAF2 and NCK-interacting protein kinase inhibitor	Q9UKE5	43	0.9366	0.932	Enzyme	Kinase
Transcription activator BRG1 inhibitor	P51532	18	0.97	0.973	Epigenetic regulator	Reader
Transcription factor 4 inhibitor	P15884	6	0.9019	0.904	Transcription factor	NA
Transcription factor 7-like 2 inhibitor	Q9NQB0	253	0.9961	0.996	Transcription factor	NA
Transcription factor AP-1 inhibitor	P05412	163	0.9709	0.971	Transcription factor	NA
Transcription factor p65 inhibitor	Q04206	68	0.9841	0.985	Transcription factor	NA
Transcription initiation factor IIA subunit 1 inhibitor	P52655	19	1.000	1.000	Transcription factor	NA

Transcription initiation factor TFIID subunit 1 inhibitor	P21675	76	0.996	0.992	Enzyme	NA
Transcription intermediary factor 1-alpha inhibitor	O15164	82	0.979	0.979	Epigenetic regulator	Reader
Transcriptional coactivator YAP1 inhibitor	P46937	43	1.000	1.000	Unclassified protein	NA
Transcriptional enhancer factor TEF-3 inhibitor	Q15561	10	0.9699	0.97	Transcription factor	NA
Transcriptional regulator ERG inhibitor	P11308	7	1.000	1.000	Unclassified protein	NA
Transforming protein RhoA inhibitor	P61586	36	0.9993	0.999	Enzyme	Hydrolase
Transient receptor potential cation channel subfamily A member 1 blocker	O75762	528	0.9904	0.991	Ion channel	Voltage-gated ion channel
Transient receptor potential cation channel subfamily M member 4 blocker	Q8TD43	9	0.9999	1.000	Ion channel	Voltage-gated ion channel
Transient receptor potential cation channel subfamily M member 5 blocker	Q9NZQ8	62	1.000	1.000	Ion channel	Voltage-gated ion channel
Transient receptor potential cation channel subfamily M member 8 blocker	Q7Z2W7	458	0.9954	0.995	Ion channel	Voltage-gated ion channel
Transient receptor potential cation channel subfamily V member 1 blocker	Q8NER1	2837	0.9946	0.995	Ion channel	Voltage-gated ion channel
Transient receptor potential cation channel subfamily V member 3 blocker	Q8NET8	238	0.9975	0.998	Ion channel	Voltage-gated ion channel
Transient receptor potential cation channel subfamily V member 4 blocker	Q9HBA0	419	0.996	0.996	Ion channel	Voltage-gated ion channel
Transient receptor potential cation channel subfamily V member 6 blocker	Q9H1D0	6	0.8207	0.821	Ion channel	Voltage-gated ion channel
Transitional endoplasmic reticulum ATPase inhibitor	P55072	284	0.9953	0.995	Transporter	Primary active transporter
Transketolase inhibitor	P29401	49	0.9993	0.999	Enzyme	Transferase
Translocator protein inhibitor	P30536	51	0.9706	0.971	Membrane receptor	NA
Transmembrane domain-containing protein TMIGD3 inhibitor	P0DMS9	24	0.9998	1.000	Unclassified protein	NA
Transmembrane prolyl 4-hydroxylase inhibitor	Q9NXG6	59	0.9997	1.000	Enzyme	Oxidoreductase
Transmembrane protease serine 11D inhibitor	O60235	61	0.9999	1.000	Enzyme	Protease
Transmembrane protease serine 2 inhibitor	O15393	3	1.000	1.000	Enzyme	Protease
Transmembrane protease serine 4 inhibitor	Q9NRS4	13	0.9998	1.000	Enzyme	Protease
Transmembrane protease serine 6 inhibitor	Q8IU80	122	0.9989	0.999	Enzyme	Protease
Transthyretin inhibitor	P02766	74	0.9724	0.973	Secreted protein	NA
Trifunctional purine biosynthetic protein adenosine-3 inhibitor	P22102	117	0.9924	0.992	Enzyme	Ligase
Trypsin-3 inhibitor	P35030	721	0.9908	0.991	Enzyme	Protease
Tryptase alpha/beta-1 inhibitor	Q15661	230	0.9963	0.996	Enzyme	Protease
Tryptase beta-2 inhibitor	P20231	8	1.000	1.000	Enzyme	Hydrolase

Tryptase gamma inhibitor	Q9NRR2	205	0.9944	0.995	Enzyme	Protease
Tryptophan 2,3-dioxygenase inhibitor	P48775	342	0.9985	0.998	Enzyme	Oxidoreductase
Tryptophan 5-hydroxylase 1 inhibitor	P17752	235	1.000	1.000	Enzyme	Oxidoreductase
Tryptophan 5-hydroxylase 2 inhibitor	Q8IWU9	5	0.9999	1.000	Enzyme	Oxidoreductase
Tubulin alpha-3C chain inhibitor	P0DPH7	598	0.9942	0.994	Structural protein	NA
Tubulin beta chain inhibitor	P07437	5	0.8944	0.896	Structural protein	NA
Tubulin beta-1 chain inhibitor	Q9H4B7	47	0.9994	0.999	Structural protein	NA
Tubulin beta-3 chain inhibitor	Q13509	7	0.8568	0.857	Structural protein	NA
Tumor necrosis factor inhibitor	P01375	348	0.9958	0.995	Secreted protein	NA
Tumor necrosis factor receptor superfamily member 10A antagonist	O00220	3	0.9993	0.999	Membrane receptor	NA
Tumor necrosis factor receptor superfamily member 16 antagonist	P08138	5	0.9933	0.994	Membrane receptor	NA
Tumor necrosis factor receptor superfamily member 1A antagonist	P19438	92	0.9948	0.995	Membrane receptor	NA
Tumor necrosis factor receptor superfamily member 5 antagonist	P25942	3	1.000	1.000	Membrane receptor	NA
Type-1 angiotensin II receptor agonist	P30556	132	0.9994	0.999	Membrane receptor	Family A G protein-coupled receptor
Type-1 angiotensin II receptor antagonist	P30556	446	0.9949	0.995	Membrane receptor	Family A G protein-coupled receptor
Type-2 angiotensin II receptor antagonist	P50052	438	0.9975	0.998	Membrane receptor	Family A G protein-coupled receptor
Tyrosinase inhibitor	P14679	55	0.9946	0.995	Enzyme	Oxidoreductase
Tyrosine-protein kinase ABL1 activator	P00519	24	0.9987	0.999	Enzyme	Kinase
Tyrosine-protein kinase ABL1 allosteric inhibitor	P00519	6	1.000	1.000	Enzyme	Kinase
Tyrosine-protein kinase ABL1 inhibitor	P00519	2024	0.9716	0.972	Enzyme	Kinase
Tyrosine-protein kinase ABL2 inhibitor	P42684	24	0.8368	0.794	Enzyme	Kinase
Tyrosine-protein kinase Blk inhibitor	P51451	76	0.9449	0.945	Enzyme	Kinase
Tyrosine-protein kinase BTK inhibitor	Q06187	2229	0.9918	0.992	Enzyme	Kinase
Tyrosine-protein kinase CSK inhibitor	P41240	84	0.9158	0.914	Enzyme	Kinase
Tyrosine-protein kinase Fer inhibitor	P16591	26	0.8765	0.882	Enzyme	Kinase
Tyrosine-protein kinase Fes/Fps inhibitor	P07332	19	0.8434	0.841	Enzyme	Kinase
Tyrosine-protein kinase Fgr inhibitor	P09769	50	0.8585	0.859	Enzyme	Kinase
Tyrosine-protein kinase Fyn inhibitor	P06241	197	0.9022	0.902	Enzyme	Kinase
Tyrosine-protein kinase HCK inhibitor	P08631	337	0.9852	0.985	Enzyme	Kinase
Tyrosine-protein kinase ITK/TSK activator	Q08881	22	1.000	1.000	Enzyme	Kinase
Tyrosine-protein kinase ITK/TSK inhibitor	Q08881	870	0.9934	0.993	Enzyme	Kinase

Tyrosine-protein kinase JAK1 inhibitor	P23458	3160	0.992	0.992	Enzyme	Kinase
Tyrosine-protein kinase JAK2 inhibitor	O60674	4429	0.9877	0.988	Enzyme	Kinase
Tyrosine-protein kinase JAK3 inhibitor	P52333	2456	0.9867	0.987	Enzyme	Kinase
Tyrosine-protein kinase Lck inhibitor	P06239	1531	0.9688	0.969	Enzyme	Kinase
Tyrosine-protein kinase Lyn inhibitor	P07948	283	0.9603	0.959	Enzyme	Kinase
Tyrosine-protein kinase Mer inhibitor	Q12866	242	0.9727	0.973	Enzyme	Kinase
Tyrosine-protein kinase receptor Tie-1 inhibitor	P35590	4	0.9999	1.000	Enzyme	Kinase
Tyrosine-protein kinase receptor TYRO3 inhibitor	Q06418	245	0.9877	0.988	Enzyme	Kinase
Tyrosine-protein kinase receptor UFO inhibitor	P30530	386	0.9827	0.983	Enzyme	Kinase
Tyrosine-protein kinase Srms inhibitor	Q9H3Y6	9	0.8399	0.844	Enzyme	Kinase
Tyrosine-protein kinase SYK inhibitor	P43405	2376	0.9892	0.989	Enzyme	Kinase
Tyrosine-protein kinase Tec inhibitor	P42680	62	0.9623	0.962	Enzyme	Kinase
Tyrosine-protein kinase TXK inhibitor	P42681	67	0.9671	0.967	Enzyme	Kinase
Tyrosine-protein kinase Yes inhibitor	P07947	117	0.945	0.944	Enzyme	Kinase
Tyrosine-protein kinase ZAP-70 inhibitor	P43403	177	0.9712	0.97	Enzyme	Kinase
Tyrosine-protein phosphatase non-receptor type 1 inhibitor	P18031	1948	0.9837	0.983	Enzyme	Phosphatase
Tyrosine-protein phosphatase non-receptor type 11 inhibitor	Q06124	289	0.9828	0.982	Enzyme	Phosphatase
Tyrosine-protein phosphatase non-receptor type 12 inhibitor	Q05209	47	0.9938	0.992	Enzyme	Phosphatase
Tyrosine-protein phosphatase non-receptor type 13 inhibitor	Q12923	17	0.9998	1.000	Enzyme	Phosphatase
Tyrosine-protein phosphatase non-receptor type 2 inhibitor	P17706	376	0.9906	0.99	Enzyme	Phosphatase
Tyrosine-protein phosphatase non-receptor type 22 inhibitor	Q9Y2R2	126	0.9728	0.972	Enzyme	Phosphatase
Tyrosine-protein phosphatase non-receptor type 5 inhibitor	P54829	83	0.9415	0.938	Enzyme	Phosphatase
Tyrosine-protein phosphatase non-receptor type 6 inhibitor	P29350	83	0.9723	0.971	Enzyme	Phosphatase
Tyrosine-protein phosphatase non-receptor type 7 inhibitor	P35236	145	0.9412	0.939	Enzyme	Phosphatase
Tyrosine-protein phosphatase non-receptor type 9 inhibitor	P43378	19	1.000	1.000	Enzyme	Phosphatase
Tyrosine--tRNA ligase, cytoplasmic inhibitor	P54577	16	1.000	1.000	Enzyme	Ligase
Tyrosyl-DNA phosphodiesterase 1 inhibitor	Q9NUW8	3166	0.8903	0.889	Enzyme	Phosphodiesterase
Tyrosyl-DNA phosphodiesterase 2 inhibitor	O95551	181	0.9843	0.985	Enzyme	Phosphodiesterase
U5 small nuclear ribonucleoprotein 200 kDa helicase inhibitor	O75643	24	1.000	1.000	Enzyme	Hydrolase
Ubiquitin carboxyl-terminal hydrolase 2 inhibitor	O75604	3144	0.9062	0.906	Enzyme	Protease
Ubiquitin carboxyl-terminal hydrolase 25 inhibitor	Q9UHP3	171	1.000	1.000	Enzyme	Hydrolase
Ubiquitin carboxyl-terminal hydrolase 28 inhibitor	Q96RU2	204	0.9972	0.997	Enzyme	Protease

Ubiquitin carboxyl-terminal hydrolase 30 inhibitor	Q70CQ3	551	0.9999	1.000	Enzyme	Hydrolase
Ubiquitin carboxyl-terminal hydrolase 47 inhibitor	Q96K76	12	1.000	1.000	Enzyme	Protease
Ubiquitin carboxyl-terminal hydrolase 7 inhibitor	Q93009	122	0.9922	0.992	Enzyme	Protease
Ubiquitin carboxyl-terminal hydrolase isozyme L1 inhibitor	P09936	15	0.9674	0.968	Enzyme	Protease
Ubiquitin-40S ribosomal protein S27a inhibitor	P62979	25	1.000	1.000	Unclassified protein	NA
Ubiquitin-conjugating enzyme E2 N inhibitor	P61088	116	0.9193	0.903	Enzyme	Aminoacyltransferase
Ubiquitin-like domain-containing CTD phosphatase 1 inhibitor	Q8WVY7	8	0.9996	1.000	Enzyme	Phosphatase
Ubiquitin-like modifier-activating enzyme ATG7 inhibitor	O95352	189	1.000	1.000	Enzyme	NA
UDP-glucuronosyltransferase 1A1 inhibitor	P22309	10	0.9303	0.924	Enzyme	Transferase
UDP-glucuronosyltransferase 2B7 inhibitor	P16662	25	0.9977	0.998	Enzyme	Transferase
UDP-N-acetylglucosamine--peptide N-acetylglucosaminyltransferase 110 kDa subunit inhibitor	O15294	17	0.9896	0.991	Enzyme	NA
Uracil nucleotide/cysteinyl leukotriene receptor agonist	Q13304	34	0.9999	1.000	Membrane receptor	Family A G protein-coupled receptor
Uracil nucleotide/cysteinyl leukotriene receptor antagonist	Q13304	9	0.9349	0.941	Membrane receptor	Family A G protein-coupled receptor
Uracil-DNA glycosylase inhibitor	P13051	3	0.9992	0.999	Enzyme	Hydrolase
Uridine 5'-monophosphate synthase inhibitor	P11172	4	1.000	1.000	Enzyme	NA
Uridine-cytidine kinase 2 inhibitor	Q9BZX2	3	0.9942	0.994	Enzyme	Transferase
Urokinase plasminogen activator surface receptor antagonist	Q03405	31	0.9993	0.999	Membrane receptor	NA
Urokinase-type plasminogen activator inhibitor	P00749	1012	0.9945	0.995	Enzyme	Protease
Urotensin-2 receptor agonist	Q9UKP6	103	0.9973	0.997	Membrane receptor	Family A G protein-coupled receptor
Urotensin-2 receptor antagonist	Q9UKP6	152	0.9933	0.993	Membrane receptor	Family A G protein-coupled receptor
Valine--tRNA ligase inhibitor	P26640	3	1.000	1.000	Enzyme	Ligase
Vascular cell adhesion protein 1 inhibitor	P19320	50	0.9992	0.999	Adhesion	NA
Vascular endothelial growth factor receptor 1 antagonist	P17948	894	0.9812	0.98	Enzyme	Kinase
Vascular endothelial growth factor receptor 2 antagonist	P35968	7648	0.9735	0.973	Enzyme	Kinase
Vascular endothelial growth factor receptor 3 antagonist	P35916	482	0.9592	0.96	Enzyme	Kinase
Vasoactive intestinal polypeptide receptor 1 agonist	P32241	13	1.000	1.000	Membrane receptor	Family B G protein-coupled receptor
Vasoactive intestinal polypeptide receptor 1 antagonist	P32241	3	0.9999	1.000	Membrane receptor	Family B G protein-coupled receptor
Vasoactive intestinal polypeptide receptor 2 agonist	P41587	27	0.9999	1.000	Membrane receptor	Family B G protein-coupled receptor

Vasoactive intestinal polypeptide receptor 2 antagonist	P41587	21	1.000	1.000	Membrane receptor	Family B G protein-coupled receptor
Vasopressin V1a receptor agonist	P37288	150	0.9914	0.992	Membrane receptor	Family A G protein-coupled receptor
Vasopressin V1a receptor antagonist	P37288	491	0.9964	0.996	Membrane receptor	Family A G protein-coupled receptor
Vasopressin V1b receptor agonist	P47901	196	0.9999	1.000	Membrane receptor	Family A G protein-coupled receptor
Vasopressin V1b receptor antagonist	P47901	219	0.9952	0.995	Membrane receptor	Family A G protein-coupled receptor
Vasopressin V2 receptor agonist	P30518	299	0.9984	0.998	Membrane receptor	Family A G protein-coupled receptor
Vasopressin V2 receptor antagonist	P30518	257	0.9859	0.985	Membrane receptor	Family A G protein-coupled receptor
Vesicular acetylcholine transporter inhibitor	Q16572	102	0.9997	1.000	Transporter	Electrochemical transporter
Vitamin D3 receptor agonist	P11473	124	0.9968	0.997	Transcription factor	Nuclear receptor
Vitamin D3 receptor antagonist	P11473	180	0.99	0.99	Transcription factor	Nuclear receptor
Vitamin K epoxide reductase complex subunit 1 inhibitor	Q9BQB6	5	0.9976	0.998	Enzyme	Oxidoreductase
Vitamin K-dependent protein C inhibitor	P04070	106	0.9934	0.993	Enzyme	Protease
Voltage-dependent calcium channel gamma-8 subunit blocker	Q8WXS5	20	0.9618	0.961	Auxiliary transport protein	Calcium channel auxiliary subunit gamma family
Voltage-dependent calcium channel subunit alpha-2/delta-1 blocker	P54289	185	0.9984	0.998	Auxiliary transport protein	Calcium channel auxiliary subunit alpha2delta family
Voltage-dependent calcium channel subunit alpha-2/delta-2 blocker	Q9NY47	25	1.000	1.000	Auxiliary transport protein	Calcium channel auxiliary subunit alpha2delta family
Voltage-dependent L-type calcium channel subunit alpha-1C blocker	Q13936	235	0.9434	0.945	Ion channel	Voltage-gated ion channel
Voltage-dependent L-type calcium channel subunit alpha-1D blocker	Q01668	5	0.9994	0.999	Ion channel	Voltage-gated ion channel
Voltage-dependent L-type calcium channel subunit alpha-1S blocker	Q13698	5	1.000	1.000	Ion channel	Voltage-gated ion channel
Voltage-dependent N-type calcium channel subunit alpha-1B blocker	Q00975	323	0.9906	0.991	Ion channel	Voltage-gated ion channel
Voltage-dependent P/Q-type calcium channel subunit alpha-1A blocker	O00555	20	0.8895	0.89	Ion channel	Voltage-gated ion channel
Voltage-dependent T-type calcium channel subunit alpha-1G blocker	O43497	494	0.9965	0.995	Ion channel	Voltage-gated ion channel

Voltage-dependent T-type calcium channel subunit alpha-1H blocker	O95180	370	0.9967	0.997	Ion channel	Voltage-gated ion channel
Voltage-dependent T-type calcium channel subunit alpha-1I blocker	Q9P0X4	239	0.9975	0.998	Ion channel	Voltage-gated ion channel
von Hippel-Lindau disease tumor suppressor inhibitor	P40337	15	0.9999	1.000	Unclassified protein	NA
V-type proton ATPase subunit B, kidney isoform inhibitor	P15313	5	1.000	1.000	Transporter; Enzyme	Primary active transporter; Hydrolase
WD repeat-containing protein 48 inhibitor	Q8TAF3	71	0.9722	0.968	Epigenetic regulator	Reader
WD repeat-containing protein 5 inhibitor	P61964	168	0.9992	0.999	Other nuclear protein	NA
Wee1-like protein kinase inhibitor	P30291	298	0.9947	0.995	Enzyme	Kinase
Xaa-Pro aminopeptidase 1 inhibitor	Q9NQW7	4	0.9999	1.000	Enzyme	Protease
Xaa-Pro aminopeptidase 2 inhibitor	O43895	8	1.000	1.000	Enzyme	Protease
Xaa-Pro dipeptidase inhibitor	P12955	5	0.9925	0.993	Enzyme	Protease
Xanthine dehydrogenase/oxidase inhibitor	P47989	250	0.9965	0.997	Enzyme	Oxidoreductase
Zinc finger protein GLI1 inhibitor	P08151	10	0.9649	0.966	Transcription factor	NA
Zinc finger protein GLI2 inhibitor	P10070	5	0.9884	0.989	Transcription factor	NA

Number – number of active compounds; **LOO CV** – leave-one-out cross-validation; **20-Fold CV** – 20-fold cross-validation.
