# Supplementary material for: CLC-Pred 2.0: A Freely Available Web Application for In Silico Prediction of Human Cell Line Cytotoxicity and Molecular Mechanisms of Action for Druglike Compounds
Source: Int J Mol Sci. 2023 Jan 14;24(2):1689. doi: 10.3390/ijms24021689 (PMC9861947; doi:10.3390/ijms24021689)
Supplement: Supplementary file 1 [file ijms-24-01689-s001.zip › Table S1.pdf]

Table S1: The information related with description of the training set based on ChEMBL and PubChem data and accuracy of cytotoxicity prediction for appropriate cell lines.

ActivityType	Cell line full name (Disease)	Tissue/Organ	Type	Number	IAP,LOO	IAP, 20-Fold
143B	Osteosarcoma	Bone	Osteosarcoma	45	0.931	0.920
184B5	Epithelial mammary gland cells	Breast	Normal	3	0.999	0.999
1A9	Ovarian adenocarcinoma	Ovarium	Adenocarcinoma	93	0.991	0.990
1A9/ptx-10	Ovarian adenocarcinoma	Ovarium	Adenocarcinoma	36	0.991	0.991
1A9/ptx-22	Ovarian adenocarcinoma	Ovarium	Adenocarcinoma	24	0.995	0.994
2008	Ovarian adenocarcinoma	Ovarium	Adenocarcinoma	4	0.863	0.866
4T1	Epithelial mammary gland cells	Breast	Normal	37	0.887	0.885
518A2	Melanoma	Derived from metastatic site: Not specified.	Melanoma	38	0.999	0.999
5637	Urothelial bladder carcinoma	Urinary tract	Carcinoma	167	0.839	0.838
786-0	Renal carcinoma	Kidney	Carcinoma	981	0.877	0.873
833K	Testicular embryonal carcinoma	Testicle	Carcinoma	16	0.992	0.993
8505C	Thyroid gland undifferentiated (anaplastic) carcinoma	Thyroid	Carcinoma	129	0.832	0.833
A172	Glioblastoma	Brain	Glioblastoma	6	0.807	0.810
A-375	Malignant melanoma	Skin	Melanoma	1407	0.931	0.930
A-427	Lung carcinoma	Lung	Carcinoma	210	0.872	0.866
A-431	Epidermoid carcinoma	Skin	Carcinoma	1547	0.937	0.935
A121	Ovarian carcinoma	Ovarium	Carcinoma	80	1.000	1.000
A2058	Melanoma	Skin	Melanoma	109	0.859	0.851
A2780	Ovarian carcinoma	Ovarium	Carcinoma	2930	0.931	0.930
A2780/T	Putative Paclitaxel-resistant ovarian endometrioid adenocarcinoma	Ovarium	Adenocarcinoma	29	0.959	0.959
A2780S	Ovarian endometrioid adenocarcinoma	Ovarium	Adenocarcinoma	13	0.851	0.855
A2780cisR	Cisplatin-resistant ovarian carcinoma	Ovarium	Carcinoma	3	0.838	0.843
A498	Renal carcinoma	Kidney	Carcinoma	1046	0.892	0.891
A549	Lung carcinoma	Lung	Carcinoma	15040	0.856	0.856
A549/CDDP	Putative Taxol-resistant lung adenocarcinoma	Lung	Adenocarcinoma	23	0.999	0.999
A549/TR	Putative Cisplatin-resistant lung adenocarcinoma	Lung	Adenocarcinoma	26	0.959	0.959
ACHN	Papillary renal carcinoma	Kidney	Carcinoma	1582	0.901	0.899
ADR5000	Childhood T acute lymphoblastic leukemia	Blood	Leukemia	11	0.911	0.913
AG1523	Fibroblast	Fibroblast	Normal	25	0.962	0.961
AGS	Gastric adenocarcinoma	Stomach	Adenocarcinoma	310	0.875	0.872
ARPE-19	Retinal pigmented epithelium	Eye; Retina	Normal	48	1.000	1.000
ASPC1	Pancreatic ductal adenocarcinoma	Pancreas	Adenocarcinoma	234	0.901	0.894
ATH-8	HTLV-I-infected human T-cell line	Peripheral blood	Normal	3	0.964	0.965
BE	Colon adenocarcinoma	Colon	Adenocarcinoma	17	0.988	0.988
BE-NQ	Colon adenocarcinoma	Colon	Adenocarcinoma	15	1.000	1.000
BEAS-2B	Epithelial cells	bronchial epithelium	Normal	9	1.000	0.999
BGC-823	Stomach adenocarcinoma	Stomach	Adenocarcinoma	92	0.969	0.969
BHK-21	Fibroblast	kidney	Normal	15	0.905	0.909
BJ	Foreskin fibroblast	Foreskin	Normal	150	0.955	0.955
BT-474	Breast ductal carcinoma	Breast	Carcinoma	625	0.941	0.941
BT-549	Breast ductal carcinoma	Breast	Carcinoma	839	0.882	0.880

BV-2	Murine microglial cell line	Microglial cells	Normal	44	0.962	0.961
BXPC-3	Pancreatic adenocarcinoma	Pancreas	Adenocarcinoma	485	0.917	0.916
Bcap37	Breast adenocarcinoma	Breast	Adenocarcinoma	109	0.979	0.979
Bel-7402	Hepatoma	Liver	Hepatoma	133	0.970	0.967
Bel7402/5-FU	Human papillomavirus-related endocervical adenocarcinoma	Uterus; Cervix	Adenocarcinoma	28	0.966	0.966
C180-13S	Ovarian carcinoma	Ovarium	Carcinoma	11	0.999	0.999
C6	Glioma	Brain	Glioma	8	0.980	0.761
C8166	Leukemic T-cells	Blood	Leukemia	105	0.839	0.841
CA46	Burkitts Lymphoma	Blood	Lymphoma	98	0.810	0.813
CAKI-1	Kidney carcinoma	Kidney	Carcinoma	979	0.880	0.877
CAKI-2	Kidney carcinoma	Kidney	Carcinoma	5	0.829	0.834
CAL-27	Squamous Cell Carcinoma	Tongue	Carcinoma	113	0.854	0.859
CAL-51	Breast carcinoma	Breast	Carcinoma	117	0.824	0.821
CAPAN-1	Pancreas Adenocarcinoma	Pancreas	Adenocarcinoma	149	0.889	0.892
CCD-18Co	Fibroblasts	Colon	Normal	261	0.994	0.994
CCRF-CEM	Childhood T acute lymphoblastic leukemia	Blood	Leukemia	3625	0.913	0.912
CCRF-CEM/VCR-1000	T-cell leukaemia	Blood	Leukemia	62	0.989	0.990
CCRF-HSB-2	Childhood T acute lymphoblastic leukemia	Blood	Leukemia	23	0.941	0.942
CCRF-SB	Childhood T acute lymphoblastic leukemia	Blood	Leukemia	91	0.967	0.967
CEM-DNR	Childhood T acute lymphoblastic leukemia	Blood	Leukemia	12	0.951	0.952
CEM-SS	Childhood T acute lymphoblastic leukemia	Blood	Leukemia	142	0.984	0.985
CEM-c113	Childhood T acute lymphoblastic leukemia	Blood	Leukemia	4	1.000	1.000
CEM/C2	Camptothecin-resistant CEM	Blood	Leukemia	17	0.934	0.937
CFPAC-1	Pancreatic carcinoma	Pancreas	Carcinoma	84	0.905	0.906
CL1-0	Lung adenocarcinoma	Lung	Adenocarcinoma	4	0.830	0.832
CL97	Lung adenocarcinoma	Lung	Adenocarcinoma	6	0.872	0.880
CNE	Hybrid cell line	Hybrid	Normal	4	0.992	0.992
CNE-2	Hybrid cell line	Hybrid	Normal	4	0.990	0.991
CNE2Z	Hybrid cell line	Hybrid	Normal	3	1.000	1.000
COLO-205	Colorectal Adenocarcinoma	Colon	Adenocarcinoma	1700	0.901	0.899
COLO-320	Colon adenocarcinoma	Colon	Adenocarcinoma	10	0.999	0.998
COLO-320DM	Colorectal Adenocarcinoma	Colon	Adenocarcinoma	64	0.994	0.980
COLO357	Pancreatic adenosquamous carcinoma	Pancreas	Carcinoma	11	1.000	1.000
COR-L23	Lung large cell carcinoma	Lung	Carcinoma	115	0.834	0.836
CPT30	Nasopharyngeal carcinoma	Head and neck	Carcinoma	7	1.000	1.000
CRL-7065	Fibroblast	Skin	Normal	9	0.934	0.935
CT26	Mouse colon adenocarcinoma	Colon	Adenocarcinoma	15	0.869	0.871
CWR22R	Prostate carcinoma epithelial cell line	Prostate	Carcinoma	303	0.911	0.907
Cac-2	Carcinoma Adenoide Cistico 2	Adenoide	Carcinoma	345	0.925	0.922
Caco-2	Colon adenocarcinoma	Colon	Adenocarcinoma	47	0.898	0.899
Calu-1	Epidermoid Carcinoma	Lung	Carcinoma	85	0.851	0.853
Caov-3	Ovary Adenocarcinoma	Ovary	Adenocarcinoma	7	0.890	0.892
Capan-2	Pancreas Adenocarcinoma	Pancreas	Adenocarcinoma	13	0.895	0.893
Cav-3	Transformed cell line	Kidney	Normal	57	0.936	0.939

Col2	Colon carcinoma	Colon	Carcinoma	3	1.000	1.000
CuFi-1	Cystic Fibrosis	Lung; Bronchus; Epithelium	Normal	3	1.000	1.000
D54	Glioblastoma	Brain	Glioblastoma	4	0.995	0.995
DAN-G	Human pancreas adenocarcinoma cell line	Pancreas	Adenocarcinoma	54	0.962	0.955
DLD-1	Colon adenocarcinoma	Colon	Adenocarcinoma	724	0.949	0.949
DMS-114	Lung carcinoma	Lung	Carcinoma	124	0.834	0.837
DO4	Melanoma	Skin	Melanoma	6	0.999	0.999
DU-145	Prostate carcinoma	Prostate	Carcinoma	4562	0.896	0.896
DU-4475	Breast Carcinoma	Breast	Carcinoma	125	0.802	0.800
Daoy	Desmoplastic Cerebellar Medulloblastoma	Brain	Medulloblastoma	21	0.908	0.909
Daudi	Burkitts Lymphoma	Blood	Lymphoma	31	0.990	0.990
Detroit562	Pharyngeal Carcinoma	Pharynx	Carcinoma	5	0.989	0.989
EA.hy 926	Somatic cell hybrid	Hybrid	Normal	20	0.968	0.968
EBC-1	Lung squamous cell carcinoma	Lung	Carcinoma	19	0.944	0.945
ECa-109	Esophageal squamous cell carcinoma	Esophagus	Carcinoma	94	0.961	0.962
EJ	Endometrial adenocarcinoma	Endometrium	Adenocarcinoma	9	1.000	1.000
EKVX	Non-small cell lung carcinoma	Lung	Carcinoma	522	0.841	0.841
EOL1	Chronic eosinophilic leukemia, not otherwise specified	Blood	Leukemia	167	0.861	0.859
FHC	Epithelial cell	Large intestine; Colon	Normal	10	0.820	0.821
FaDu	Hypopharyngeal squamous carcinoma	Upper aerodigestive tract	Carcinoma	254	0.902	0.901
G-361	Melanoma	Skin	Melanoma	191	0.890	0.882
G55T2	Anaplastic astrocytoma	Brain	Astrocytoma	11	1.000	1.000
GC3/Cl	Colorectal carcinoma	Colon	Carcinoma	5	1.000	1.000
GC3/M	Colorectal carcinoma	Colon	Carcinoma	12	1.000	1.000
GC3/MTK-	Colorectal carcinoma	Colon	Carcinoma	13	1.000	1.000
GES1	Gastric epithelial cell line	Stomach	Normal	33	0.963	0.964
GHOSTCXCR4	Osteosarcoma	Bone	Sarcoma	3	1.000	1.000
GIST430	Gastrointestinal stromal tumor	Intestine	Carcinoma	5	0.820	0.823
GIST48	Gastrointestinal stromal tumor	Intestine	Carcinoma	6	0.805	0.806
GIST882	Gastrointestinal stromal tumor	Intestine	Carcinoma	6	0.824	0.828
GISTT1	Gastrointestinal stromal tumor	Intestine	Carcinoma	32	0.942	0.941
GLC4	Lung small cell carcinoma	Lung	Carcinoma	10	1.000	1.000
GM892A	Immortal human peripheral vein-derived B cell line cell	Blood	Normal	4	1.000	1.000
Granta-519	Mantle cell lymphoma	Blood	Lymphoma	23	0.993	0.993
H2981	Lung carcinoma	Lung	Carcinoma	11	0.989	0.989
H322	Lung carcinoma	Lung	Carcinoma	6	1.000	1.000
H9	T-lymphoid	Haematopoietic and lymphoid tissue	Leukemia	123	0.872	0.873
H9c2	Rat DB1X heart myoblast cell line	Heart	Myoblast	9	0.897	0.787
HA22T	Hepatocellular carcinoma	Liver	Carcinoma	21	0.946	0.944
HBL	Melanoma	Skin	Melanoma	10	0.939	0.939
HBL-100	Human mammary gland breast carcinoma	Breast	Carcinoma	21	0.957	0.957
HCC1806	Acantholytic Squamous Cell Carcinoma	Breast	Carcinoma	108	0.824	0.820
HCC1937	Breast Carcinoma	Breast	Carcinoma	78	0.806	0.791
HCC1954	Breast Carcinoma	Breast	Carcinoma	94	0.829	0.830

HCC2998	Colon adenocarcinoma	Colon	Adenocarcinoma	708	0.872	0.871
HCC78	Lung adenocarcinoma	Lung	Adenocarcinoma	65	0.989	0.989
HCC827	Lung Adenocarcinoma	Lung	Adenocarcinoma	113	0.985	0.985
HCT-116	Colon carcinoma	Colon	Carcinoma	10992	0.890	0.889
HCT-116/VM46	Colon carcinoma	Colon	Carcinoma	104	0.998	0.998
HCT-15	Colon adenocarcinoma	Colon	Adenocarcinoma	2078	0.910	0.909
HCT-8	Ileocecal adenocarcinoma	Large intestine	Adenocarcinoma	681	0.960	0.958
HEC-1B	Adenocarcinoma	Uterus Endometrium	Adenocarcinoma	11	1.000	1.000
HEK-293T	Renal epithelial line	Kidney	Normal	58	0.967	0.967
HEK293	Embryonic kidney fibroblast	Kidney	Normal	1110	0.898	0.898
HEL	Erythroleukemia	Blood	Leukemia	302	0.903	0.900
HEL299	Fibroblasts	Lung	Normal	4	0.899	0.904
HET-1A	Epithelial cell	Esophagus	Normal	4	0.998	0.998
HEY	Ovarian carcinoma	Ovarium	Carcinoma	34	0.967	0.968
HFF	Foreskin fibroblast	Skin	Normal	27	0.955	0.951
HFL1	Human foetal lung fibroblast	Lung	Normal	309	0.996	0.996
HGC-27	Gastric carcinoma	Stomach	Carcinoma	148	0.830	0.821
HK-2	Papilloma	Kidney	Normal	10	0.879	0.881
HL-60	Promyeloblast leukemia	Haematopoietic and lymphoid tissue	Leukemia	5486	0.881	0.880
HL-60(TB)	Adult acute myeloid leukemia	Blood	Leukemia	53	0.950	0.949
HMEC-1	Dermal microvascular endothelial cell	Skin; Dermis; Endothelium	Normal	42	0.981	0.981
HN5	Squamous cell carcinoma	Skin	Carcinoma	75	0.987	0.987
HONE1	Nasopharyngeal carcinoma	Head and neck	Carcinoma	5	1.000	1.000
HOP-18	Non-small cell lung carcinoma	Lung	Carcinoma	9	0.933	0.862
HOP-62	Non-small cell lung carcinoma	Lung	Carcinoma	1318	0.898	0.897
HOP-92	Non-small cell lung carcinoma	Lung	Carcinoma	679	0.862	0.856
HOS	Osteosarcoma	Bone	Sarcoma	161	0.863	0.852
HOS-TE85	Osteosarcoma	Bone	Sarcoma	13	0.844	0.845
HPAC	Pancreatic adenocarcinoma	Pancreas	Adenocarcinoma	28	0.932	0.934
HRT-18	Colon adenocarcinoma	Colon	Adenocarcinoma	4	1.000	1.000
HS27	Fibroblast	Skin	Normal	41	0.970	0.968
HT	Lymphoma	Haematopoietic and lymphoid tissue	Leukemia	121	0.857	0.860
HT-1080	Fibrosarcoma	Soft tissue	Sarcoma	534	0.918	0.916
HT-29	Colon adenocarcinoma	Colon	Adenocarcinoma	7636	0.888	0.887
HT1197	Carcinoma	Urinary bladder	Carcinoma	59	0.815	0.815
HUVEC	Umbilical vein endothelial cell	Endothelium	Normal	131	0.897	0.892
HaCaT	Keratinocyte	Skin	Normal	375	0.967	0.967
HeLa	Cervical adenocarcinoma	Cervix	Adenocarcinoma	8431	0.885	0.884
HeLa S3	Adenocarcinoma	Uterus; Cervix	Adenocarcinoma	23	0.974	0.975
Hep 3B2	Childhood hepatocellular carcinoma	Liver	Carcinoma	135	0.957	0.956
Hep293TT	Hepatoblastoma	Liver	Hepatoblastoma	13	0.987	0.987
HepG2	Hepatoblastoma	Liver	Hepatoblastoma	7802	0.873	0.871
HepG 22.2.15	Hepatoblastoma	Liver	Hepatoblastoma	12	0.853	0.854
HepG2-CD81	Hepatoblastoma (HepG2) cells expressing CD81	Liver	Hepatoblastoma	5768	0.932	0.932
Hs-578T	Invasive ductal breast carcinoma	Breast	Carcinoma	434	0.869	0.865

Hs683	Glioma	Brain	Glioma	47	0.959	0.958
Hs766	Pancreatic adenocarcinoma	Liver	Carcinoma	11	1.000	1.000
HuCC-A1	Intrahepatic cholangiocarcinoma	Liver	Carcinoma	16	0.881	0.883
HuP-T3	Pancreatic adenocarcinoma	Pancreas	Adenocarcinoma	62	0.868	0.867
HuT78	T-lymphoma	Haematopoietic and lymphoid tissue	Leukemia	40	0.978	0.977
Huh-7	Hepatocellular carcinoma	Liver	Carcinoma	555	0.920	0.919
IGROV-1	Ovarian adenocarcinoma	Ovarium	Adenocarcinoma	1116	0.891	0.888
IMR-32	Neuroblastoma	Nervous system	Neuroblastoma	188	0.951	0.952
IMR-90	Embryonic lung fibroblast	Lung	Normal	18	0.879	0.869
Ishikawa	Uterine carcinoma	Uterus	Carcinoma	111	0.965	0.965
JAM	Ovarian cystadenocarcinoma	Ovarium	Adenocarcinoma	5	0.999	0.999
JJN-3	Plasma cell myeloma	Bone Marrow	Myeloma	17	1.000	1.000
JY	EBV-positive lymphoblastoid B cell line	Blood	Normal	13	1.000	1.000
JeKo-1	Lymphoma	Blood	Lymphoma	74	0.948	0.950
Jurkat	Acute leukemic T-cells	Blood	Leukemia	218	0.946	0.947
Jurkat E6.1	Acute T cell leukemia	Blood	Leukemia	5	1.000	1.000
K-562R	Blast phase chronic myelogenous leukemia, BCR-ABL1 positive	Haematopoietic and lymphoid tissue	Leukemia	14	0.869	0.870
K562	Erythroleukemia	Haematopoietic and lymphoid tissue	Leukemia	6204	0.892	0.892
K562/Adr	Blast phase chronic myelogenous leukemia, BCR-ABL1 positive	Haematopoietic and lymphoid tissue	Leukemia	13	0.972	0.974
KARPAS-299	Anaplastic large cell lymphoma	Haematopoietic and lymphoid tissue	Leukemia	291	0.939	0.940
KARPAS-422	Diffuse large B-cell lymphoma germinal center B-cell type	Haematopoietic and lymphoid tissue	Leukemia	128	0.875	0.875
KB	Human papillomavirus-related squamous cell carcinoma	Uterus; cervix	Carcinoma	203	0.966	0.967
KB 3-1	Human papillomavirus-related endocervical adenocarcinoma	Uterus	Adenocarcinoma	22	0.960	0.962
KB/VJ300	Human papillomavirus-related endocervical adenocarcinoma	Uterus	Adenocarcinoma	23	0.989	0.989
KBM5	Chronic myelogenous leukemia, BCR-ABL1 positive	Blood	Leukemia	6	0.931	0.932
KETR3	Renal carcinoma	Kidney	Carcinoma	43	0.981	0.980
KG-1	Acute myelogenous leukemia	Blood	Leukemia	253	0.901	0.897
KG-1a	Acute myelogenous leukemia	Blood	Leukemia	17	0.999	0.999
KK-47	Bladder carcinoma	Bladder	Carcinoma	3	0.887	0.889
KKLS	Gastric adenocarcinoma	Stomach	Adenocarcinoma	4	1.000	1.000
KM-20L2	Colon adenocarcinoma	Colon	Adenocarcinoma	4	1.000	1.000
KM12	Colon adenocarcinoma	Colon	Adenocarcinoma	1049	0.875	0.873
KM3/BTZ	Plasma cell myeloma	Bone Marrow	Myeloma	6	0.994	0.994
KMS-12-BM	Plasma cell myeloma	Bone Marrow	Myeloma	4	0.998	0.998
KOPTK1	Childhood T acute lymphoblastic leukemia	Blood	Leukemia	5	0.808	0.809
KYSE-520	Esophageal squamous cell carcinoma	Esophagus	Carcinoma	83	0.858	0.857
Kasumi-1	Acute myeloblastic leukemia	Blood	Leukemia	116	0.816	0.806
L02	Human papillomavirus-related endocervical adenocarcinoma	Uterus	Adenocarcinoma	193	0.933	0.934
L132	Human papillomavirus-related endocervical adenocarcinoma	Uterus	Adenocarcinoma	4	1.000	1.000
L2987	Lung adenocarcinoma	Lung	Adenocarcinoma	11	0.978	0.978
L3.6pl	Pancreatic adenosquamous carcinoma	Pancreas	Carcinoma	3	0.976	0.977
LAPC4	Prostate carcinoma	Prostate	Carcinoma	16	1.000	1.000
LNCaP	Prostate carcinoma	Prostate	Carcinoma	1665	0.947	0.947

LNCaP C4-2	Prostate carcinoma	Prostate	Carcinoma	4	0.996	0.997
LOXIMVI	Amelanotic melanoma	Skin	Melanoma	1010	0.877	0.876
LS174T	Colon adencocarcinoma	Colon	Adenocarcinoma	78	0.910	0.906
LXFL529	Lung non-small cell carcinoma	Lung	Carcinoma	13	0.979	0.980
LoVo	Colon adenocarcinoma	Colon	Adenocarcinoma	71	0.911	0.914
Lu1	Lung carcinoma	Lung	Carcinoma	20	0.995	0.995
M14	Melanoma	Skin	Melanoma	1203	0.894	0.893
M19-MEL	Melanoma	Skin	Melanoma	9	0.925	0.866
M21	Melanoma	Skin	Melanoma	280	0.985	0.985
M4Beu	Melanoma	Skin	Melanoma	6	1.000	1.000
MAXF401	Breast carcinoma	Breast	Carcinoma	6	0.983	0.983
MCF-10A	Human mammary epithelial cell line	Breast	Normal	71	0.943	0.943
MCF-12A	Human mammary epithelial cell line	Breast	Normal	7	0.994	0.995
MCF7	Breast carcinoma	Breast	Carcinoma	15718	0.836	0.836
MCF7R	Breast carcinoma	Breast	Carcinoma	72	0.996	0.996
MDA-MB-231	Breast adenocarcinoma	Breast	Adenocarcinoma	7364	0.881	0.880
MDA-MB-361	Breast adenocarcinoma	Breast	Adenocarcinoma	174	0.872	0.875
MDA-MB-435	Amelanotic melanoma	Breast	Melanoma	101	0.947	0.944
MDA-MB-436	Breast adenocarcinoma	Breast	Adenocarcinoma	29	0.933	0.934
MDA-MB-453	Breast adenocarcinoma	Breast	Adenocarcinoma	233	0.888	0.886
MDA-MB-468	Breast adenocarcinoma	Breast	Adenocarcinoma	1571	0.898	0.896
MEC1	Chronic lymphocytic leukemia	Blood	Leukemia	14	0.997	0.997
MES-SA	Uterine corpus sarcoma	Uterus	Sarcoma	83	0.815	0.815
MES-SA/Dx5	Uterine corpus sarcoma	Uterus	Sarcoma	46	0.940	0.940
MEXF276L	Xenograft melanoma	Skin	Melanoma	4	0.808	0.809
MGC-803	Gastric mucinous adenocarcinoma	Stomach	Adenocarcinoma	214	0.959	0.959
MIA PaCa-2	Pancreatic carcinoma	Pancreas	Carcinoma	854	0.919	0.918
MKN-28	Gastric tubular adenocarcinoma	Stomach	Adenocarcinoma	17	0.919	0.920
MKN-45	Gastric adenocarcinoma	Stomach	Adenocarcinoma	812	0.941	0.941
MKN-74	Gastric tubular adenocarcinoma	Stomach	Adenocarcinoma	49	0.856	0.860
MM1.S	Immunoglobulin A Lambda Myeloma	Blood	Myeloma	204	0.988	0.988
MM96L	Melanoma	Skin	Melanoma	64	1.000	1.000
MOLM-13	Adult acute myeloid leukemia	Blood	Leukemia	186	0.967	0.967
MOLM-14	Adult acute myeloid leukemia	Blood	Leukemia	8	0.888	0.889
MOLT-3	T-lymphoblastic leukemia	Blood	Leukemia	90	0.976	0.956
MOLT-4	Acute T-lymphoblastic leukemia	Blood	Leukemia	1490	0.885	0.885
MONO-MAC-6	Adult acute monocytic leukemia	Blood	Leukemia	92	0.807	0.810
MR49F	Prostate carcinoma	Prostate	Carcinoma	3	1.000	1.000
MRC5	Embryonic lung fibroblast	Lung	Normal	596	0.916	0.916
MSTO-211H	Biphasic Mesothelioma	Lung	Mesothelioma	121	0.878	0.880
MT2	Lymphocyte (HTLV-1 producing cell line)	Blood	Normal	101	0.963	0.963
MT4	Adult T acute lymphoblastic leukemia	Blood	Leukemia	466	0.974	0.973
MV4-11	Myeloid leukemia	Haematopoietic and lymphoid tissue	Leukemia	1832	0.947	0.947
MX1	Breast carcinoma	Breast	Carcinoma	243	0.974	0.973
Mahlavu	Hepatocellular carcinoma	Liver	Carcinoma	3	0.937	0.938

Malme-3M	Melanoma	Skin	Melanoma	712	0.880	0.877
Maver1	Mantle Cell Lymphoma	Blood	Lymphoma	31	0.930	0.931
NALM-6	Adult B acute lymphoblastic leukemia	Haematopoietic and lymphoid tissue	Leukemia	192	0.849	0.850
NAMALVA	EBV-related Burkitt lymphoma	Blood	Lymphoma	35	0.986	0.986
NB-4	Acute promyelocytic leukemia	Haematopoietic and lymphoid tissue	Leukemia	174	0.950	0.950
NCH82	Glioblastoma	Brain	Glioblastoma	6	1.000	1.000
NCI-H128	Small cell lung cancer	Lung	Carcinoma	16	0.908	0.914
NCI-H1299	Non-small cell lung carcinoma	Lung	Carcinoma	470	0.916	0.914
NCI-H157	Lung squamous cell carcinoma	Lung	Carcinoma	11	0.914	0.915
NCI-H1581	Non Small Cell Lung Cancer	Lung	Carcinoma	146	0.871	0.865
NCI-H1650	Bronchoalveolar carcinoma	Lung	Adenocarcinoma	139	0.839	0.837
NCI-H1703	Non Small Cell Lung Cancer	Lung	Carcinoma	86	0.851	0.842
NCI-H187	Small cell lung carcinoma	Lung	Carcinoma	88	0.846	0.845
NCI-H1975	Bronchoalveolar carcinoma	Lung	Carcinoma	717	0.950	0.950
NCI-H1993	Non-small cell lung cancer	Lung	Adenocarcinoma	146	0.913	0.914
NCI-H2171	Small cell lung cancer	Lung	Carcinoma	299	0.953	0.953
NCI-H2228	Non-small cell lung cancer	Lung	Adenocarcinoma	237	0.920	0.919
NCI-H226	Non-small cell lung carcinoma	Lung	Carcinoma	868	0.880	0.878
NCI-H2286	Small cell lung cancer	Lung	Adenocarcinoma	9	0.968	0.959
NCI-H23	Non-small cell lung carcinoma	Lung	Carcinoma	1192	0.883	0.882
NCI-H292	Mucoepidermoid Pulmonary Carcinoma	Lung	Carcinoma	142	0.845	0.847
NCI-H295R	Adrenal cortex carcinoma	Adrenal cortex	Carcinoma	11	0.856	0.860
NCI-H3122	Bronchioalveolar Carcinoma	Lung; Bronchiole	Carcinoma	57	0.982	0.982
NCI-H322M	Non-small cell lung carcinoma	Lung	Carcinoma	634	0.881	0.881
NCI-H358	Bronchioalveolar Carcinoma	Lung; Bronchiole	Carcinoma	109	0.805	0.782
NCI-H417	Small cell lung carcinoma	Lung	Carcinoma	22	0.985	0.985
NCI-H441	Papillary adenocarcinoma	Lung	Adenocarcinoma	69	0.813	0.802
NCI-H460	Non-small cell lung carcinoma	Lung	Carcinoma	3810	0.906	0.904
NCI-H508	Colorectal Adenocarcinoma	Large intestine; Cecum	Adenocarcinoma	10	0.991	0.991
NCI-H520	Squamous Cell Carcinoma	Lung	Carcinoma	112	0.837	0.840
NCI-H522	Non-small cell lung carcinoma	Lung	Carcinoma	1035	0.877	0.877
NCI-H647	Adenosquamous lung carcinoma	Lung	Carcinoma	10	0.889	0.890
NCI-H661	Lung carcinoma	Lung	Carcinoma	120	0.831	0.823
NCI-H69	Small cell lung carcinoma	Lung	Carcinoma	205	0.902	0.904
NCI-H727	Carcinoid	Lung; Bronchus	Carcinoma	107	0.828	0.812
NCI-H929	Plasma cell myeloma	Bone Marrow	Myeloma	35	0.972	0.974
NCI-N87	Gastric carcinoma	Stomach	Carcinoma	320	0.923	0.922
NCI/ADR-RES	High grade ovarian serous adenocarcinoma	Ovarium	Adenocarcinoma	98	0.914	0.911
NHDF	Fibroblast	Skin	Normal	30	0.953	0.955
NSCLC	Non-small cell lung carcinoma	Lung	Carcinoma	22	0.957	0.950
NT2	Embryonal carcinoma	Germ cell. fibroblast	Carcinoma	58	0.993	0.993
NUGC-3	Gastric carcinoma	Stomach	Carcinoma	288	0.926	0.927
OCI-AML-3	Adult acute myeloid leukemia	Blood	Leukemia	14	0.982	0.982
OCI-AML2	Adult acute myeloid leukemia	Blood	Leukemia	114	0.803	0.798
OCI-Ly1	Diffuse large B-cell lymphoma	Lymphoid tissue	Lymphoma	5	1.000	1.000

OCI-Ly10	Diffuse large B-cell lymphoma activated B-cell type	Lymphoid tissue	Lymphoma	3	0.919	0.922
OCI-Ly3	Diffuse large B-cell lymphoma activated B-cell type	Lymphoid tissue	Lymphoma	40	0.986	0.986
OE33	Barrett adenocarcinoma	Esophagus	Adenocarcinoma	56	0.805	0.802
OPM-2	Plasma cell myeloma	Bone Marrow	Myeloma	81	0.823	0.829
OS-RC-2	Clear cell renal cell carcinoma	Kidney	Carcinoma	117	0.812	0.808
OVCAR-3	Ovarian adenocarcinoma	Ovarium	Adenocarcinoma	1728	0.907	0.907
OVCAR-4	Ovarian adenocarcinoma	Ovarium	Adenocarcinoma	715	0.859	0.854
OVCAR-5	Ovarian adenocarcinoma	Ovarium	Adenocarcinoma	617	0.866	0.863
OVCAR-8	Ovarian adenocarcinoma	Ovarium	Adenocarcinoma	973	0.885	0.883
OVXF1023	Ovarian adenocarcinoma	Ovarium	Adenocarcinoma	3	1.000	1.000
OVXF1353	Ovarian adenocarcinoma	Ovarium	Adenocarcinoma	3	1.000	1.000
PA-1	Ovarian carcinoma	Ovarium	Carcinoma	159	0.854	0.843
PANC-1	Pancreatic carcinoma	Pancreas	Carcinoma	728	0.933	0.933
PBMC	Peripheral blood mononuclear cell	Blood	Normal	69	0.932	0.934
PC-12	Rat adrenal gland pheochromocytoma	Adrenal gland	Carcinoma	21	0.943	0.943
PC-14	Lung adenocarcinoma	Lung	Carcinoma	252	0.932	0.928
PC-3	Prostate carcinoma	Prostate	Carcinoma	5760	0.883	0.882
PC-3M	Prostate carcinoma	Prostate	Carcinoma	7	0.870	0.871
PC-9	Lung adenocarcinoma	Lung	Adenocarcinoma	130	0.951	0.952
PLC-PRF-5	Adult hepatocellular carcinoma	Liver	Carcinoma	50	0.827	0.830
PNT1A	Human prostate epithelium cell line	Prostate	Normal	8	0.881	0.881
PT-45	Pancreatic carcinoma	Pancreas	Carcinoma	20	0.938	0.939
Panc203	Pancreatic carcinoma	Pancreas	Carcinoma	6	1.000	1.000
Panc430	Pancreatic carcinoma	Pancreas	Carcinoma	8	1.000	1.000
Pfeiffer	Diffuse Large Cell Lymphoma	Blood	Lymphoma	35	0.993	0.993
QG-56	Squamous cell lung carcinoma	Lung	Carcinoma	90	1.000	1.000
QGY-7703	Hepatocellular carcinoma	Liver	Carcinoma	48	0.976	0.976
RCC4	Clear cell renal cell carcinoma	Kidney	Carcinoma	3	0.845	0.848
RD	Rhabdomyosarcoma	Muscle	Normal	104	0.828	0.832
RKO	Colon carcinoma	Colon	Carcinoma	343	0.879	0.877
RL	Non-Hodgkin's Lymphoma	Ascites	Lymphoma	43	0.833	0.836
RPMI-7951	Malignant Melanoma	Skin	Melanoma	130	0.854	0.847
RPMI-8226	Multiple myeloma	Haematopoietic and lymphoid tissue	Myeloma	1013	0.883	0.881
RS4-11	Adult B acute lymphoblastic leukemia	Bone Marrow	Leukemia	198	0.835	0.831
RWLeu4	Chronic myelogenous leukemia	Blood	Leukemia	9	1.000	1.000
RWPE-1	Prostatic epithelial cell line	Prostate	Normal	28	0.922	0.924
RXF393	Renal cell carcinoma	Kidney	Carcinoma	795	0.873	0.869
RXF944	Renal cell carcinoma	Kidney	Carcinoma	8	0.989	0.989
Raji	B-lymphoblastic cells	Haematopoietic and lymphoid tissue	Leukemia	430	0.938	0.938
Ramos	Burkitts lymhoma B-cells	Blood	Leukemia	189	0.958	0.959
Rec1	Lymphoma; Mantle Cell	Lymph node	Lymphoma	22	1.000	1.000
S1B1-20	Colon carcinoma	Colon	Carcinoma	38	1.000	1.000
SEM	Childhood B acute lymphoblastic leukemia	Blood	Leukemia	21	0.999	0.999
SF-268	Glioblastoma	Brain	Glioblastoma	1287	0.890	0.889
SF-295	Glioblastoma	Brain	Glioblastoma	1016	0.884	0.882

SF-539	Glioblastoma	Brain	Glioblastoma	1104	0.888	0.888
SGC-7901	Gastric carcinoma	Stomach	Carcinoma	128	0.963	0.963
SH-SY5Y	Bone marrow neuroblastoma	Brain	Neuroblastoma	380	0.930	0.926
SISO	Uterine cervical adenocarcinoma	Cervix	Adenocarcinoma	28	0.950	0.950
SJSA-1	Osteosarcoma	Bone	Sarcoma	398	0.940	0.940
SK-BR-3	Breast adenocarcinoma	Breast	Adenocarcinoma	1479	0.950	0.949
SK-HEP1	Hepatocellular carcinoma	Liver	Carcinoma	234	0.912	0.911
SK-LU-1	Adenocarcinoma	Lung	Carcinoma	80	0.810	0.806
SK-MEL-1	Metastatic melanoma	Skin	Melanoma	64	0.808	0.808
SK-MEL-103	Melanoma	Skin	Melanoma	3	0.999	0.999
SK-MEL-2	Melanoma	Skin	Melanoma	1048	0.885	0.885
SK-MEL-28	Melanoma	Skin	Melanoma	1160	0.879	0.879
SK-MEL-5	Melanoma	Skin	Melanoma	1037	0.903	0.902
SK-MES-1	Squamous cell lung carcinoma	Lung	Carcinoma	127	0.834	0.831
SK-N-SH	Neuroblastoma	Nervous system	Neuroblastoma	213	0.950	0.950
SK-OV-3	Ovarian carcinoma	Ovarium	Carcinoma	2424	0.896	0.894
SKM-1	Adult acute myeloid leukemia	Blood	Leukemia	66	0.917	0.918
SKW6.4	Blastoid B-Cell	Lymphocytes	Normal	40	1.000	1.000
SMMC-7721	Hepatocellular carcinoma	Liver	Carcinoma	298	0.959	0.956
SN12C	Renal carcinoma	Kidney	Carcinoma	1176	0.896	0.895
SNB-19	Astrocytoma	Brain	Astrocytoma	76	0.948	0.949
SNB-7	Glioblastoma	Brain	Glioblastoma	4	0.999	0.999
SNB-75	Glioblastoma	Nervous system	Glioblastoma	871	0.877	0.874
SNU-16	Gastric Carcinoma	Stomach	Carcinoma	65	0.850	0.847
SNU-398	Hepatocellular carcinoma	Liver	Carcinoma	74	0.970	0.970
SNU-475	Hepatocellular carcinoma	Liver	Carcinoma	22	0.819	0.814
SNU-5	Gastric Carcinoma	Stomach	Carcinoma	97	0.828	0.831
SNU-638	Gastric carcinoma	Stomach	Carcinoma	170	0.969	0.959
SPC-A4	Lung Adenocarcinoma	Lung	Adenocarcinoma	10	1.000	1.000
SQ20B	Head and neck Squamous carcinoma	Head and neck	Carcinoma	33	0.958	0.960
SR	Adult immunoblastic lymphoma	Haematopoietic and lymphoid tissue	Lymphoma	944	0.877	0.875
SU-DHL-6	Large Cell Lymphoma	Peritoneal effusion	Lymphoma	71	0.986	0.986
SU-DHL-8	Large Cell Lymphoma	Lymph node	Lymphoma	4	0.847	0.850
SUD4	Diffuse large B-cell lymphoma germinal center B-cell type	Lymphocytes	Lymphoma	14	0.811	0.814
SUM-159-PT	Breast pleomorphic carcinoma	Breast	Carcinoma	31	0.982	0.981
SUM149PT	Breast inflammatory carcinoma	Breast	Carcinoma	3	0.983	0.984
SUM185PE	Breast ductal carcinoma	Breast	Carcinoma	11	0.957	0.957
SUNE1	Nasopharyngeal carcinoma	Nasopharynx	Carcinoma	3	1.000	1.000
SW-1736	Thyroid gland undifferentiated (anaplastic) carcinoma	Thyroid	Carcinoma	80	0.987	0.987
SW-620	Colon adenocarcinoma	Colon	Adenocarcinoma	1845	0.887	0.887
SW1116	Colorectal Adenocarcinoma	Colon	Adenocarcinoma	46	0.864	0.867
SW1353	Bone chondrosarcoma	Bone	Sarcoma	41	0.938	0.938
SW1573	Lung carcinoma	Lung	Carcinoma	228	0.919	0.917
SW1990	Pancreatic adenocarcinoma	Pancreas	Adenocarcinoma	108	0.876	0.875
SW48	Colorectal Adenocarcinoma	Colon	Adenocarcinoma	101	0.806	0.809
SW480	Colon adenocarcinoma	Colon	Adenocarcinoma	846	0.932	0.932

SiHa	Cervical squamous cell carcinoma	Cervix	Carcinoma	28	0.859	0.851
Sku1B	Rb positve uterine leiomyosarcoma	Uterus	Sarcoma	6	1.000	1.000
T-24	Bladder carcinoma	Urinary tract	Carcinoma	297	0.925	0.923
T47D	Breast carcinoma	Breast	Carcinoma	461	0.936	0.934
T98	Glioblastoma	Brain	Blastoma	5	1.000	1.000
T98G	Glioblastoma	Brain	Carcinoma	21	0.912	0.912
TERT-RPE1	Retinal pigmented epithelial cell	Retina	Normal	31	0.942	0.942
TF-1	Bone marrow erythroleukemic	Haematopoietic and lymphoid tissue	Leukemia	34	0.972	0.972
THP-1	Acute monocytic leukemia	Blood	Leukemia	1482	0.937	0.934
TK-10	Renal carcinoma	Kidney	Carcinoma	731	0.854	0.851
TMD8	Diffuse large B-cell lymphoma activated B-cell type	Lymphocytes	Lymphoma	7	0.811	0.816
TOV112D	Ovarian endometrioid adenocarcinoma	Ovary	Carcinoma	4	0.998	0.998
TSGH9201	Gastric signet ring cell adenocarcinoma	Stomach	Carcinoma	18	1.000	1.000
TSU	Prostatic carcinoma	Prostate	Carcinoma	15	0.947	0.948
U-251	Astrocytoma	Brain	Astrocytoma	1478	0.883	0.882
U-266	Plasma cell myeloma	Blood	Myeloma	147	0.870	0.864
U-87MG	Glioblastoma	Brain	Blastoma	622	0.921	0.919
U-937	Histiocytic lymphoma	Haematopoietic and lymphoid tissue	Lymphoma	138	0.926	0.920
U-937/GTB	Histiocytic lymphoma	Blood	Lymphoma	10	0.846	0.852
U2OS	Osteosarcoma	Bone	Sarcoma	299	0.885	0.883
UACC-257	Melanoma	Skin	Melanoma	571	0.861	0.858
UACC-375	Melanoma	Skin	Melanoma	86	0.994	0.994
UACC-62	Melanoma	Skin	Melanoma	1217	0.904	0.902
UACC-903	Malignant melanoma	Skin	Melanoma	38	0.954	0.953
UCLAP-3	Lung squamous cell carcinoma	Lung	Carcinoma	5	1.000	1.000
UMSCC22B	Hypopharyngeal squamous cell carcinoma	Upper aerodigestive tract	Carcinoma	4	1.000	1.000
UMUC3	Bladder Carcinoma	Urinary tract	Carcinoma	86	0.810	0.806
UO-31	Renal carcinoma	Kidney	Carcinoma	745	0.879	0.879
VCaP	Prostate carcinoma	Prostate	Carcinoma	86	0.980	0.980
WI-38	Embryonic lung fibroblast	Lung	Normal	288	0.943	0.941
WIL2	Lymphoblastoid cell	Haematopoietic, lymphoid tissue	Normal	39	1.000	1.000
WIL2-NS	Lymphoblastoid cell	Haematopoietic, lymphoid tissue	Normal	49	0.979	0.979
WM 266-4	Melanoma	skin	Melanoma	17	0.977	0.977
WM164	Cutaneous melanoma	skin	Melanoma	18	0.964	0.964
WiDr	Adenocarcinoma; Colorectal	Colon	Carcinoma	23	0.893	0.896
XF498	Glioma	Brain	Glioma	98	0.965	0.964
Y79	Retinoblastoma	Eye; Retina	Blastoma	14	0.963	0.964
YAPC	Pancreatic carcinoma	Pancreas	Carcinoma	50	0.817	0.810
Z-138	Lymphoma; Mantle Cell	lymph nodes	Lymphoma	51	0.925	0.922
ZR-75-1	Breast carcinoma	Breast	Carcinoma	242	0.965	0.966

Number – number of active compounds; **LOO CV** – leave-one-out cross-validation; **20-Fold CV** – 20-fold cross-validation.
