# Supplementary material for: CLC-Pred 2.0: A Freely Available Web Application for In Silico Prediction of Human Cell Line Cytotoxicity and Molecular Mechanisms of Action for Druglike Compounds
Source: Int J Mol Sci. 2023 Jan 14;24(2):1689. doi: 10.3390/ijms24021689 (PMC9861947; doi:10.3390/ijms24021689)
Supplement: Supplementary file 1 [file ijms-24-01689-s001.zip › Table S2.pdf]

Table S2: The information related with description of the training set based on DTP NCI60 data and accuracy of cytotoxicity prediction for NCI60 cell lines.

DTP NCI60 cell-line	Tissue of origin	Age	Sex	Epithelial	Histology	Ploidy	p53	Number (1 nM)	AUC LOO CV (1 nM)	AUC 20-Fold CV (1 nM)	Number (10 nM)	AUC LOO CV (10 nM)	AUC 20-Fold CV (10 nM)	Number (100 nM)	AUC LOO CV (100 nM)	AUC 20-Fold CV (100 nM)
BR:BT-549	Breast	72	F	yes	Papillary infiltrating ductal carcinoma-mammary gland; breast	3n+/-, Near-triploid 69+/- (58-80)	NA	37	0.973	0.973	284	0.904	0.902	918	0.871	0.868
BR:HS 578T	Breast	74	F	yes	Carcinosarcoma-mammary gland; breast	2n+, Hyperdiploid (47-57)	MT	57	0.962	0.955	379	0.877	0.876	1117	0.855	0.853
BR:MCF7	Breast	69	F	yes	Adenocarcinoma-mammary gland; breast; metastatic site: pleural effusion;	3n-, Hypotriploid (58-68)	WT	74	0.913	0.904	739	0.905	0.904	1921	0.874	0.874
BR:MDA-MB-231	Breast	51	F	yes	Adenocarcinoma-mammary gland; breast; epithelial; metastatic site: pleural effusion;	2n+, Hyperdiploid (47-57)	MT	45	0.937	0.932	291	0.918	0.918	961	0.878	0.878
BR:T-47D	Breast	54	F	yes	infiltrating ductal carcinoma	2n+, Hyperdiploid (47-57)	MT	46	0.908	0.909	364	0.911	0.909	1058	0.874	0.874
CNS:SF-268	Central nervous system	24	F	no	Glioblastoma, ud	2n+, Hyperdiploid (47-57)	MT	44	0.956	0.956	410	0.920	0.920	1283	0.888	0.888
CNS:SF-295	Central nervous system	67	F	no	Glioblastoma, ud	5n+/-, Near-pentaploid 115+/- (104-126)	MT	62	0.966	0.964	499	0.905	0.905	1529	0.874	0.873
CNS:SF-539	Central nervous system	34	F	no	Glial cell neoplasm	4n+/-, Near-tetraploid 92+/- (81-103)	WT	55	0.955	0.948	519	0.892	0.891	1489	0.870	0.870
CNS:SNB-19	Central nervous system	47	M	no	Glioblastoma, ud	3n+/-, Near-triploid 69+/- (58-80)	MT	43	0.958	0.950	368	0.906	0.906	1087	0.882	0.881
CNS:SNB-75	Central nervous system	NA	F	no	Astrocytoma	2n+, Hyperdiploid (47-57)	MT	63	0.936	0.930	491	0.856	0.854	1506	0.851	0.851
CNS:U251	Central nervous system	75	M	no	Glioblastoma,ud	2n+, Hyperdiploid (47-57)	MT	59	0.963	0.957	530	0.913	0.913	1565	0.880	0.880
CO:COLO 205	Colon	70	M	yes	Adenocarcinoma	3n, Triploid (69)	MT	50	0.961	0.962	497	0.915	0.913	1498	0.881	0.880
CO:HCC-2998	Colon	NA	NA	yes	carcinoma	2n+/-, Near-diploid 46+/-	MT	38	0.948	0.949	381	0.890	0.888	1150	0.868	0.868

						(35-57)									
CO:HCT-116	Colon	NA	M	yes	carcinoma-vpd	2n-, Hypodiploid (35-45)	NA	72	0.958	0.952	621	0.911	0.910	1858	0.867	0.865
CO:HCT-15	Colon	NA	NA	yes	Adenocarcinoma p/md	2n+/-, Near-diploid 46+/- (35-57)	NA	31	0.955	0.950	336	0.898	0.896	1255	0.874	0.873
CO:HT29	Colon	44	F	yes	Adenocarcinoma-md	3n+/-, Near-triploid 69+/- (58-80)	MT	54	0.952	0.953	564	0.916	0.915	1573	0.886	0.884
CO:KM12	Colon	NA	NA	yes	Adenocarcinome-pd	2n+/-, Near-diploid 46+/- (35-57)	MT	55	0.956	0.944	443	0.912	0.911	1451	0.881	0.880
CO:SW-620	Colon	51	M	yes	Carcinoma-ud	2n+/-, Near-diploid 46+/- (35-57)	MT	61	0.963	0.959	569	0.901	0.900	1687	0.873	0.872
LC:A549/ATCC	Non-Small Cell Lung	58	M	yes	Adenocarcinoma-p/md	3n+/-, Near-triploid 69+/- (58-80)	WT	47	0.961	0.960	437	0.918	0.918	1371	0.883	0.881
LC:EKVX	Non-Small Cell Lung	NA	M	yes	Adenocarcinoma-md	3n+/-, Near-triploid 69+/- (58-80)	MT	29	0.931	0.932	219	0.900	0.900	804	0.853	0.850
LC:HOP-62	Non-Small Cell Lung	60	F	yes	adenocarcinoma-ud	4n+, Hypertetraploid (93-103)	MT	54	0.961	0.962	420	0.908	0.907	1344	0.881	0.880
LC:HOP-92	Non-Small Cell Lung	62	M	yes	Large cell-ud	4n+/-, Near-tetraploid 92+/- (81-103)	MT	34	0.936	0.919	398	0.822	0.819	1316	0.829	0.829
LC:NCI-H226	Non-Small Cell Lung	NA	M	yes	Squamous cell carcinoma-vpd	3n, Triploid (69)	MT	34	0.970	0.968	305	0.873	0.874	1088	0.867	0.866
LC:NCI-H23	Non-Small Cell Lung	NA	M	yes	Adenocarcinoma-ud	2n+, Hyperdiploid (47-57)	MT	45	0.950	0.939	436	0.914	0.914	1374	0.882	0.881
LC:NCI-H322M	Non-Small Cell Lung	52	M	yes	Small cell Bronchioalveolar Carcinoma	2n+/-, Near-diploid 46+/- (35-57)	MT	39	0.925	0.915	288	0.921	0.918	994	0.880	0.877
LC:NCI-H460	Non-Small Cell Lung	NA	M	yes	Large Cell Carcinoma-ud	2n+/-, Near-diploid 46+/- (35-57)	WT	69	0.947	0.939	679	0.912	0.913	1843	0.874	0.873
LC:NCI-H522	Non-Small Cell Lung	NA	M	yes	Adenocarcinoma-vpd	2n+/-, Near-diploid 46+/- (35-57)	MT	69	0.974	0.970	604	0.885	0.884	1824	0.866	0.864
LE:CCRF-CEM	Leukemia	4	F	no	ALL	2n+/-, Near-diploid 46+/- (35-57)	MT	68	0.937	0.935	755	0.868	0.867	2131	0.825	0.824
LE:HL-60(TB)	Leukemia	36	F	no	Pro myelocytic leukemia	2n+/-, Near-diploid 46+/- (35-57)	MT	78	0.955	0.953	724	0.844	0.843	2047	0.819	0.819

LE:K-562	Leukemia	53	F	no	CML	3n-, Hypotriploid (58-68)	MT	71	0.924	0.922	691	0.899	0.898	1974	0.857	0.856
LE:MOLT-4	Leukemia	19	M	no	ALL (cells were taken when patient was in relapse)	4n, Tetraploid (92)	WT	64	0.971	0.965	704	0.898	0.897	1910	0.863	0.863
LE:RPMI-8226	Leukemia	61	M	no	Myeloma	3n-, Hypotriploid (58-68)	WT	68	0.961	0.953	582	0.883	0.882	1665	0.846	0.845
LE:SR	Leukemia	11	M	no	Lymphoma	2n+/-, Near-diploid 46+/- (35-57)	NA	94	0.921	0.921	893	0.862	0.861	2425	0.828	0.827
ME:LOX IMVI	Melanoma	58	M	no	Malignant amelanotic melanoma	3n+/-, Near-triploid 69+/- (58-80)	WT	68	0.969	0.964	582	0.900	0.899	1740	0.865	0.864
ME:M14	Melanoma	NA	NA	no	Melanotic melanoma	3n+/-, Near-triploid 69+/- (58-80)	MT	56	0.950	0.941	479	0.909	0.909	1504	0.875	0.874
ME:MALME-3M	Melanoma	43	M	no	Malignant melanotic melanoma	4n+/-, Near-tetraploid 92+/- (81-103)	WT	36	0.908	0.894	339	0.892	0.892	1164	0.866	0.866
ME:MDA-MB-435	Melanoma	31	F	no	Ductal carcinoma-mammary gland; breast; duct; metastatic site: pleural effusion;	2n+, Hyperdiploid (47-57)	MT	82	0.944	0.938	564	0.908	0.907	1625	0.870	0.869
ME:MDA-N	Melanoma	31	F	no	Ductal carcinoma-mammary gland; breast; duct; metastatic site: pleural effusion;	NA	MT	60	0.939	0.932	289	0.941	0.942	678	0.910	0.909
ME:SK-MEL-2	Melanoma	60	M	no	Malignant melanotic melanoma	4n-, Hypotetraploid (81-91)	WT	39	0.968	0.968	329	0.909	0.907	998	0.882	0.880
ME:SK-MEL-28	Melanoma	51	M	no	Malignant melanotic melanoma	4n-, Hypotetraploid (81-91)	MT	29	0.967	0.962	217	0.909	0.907	755	0.878	0.878
ME:SK-MEL-5	Melanoma	24	F	no	Malignant melanotic melanoma	4n+, Hypertetraploid (93-103)	WT	56	0.971	0.966	464	0.909	0.910	1525	0.875	0.874
ME:UACC-257	Melanoma	NA	NA	no	Melanotic melanoma	3n+, Hypertriploid (70-80)	WT	28	0.972	0.971	269	0.896	0.892	936	0.875	0.875
ME:UACC-62	Melanoma	NA	NA	no	Melanotic melanoma	3n+/-, Near-triploid 69+/- (58-80)	WT	58	0.957	0.953	502	0.909	0.909	1573	0.872	0.871
OV:IGROV1	Ovarian	47	F	yes	Cystoadenocarcinoma-pd	4n+/-, Near-tetraploid 92+/- (81-103)	MT	35	0.950	0.949	443	0.908	0.906	1411	0.871	0.871
OV:NCI/ADR-RES	Ovarian	NA	F	yes	Adenocarinoma	2n+/-, Near-diploid 46+/- (35-57)	MT	28	0.877	0.868	264	0.903	0.900	981	0.886	0.884

OV:OVCAR-3	Ovarian	60	F	yes	Adenocarcinoma-md	3n+/-, Near-triploid 69+/-(58-80)	MT	56	0.948	0.938	458	0.907	0.904	1454	0.868	0.867
OV:OVCAR-4	Ovarian	42	F	yes	Adenocarcinoma-md	3n+/-, Near-triploid 69+/-(58-80)	WT	22	0.959	0.960	213	0.917	0.916	829	0.861	0.859
OV:OVCAR-5	Ovarian	67	F	yes	Adenocarcinoma-wd	2n+, Hyperdiploid (47-57)	MT	26	0.930	0.929	195	0.906	0.906	819	0.880	0.877
OV:OVCAR-8	Ovarian	64	F	yes	Carcinoma-ud	2n+, Hyperdiploid (47-57)	MT	41	0.961	0.948	436	0.908	0.906	1347	0.884	0.882
OV:SK-OV-3	Ovarian	64	F	yes	Adenocarcinoma-vpd	4n+/-, Near-tetraploid 92+/-(81-103)	NA	33	0.909	0.910	337	0.887	0.887	1135	0.883	0.882
PR:DU-145	Prostate	69	M	yes	prostate; metastatic site: brain; carcinoma (patient with metastatic carcinoma of the prostate and a 3 year history of lymphocytic leukemia.)	3n+/-, Near-triploid 69+/-(58-80)	NA	63	0.953	0.946	416	0.917	0.917	1218	0.876	0.875
PR:PC-3	Prostate	62	M	yes	Adenocarcinoma-prostate; metastatic site: bone;	4n, Tetraploid (92)	MT	57	0.943	0.936	428	0.907	0.907	1280	0.881	0.880
RE:786-0	Renal	58	M	yes	Adenocarcinoma	4n+/-, Near-tetraploid 92+/-(81-103)	MT	51	0.939	0.939	457	0.907	0.905	1384	0.878	0.876
RE:A498	Renal	52	F	yes	Adenocarcinoma	3n, Triploid (69)	WT	34	0.954	0.953	389	0.864	0.858	1453	0.861	0.860
RE:ACHN	Renal	22	M	yes	Renal cell carcinoma-p/md	2n+/-, Near-diploid 46+/-(35-57)	WT	45	0.936	0.934	464	0.908	0.908	1428	0.883	0.883
RE:CAKI-1	Renal	49	M	yes	Clear cell carcinoma	3n, Triploid (69)	WT	44	0.922	0.924	432	0.888	0.887	1356	0.866	0.864
RE:RXF 393	Renal	54	M	yes	hypernephroma-pd	3n+/-, Near-triploid 69+/-(58-80)	MT	49	0.953	0.952	414	0.866	0.866	1433	0.849	0.848
RE:SN12C	Renal	43	M	yes	Renal cell carcinoma-pd	3n, Triploid (69)	MT	39	0.970	0.959	415	0.903	0.901	1286	0.878	0.877
RE:TK-10	Renal	43	M	yes	Renal Spindle cell carcinoma	4n, Tetraploid (92)	MT	23	0.912	0.915	173	0.873	0.874	771	0.866	0.866
RE:UO-31	Renal	NA	F	yes	Renal cell carcinoma-vpd	2n+/-, Near-diploid 46+/-(35-57)	WT	27	0.854	0.855	273	0.860	0.861	1128	0.853	0.853

Number – number of active compounds; **LOO CV** – leave-one-out cross-validation; **20-Fold CV** – 20-fold cross-validation.
